# A natural allele of *OsMS1* responds to temperature changes and confers thermosensitive genic male sterility

**DOI:** 10.1038/s41467-022-29648-z

**Published:** 2022-04-19

**Authors:** Lunying Wu, Xiaohui Jing, Baolan Zhang, Shoujun Chen, Ran Xu, Penggen Duan, Danni Zou, Shengjian Huang, Tingbo Zhou, Chengcai An, Yuehua Luo, Yunhai Li

**Affiliations:** 1 College of Tropical Crops, Hainan University, Haikou, 570228 China; 2 State Key Laboratory of Plant Cell and Chromosome Engineering, CAS Centre for Excellence in Molecular Plant Science, Institute of Genetics and Developmental Biology, Chinese Academy of Sciences, Beijing, 100101 China; 3 The State Key Laboratory of Protein and Plant Gene Research, College of Life Sciences, Peking University, Beijing, 100871 China; 4 Shanghai Agrobiological Gene Center, Shanghai, 201106 China; 5 Sanya Nanfan Research Institute of Hainan University, Haikou, 570228 China; 6 Hainan Yazhou Bay Seed Laboratory, Sanya, 572025 China; 7 College of Advanced Agricultural Sciences, University of Chinese Academy of Sciences, Beijing, 100039 China; 8 The Innovative Academy of Seed Design, Chinese Academy of Sciences, Beijing, 100101 China

**Keywords:** Plant breeding, Plant molecular biology, Agricultural genetics

## Abstract

Changes in ambient temperature influence crop fertility and production. Understanding of how crops sense and respond to temperature is thus crucial for sustainable agriculture. The thermosensitive genic male-sterile (TGMS) lines are widely used for hybrid rice breeding and also provide a good system to investigate the mechanisms underlying temperature sensing and responses in crops. Here, we show that OsMS1 is a histone binding protein, and its natural allele *OsMS1*^*wenmin1*^ confers thermosensitive male sterility in rice. OsMS1 is primarily localized in nuclei, while OsMS1^wenmin1^ is localized in nuclei and cytoplasm. Temperature regulates the abundances of OsMS1 and OsMS1^wenmin1^ proteins. The high temperature causes more reduction of OsMS1^wenmin1^ than OsMS1 in nuclei. OsMS1 associates with the transcription factor TDR to regulate expression of downstream genes in a temperature-dependent manner. Thus, our findings uncover a thermosensitive mechanism that could be useful for hybrid crop breeding.

## Abstract (web summary)

Thermosensitive genic male-sterile (TGMS) lines are widely used for two-line hybrid rice breeding, but the mechanism of TGMS has not been fully elucidated. Here, the authors show that natural allele of the *OsMS1* gene, encoding a histone binding PHD finger protein, responds to temperature change and confers TGMS in rice.

## Introduction

As one of the most important cereal crops, rice (*Oryza sativa* L.) is a staple food for nearly half of the world’s population^1^. Hybrid rice leads to a 10–20% grain yield increase compared with parental inbred lines^2–4^, thereby contributing greatly to food security. The three-line system (the cytoplasmic male-sterile line [CMS], the maintainer line, and the restorer line) and two-line system (the thermosensitive genic male-sterile [TGMS] line/photoperiod-sensitive genic male-sterile [PGMS] line and the restorer line) are widely used for hybrid rice breeding^5,6^. TGMS lines are male-sterile at restrictive (high) temperatures but male-fertile at permissive (low) temperatures. Therefore, the male-sterile line in two-line system also serves as the maintainer line, resulting in a dramatically decreased cost in seed production. Considering that most cultivars can restore the fertility of TGMS lines^5,7,8^, two-line system utilizes broader genetic resources to generate hybrids than three-line system in hybrid rice breeding. Recently, TGMS lines have made tremendous contributions to heterosis breeding for increasing rice yield in China^2,5^.

As the male gametophyte development has been identified as the most heat-vulnerable stage in flowering plants^9^, high temperature often causes male sterility. TGMS lines therefore provide a good system for studying the tissue-specific thermosensitive mechanisms. A few factors conferring TGMS phenotype have been identified in rice, including RNase Z^S1^, long non-coding RNA, and receptor-like kinases, but temperature does not directly alter the properties (e.g., activity or protein abundance) of these factors^5,7,8^.

Male reproductive development is a dynamic fine-tuning regulatory process involving the formation of male meiocytes, gametophytes, and gametes^10^. The male reproductive organ, the anther, forms male gametophytes via meiosis and mitosis within the flower, and releases mature pollen grain^11^. Tapetum, the innermost anther wall layer, plays essential roles in supporting gametogenesis, and undergoes programmed cell death (PCD)-triggered degradation after the meiosis of microspore mother cells^10^. Thus, abnormal development and/or premature or delayed degradation of the tapetum results in male sterility. Several conserved receptor-like protein kinases and transcriptional regulators have been identified to be associated with tapetal cell development in rice^10^. Rice *MULTIPLE SPOROCYTE 1* (*MSP1*) and *MICROSPORELESS 2* (*MIL2*) encode receptor-like protein kinases and work coordinately to promote the correct progression of anther development^12,13^. Rice MYB transcription factor encoded by *GAMYB* plays an important role in floral organ development and pollen development^14,15^. Rice *UDT1* (*UNDEVELOPED TAPETUM 1*)^16^, *TDR* (*TAPETUM DEGENERATION RETARDATION*)^17^, *EAT1* (*ETERNAL TAPETUM 1*)^18^, and *TIP2/bHLH142* (*TDR INTERACTING PROTEIN 2*)^19,20^, encoding basic helix-loop-helix (bHLH) transcription factors, play key roles in maintaining tapetum development in rice. *TIP2/bHLH142* acts downstream of *UDT1* and *GAMYB* but upstream of *TDR* and *EAT1* in pollen development^18–20^. *PTC1* (*PERSISTENT TAPETAL CELL1*)/*OsMS1* (*MALE STERILITY 1*) encodes a transcriptional activator that regulates tapetal PCD and pollen exine formation in rice^21–23^. However, how *PTC1/OsMS1* is involved in the regulation of transcriptional activation remains unclear.

Here, we report that OsMS1 is a histone binding protein, and its natural allele *OsMS1*^*wenmin1*^ from two *indica* TGMS lines (Tian1S and HengnongS-1) confers thermosensitive genic male sterility in rice. The abundances of OsMS1 and OsMS1^wenmin1^ are temperature dependent. OsMS1 associates with the transcription factor TDR in nuclei to activate its downstream gene expression in a temperature-dependent manner. These findings define an important mechanism of thermosensitive genic male sterility control and open a way for hybrid crop breeding.

## Results

### The *wenmin1* allele confers thermosensitive male sterility in both *indica* and *japonica* backgrounds

To dissect the temperature sensing mechanism, we collected multiple temperature-sensitive male-sterile lines used for two-line hybrid rice breeding. Among them, the *indica* TGMS line Tian1S showed male sterility under high temperature (restrictive temperature), but exhibited normal male fertility under low temperature (permissive temperature), irrespective of the day length (Supplementary Fig. 1a, b). Our genetic analysis revealed that the thermosensitive male-sterile phenotype was controlled by a single recessive locus (Supplementary Fig. 1c, Supplementary Table 2), hereafter named this locus *wenmin1* (wenmin: temperature-sensitive in Chinese).

**Fig. 1:**
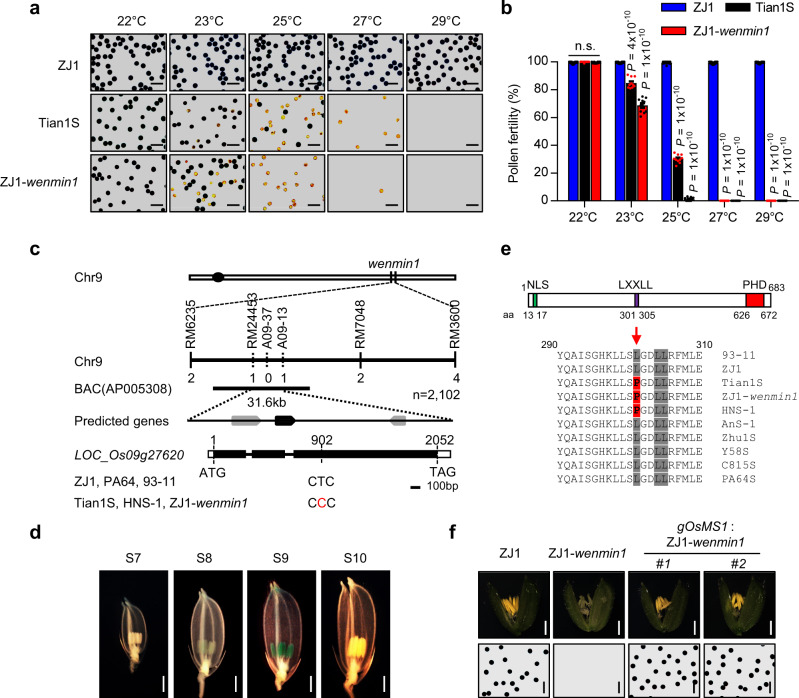
Phenotypes and identification of the *wenmin1* allele. **a** Pollen fertility of ZJ1, Tian1S and ZJ1-*wenmin1* at the indicated day average temperature (DAT). **b** Statistical analysis of (**a**). Data are presented as means ± s.e.m. (*n* = 10 biological replicates). *P* values indicate the significant differences relative to ZJ1 at the indicated temperatures. Two-tailed unpaired *t*-test was used for statistical analysis. n.s., not significant. **c** Mapping and identification of *wenmin1*. Arrows, predicted genes; black boxes, exons; black lines, introns. Numbers indicate the exon length. The specific single nucleotide polymorphism in Tian1S, HNS-1, and ZJ1-*wenmin1* was shown in red. **d** OsMS1-GUS is specifically detectable in the stages 8 and 9 anthers. S7, Stage 7; S8, stage 8; S9, stage 9; S10, stage 10. **e** Schematic diagram of OsMS1 protein structure. Green, purple and red rectangles indicate NLS, LXXLL motif, and PHD domain, respectively. aa, amino acids. The mutated amino acids (L301P) in Tian1S, HNS-1, and ZJ1-*wenmin1* are shaded in red. **f** Spikelet (up) and pollen (down) phenotypes of ZJ1, ZJ1-*wenmin1*, and *gOsMS1:*ZJ1-*wenmin1* lines (#1 and #2). Scale bars, 100 μm (**a**), 1 mm (**d**), 1 mm (**f**, up), 100 μm (**f**, down). Representative results of at least three independent experiments in (**d**) and (**f**) are shown. Source data are provided as a Source data file.

To explore whether the *wenmin1* allele could also confer the thermosensitive male sterility in *japonica* varieties, we backcrossed Tian1S with a wide compatibility *japonica* cultivar ZhongJing1 (ZJ1) and generated the nearly isogenic line ZJ1-*wenmin1*, which contains the *wenmin1* allele in ZJ1 background (Supplementary Fig. 2a, b). ZJ1-*wenmin1* exhibited normal vegetative growth (Supplementary Fig. 2b), but was temperature-sensitive male sterile, irrespective of the day length (Supplementary Fig. 2c). To investigate the temperature range of *wenmin1* in detail, we examined the pollen fertility of Tian1S and ZJ1-*wenmin1* at day average temperatures (DAT) 22 °C, 23 °C, 25 °C, 27 °C, and 29 °C under the fixed photoperiods. Pollen fertility of Tian1S and ZJ1-*wenmin1* was normal at 22 °C, whereas they exhibited a gradual abortion with increasing temperatures (Fig. 1a, b). Tian1S and ZJ1-*wenmin1* showed no mature pollen grains at 27 °C and 25 °C, respectively. Together, these results indicate that the *wenmin1* allele confers the temperature-sensitive male sterility in both *indica* and *japonica* cultivars at restrictive temperature and that the male sterility-fertility transition temperature of the *wenmin1* allele depends on genetic backgrounds.

We further examined anther development to characterize the cellular abnormalities of ZJ1-*wenmin1*. At restrictive temperature, the early development events of anthers and pollen grains of ZJ1-*wenmin1* were similar to those of ZJ1, whereas ZJ1-*wenmin1* tapetum appeared abnormally enlarged, and microspores became degenerated with few or no mature pollen grains compared with normal tapetal layer degradation and mature pollen grains formation of ZJ1 (Supplementary Fig. 2d).

### Identification of the *wenmin1* mutation

To identify the *wenmin1* mutation, we conducted a map-based cloning by crossing Tian1S with ZJ1. Fine mapping of the *wenmin1* mutation using 2102 F_2_ individuals narrowed the candidate region to a 31.6 kb interval between markers RM24453 and A09-13 (Fig. 1c). Interestingly, this mapping region contains three genes, and one of them (*LOC_Os09g27620*) is the *PTC1*/*OsMS1* gene^21,23^. Notably, mutations in several *PTC1/OsMS1* homologs including *Arabidopsis thaliana MS1* (*At*MS1)^24–27^, barley *MALE STERILITY 1* (*Hv*MS1)^28^, and maize *MALE STERILITY 7* (*Zm*MS7)^29^ resulted in the male sterility, although these mutations did not cause the temperature-sensitive male-sterile phenotype. Thus, *LOC_Os09g27620* was the most promising candidate gene. To identify the causal mutation, we firstly investigated the expression of *LOC_Os09g27620* in ZJ1 and ZJ1-*wenmin1*. We found that *LOC_Os09g27620* is mainly expressed in stages 8 and 9 of anther development (Supplementary Fig. 3a), consistent with the public data (Supplementary Fig. 3b, c)^30^. Similarly, OsMS1-GUS fusion proteins in *gOsMS1:GUS* transgenic plants were also detected in stages 8 and 9 of anther development (Fig. 1d). We observed that the transcript levels of *LOC_Os09g27620* were similar in both ZJ1 and ZJ1-*wenmin1* grown at restrictive temperature (Supplementary Fig. 3a), indicating that the mutation in ZJ1-*wenmin1* does not affect the expression of *LOC_Os09g27620*. We then sequenced the *LOC_Os09g27620* gene and found a nucleotide transition (T902C) in the second exon of *LOC_Os09g27620* in Tian1S and ZJ1-*wenmin1* compared with ZJ1 and *indica* cultivars 93-11 and Pei’Ai 64 (PA64) (Fig. 1c), which resulted in an amino acid change (Leu^301^ to Pro, L301P) (Fig. 1e). These results suggest that T902C mutation in *LOC_Os09g27620* might be responsible for the temperature-sensitive male-sterile phenotype of Tian1S and ZJ1-*wenmin1*. To confirm this, we conducted a genomic complementation assay. A genomic fragment of *LOC_Os09g27620* (*gOsMS1*) from 93-11 was transformed into ZJ1-*wenmin1*. Transgenic plants (*gOsMS1:*ZJ1-*wenmin1*) showed normal fertility under restrictive temperature (Fig. 1f and Supplementary Fig. 4). Thus, these results demonstrate that the T902C mutation in *LOC_Os09g27620/OsMS1* is responsible for the temperature-sensitive male sterile phenotype in Tian1S and ZJ1-*wenmin1*, and that the *wenmin1* allele is a weak allele. Therefore, the *wenmin1* allele was referred to as *OsMS1*^*wenmin1*^ thereafter.

To explore whether other TGMS lines could contain the *OsMS1*^*wenmin1*^ mutation, we sequenced the *OsMS1* gene in multiple TGMS lines. The *indica* thermosensitive male sterile line HengnongS-1 (HNS-1) was bred from a cross progeny between wild rice and *indica* cultivars, and has been widely used in two-line hybrid rice breeding since the 1990s in China (China Rice Data Center, http://www.ricedata.cn). Interestingly, genomic DNA sequencing showed that HNS-1, but not other TGMS lines, harbors the same mutation as Tian1S and ZJ1-*wenmin1* (Fig. 1c). Notably, the *tms9-1* was also mapped to the *OsMS1*^*wenmin1*^ region using an F_2_ population from a cross between HNS-1 and Minghui63 (MH63)^31^. The *OsMS1* was identified as the candidate gene for *TMS9-1*, although genomic complementation test has not been conducted^31^. Consistent with this, our allelic tests revealed that *OsMS1*^*wenmin1*^ was allelic to *tms9-1*, but not to *tms5* (Supplementary Table 5). We then analyzed the sequences of 4,726 rice genomes (http://ricevarmap.ncpgr.cn/v2) and found that none of these rice varieties contained the *OsMS1*^*wenmin1*^ mutation, indicating that *OsMS1*^*wenmin1*^ is a rare allele.

*OsMS1* and its homolog genes are shared in monocot and dicot species (Supplementary Figs. 5 and 6), and the Leu^301^ of OsMS1 is evolutionarily conserved (Supplementary Fig. 7), suggesting a promising common target to engineer TGMS lines in the plant kingdom.

### OsMS1 is a histone binding protein

**Fig. 2:**
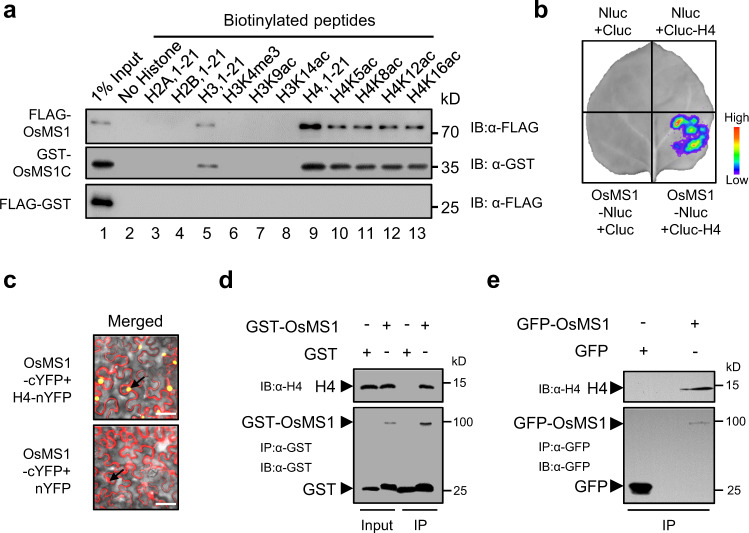
OsMS1 is a histone binding protein. **a** In vitro histone pull-down assay with the indicated proteins against biotinylated peptides. Proteins were pulled down by strepavidin beads and detected by western blots with antibody against FLAG or GST. **b** OsMS1 associates with H4 in a LCI assay. The indicated constructs were transiently expressed in *N. benthamiana* at 22 °C for 48 h. Luciferase activity is depicted with false color from low (blue) to high (red). Nluc, N-terminal half of firefly luciferase; Cluc, C-terminal half of firefly luciferase. **c** OsMS1 associates with H4 in a BiFC assay. Assays were done as in (**b**). nYFP, N-terminal portion of YFP; cYFP, C-terminal portion of YFP. Black arrows indicate nuclei. Scale bars, 50 μm. **d** OsMS1 associates with H4 in a *semi-*in vivo pull-down assay. GST-OsMS1 was incubated with nuclear proteins extracts from stage 9 anthers of rice young panicles. Proteins were pulled down by Glutathione sepharose beads and detected by western blots with antibody against GST or H4. **e** OsMS1 associates with H4 in a co-immunoprecipitation assay. Proteins extracts from stage 9 anthers of transgenic *35S:GFP-OsMS1* rice was immunoprecipitated with a GFP-Trap^®^_A agarose beads and detected by western blots with antibody against GFP or H4. *35S:GFP* transgenic rice was used as a negative control. Experiments were repeated more than three times with similar results. Source data are provided as a Source data file.

*OsMS1* encodes a 683-amino acid protein that contains a putative nuclear localization motif (NLS) at the N-terminus, a LXXLL motif (where L is leucine and X is any amino acid) in the middle region^32^, and a plant homeodomain (PHD) in the C-terminus (Fig. 1e and Supplementary Fig. 5). In animals, the PHD fingers were proposed to play crucial roles in transcriptional regulation and were known to recognize a subset of post-translational histone modifications, such as histone H3 trimethylated at Lys4 (H3K4me3), unmethylated tail of histone H3 (H3K4me0), histone H3 trimethylated at Lys9 (H3K9me3), and histone H3 or H4 acetylated at various lysine residues (H3/H4Kac)^33–35^. However, little is known about the function of tissue-specific expressed PHD proteins in plants^36^. Interestingly, sequence alignment revealed that the PHD domain of OsMS1 is highly conserved in OsMS1 and its homologs (Supplementary Fig. 8a, b), but with a unique amino acid composition (Supplementary Fig. 8b, c), possessing more acidic amino acid that is different from the known PHD domains of human BPTF and PHF2 that specifically recognize H3K4me2/3, of human AIRE and BHC80 that bind H3K4me0, of human DPF3b that recognizes H3K14ac (Supplementary Fig. 8b, c). We therefore asked whether OsMS1 could bind histone tails through its PHD finger. To answer this, we tested several biotin-labeled histone H2A, H2B, H3, and H4 peptides and modified histone peptides bearing methylations and acetylations on different residues in peptide pull-down assays. Unexpectedly, FLAG-tagged full-length OsMS1 preferentially binds to H4 (lane 9), H4 acetylated at lysines 5, 8, 12, 16 (lanes 10–13), and H3 (lane 5) (Fig. 2a, upper panel). Meanwhile, OsMS1^wenmin1^ also interacted with H4, acetylated H4, and H3 (Supplementary Fig. 9a), indicating that the mutation in OsMS1^wenmin1^ does not affect its histone-binding ability. A glutathione S-transferase (GST) tagged C-terminal region of OsMS1 containing the PHD domain (GST-OsMS1C; amino acids 626–683) retains the histone-binding specificity of the full-length OsMS1 (Fig. 2a, middle panel), indicating that the PHD domain-bearing C-terminal region of OsMS1 is sufficient for binding these histone peptides. We further mutated the conserved C642, C645, H650, and C653, which are important for PHD domain structure, into Ala (Supplementary Fig. 8b). We found that the mutations (C642A, C645A, H650A, and C653A) within the PHD domain abolished the interactions between OsMS1 and histone peptides (Supplementary Fig. 9b), indicating that the PHD domain structure is crucial for the specific interaction. We confirmed the interaction between full-length OsMS1 and H4 in planta using a luciferase complementation imaging (LCI) assay and a bimolecular fluorescence complementation (BiFC) assay in *N. benthamiana* leaves (Fig. 2b, c). The association of OsMS1 with H4 in rice anthers is further demonstrated by the *semi*-in-vivo pull-down and in vivo co-immunoprecipitation assays (Fig. 2d, e). Together, these results indicate that OsMS1 is a histone binding protein.

### The L301P mutation in OsMS1^wenmin1^ affects its nucleus localization

**Fig. 3:**
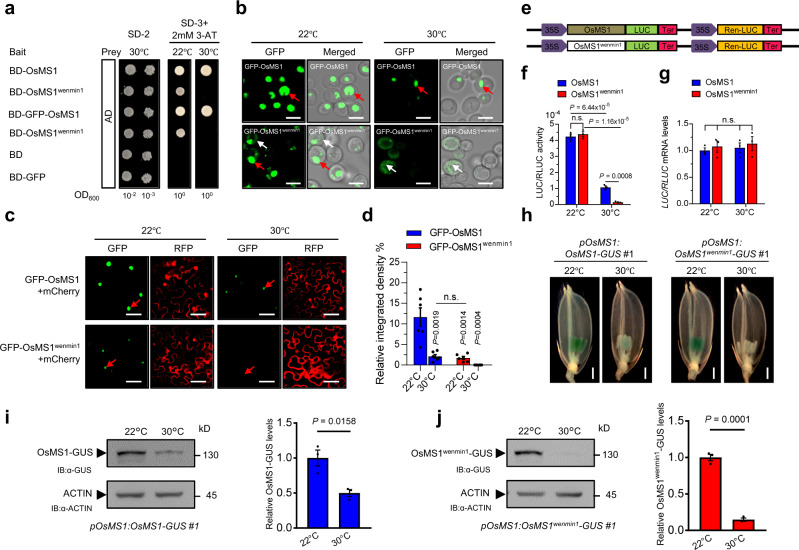
Temperature regulates the abundances of OsMS1 and OsMS1^wenmin1^. **a** Transcriptional activation assay in yeast. Yeast cells were spotted on the indicated plates and grown at low (22 °C) or high (30 °C) temperatures for 96 h. BD, binding domain; AD, activation domain; SD-2, plates lacking Trp and Leu; SD-3 + 2 mM 3-AT, plates lacking Trp, Leu, and His, plus 2 mM 3-aminotriazole. **b**, **c** Protein levels of GFP-OsMS1 and GFP-OsMS1^wenmin1^ are temperature-dependent in yeast cells (**b**) and *N. benthamiana* (**c**). *N. benthamiana* were incubated at 22 °C for 44 h and then transferred to the indicated temperatures for another 4 h before detecting the GFP signals. Red arrows indicate nuclei, white arrows indicate cytoplasm. **d** Quantitative analysis of (**c**). The GFP-OsMS1 and GFP-OsMS1^wenmin1^ protein levels were quantitated and normalized to the mCherry level. *P* values indicate the significant differences relative to GFP-OsMS1 at 22 °C. **e** Dual-luciferase system. LUC, firefly luciferase. Ren-LUC, Renilla luciferase. **f** Relative luciferase activity of OsMS1-LUC and OsMS1^wenmin1^-LUC in *N. benthamiana* plants during the low temperature to high-temperature transition. **g** *OsMS1* and *OsMS1*^*wenmin1*^ mRNA levels as in (**f**). **h**–**j** Protein levels of OsMS1-GUS and OsMS1^wenmin1^-GUS are temperature-dependent in rice. *pOsMS1:OsMS1-GUS* and *pOsMS1:OsMS1*^*wenmin1*^*-GUS* transgenic plants were grown at 22 °C or 30 °C in growth chamber. Protein levels of OsMS1-GUS and OsMS1^wenmin1^-GUS were detected by GUS-staining of transgenic plant spikelets at stage 9 (**h**). OsMS1-GUS (**i**, left) and OsMS1^wenmin1^-GUS (**j**, left) proteins were detected by ani-GUS antibody. Quantifications of OsMS1-GUS (**i**, right) and OsMS1^wenmin1^-GUS (**j**, right) protein levels were relative to ACTIN. Scale bars, 5 μm (**b**), 50 μm (**c**), 1 mm (**h**). In **d**, **f, g**, **i** (right) and **j** (right). Data are presented as means ± s.e.m. (*n* = 6 biological replicates for **d**, *n* = 3 biological replicates for **f**, **g**, **i**, and **j**). Two-tailed unpaired *t*-test was used for statistical analysis. n.s., not significant. Source data are provided as a Source data file.

Given the putative nuclear localization motif in OsMS1 (Fig. 1e and Supplementary Fig. 5) and the fact that the LXXLL motif was proposed to play a role in determining nuclear localization of Dax-1 (dosage-sensitive sex reversal-adrenal hypoplasia congenita critical region on the X chromosome, gene 1)^37,38^, we asked whether the L301P mutation in the conserved LXXLL motif of OsMS1 could influence its subcellular localization. We observed that the fluorescent signal of green fluorescent protein (GFP)-OsMS1 fusion protein was exclusively found in the nuclei in transgenic rice (Supplementary Fig. 10a), yeast (Fig. 3b and Supplementary Fig. 10b, upper panels), *N. benthamiana* (Supplementary Fig. 10c, upper panels), *Arabidopsis* (Supplementary Fig. 10d, upper panels), and rice protoplast (Supplementary Fig. 10e, upper panels), whereas GFP- OsMS1^wenmin1^ is distributed in both the cytoplasm and nuclei (Fig. 3b, Supplementary Fig. 10b, lower panels, Supplementary Fig. 10c, middle and lower panels, Supplementary Fig. 10d, lower panels, Supplementary Fig. 10e, lower panels). To validate these results, we then investigated the localization of OsMS1 and OsMS1^wenmin1^ using stable rice transgenic plants expressing OsMS1-FLAG and OsMS1^wenmin1^-FLAG under native *OsMS1* promoter. Consistent with the results in yeast, *N. benthamiana,* and *Arabidopsis*, we found that OsMS1 was exclusively detected in the nuclei, while OsMS1^wenmin1^ was detected in both the cytoplasm and nuclei (Supplementary Fig. 10f). Together, these results indicate that the residue at position 301 (L301) is important for the nuclear localization of OsMS1, and also suggest that the L301P mutation affects the function of OsMS1, at least in part, by influencing its subcellular localization.

### Temperature regulates the abundances of OsMS1 and OsMS1^wenmin1^

Besides the histone binding domain, LXXLL motif also functions in transcriptional regulation in mammals^32,39^. Notably, the L301P substitution in OsMS1^wenmin1^ happens in the extremely conserved LXXLL motif (Fig. 1e and Supplementary Fig. 7). We therefore tested whether the L301P mutation in OsMS1 influences its transcriptional activation activity in yeast cell. Using BD-OsMS1 and BD-GFP-OsMS1 as baits and AD as prey, we found that OsMS1 and GFP-OsMS1 had the transcriptional activation activity in yeast cells (Fig. 3a). Furthermore, the PHD domain-bearing C-terminal region, but not the N-terminus, is necessary for the transcriptional activation of OsMS1 (Supplementary Fig. 11). Interestingly, we observed that yeast cells with BD-OsMS1^wenmin1^ grew slowly at 22 °C, but had no obvious growth at 30 °C (Fig. 3a and Supplementary Fig. 12a), indicating a temperature-dependent transcriptional activation activity of OsMS1^wenmin1^ in yeast cells. These results suggest that the L310P mutation in OsMS1^wenmin1^ could suppress the transcriptional activation activity and/or decrease its protein abundance in a temperature-dependent manner. Consistent with this hypothesis, we observed that OsMS1 and OsMS1^wenmin1^ protein abundance is sensitive to ambient temperatures in yeast cells (Fig. 3b). Compared with OsMS1, high temperature caused less OsMS1^wenmin1^ accumulation in the nuclei (Fig. 3b and Supplementary Fig. 12b). Thus, it is possible that the L301P mutation in OsMS1^wenmin1^ reduces its protein abundance in the nuclei, thereby decreasing the transcriptional activation activity in yeast cells, which is consistent with the fact that GFP-OsMS1 was detected in nuclei, while GFP-OsMS1^wenmin1^ was distributed in both nuclei and cytoplasm (Fig. 3b and Supplementary Fig. 10b). However, we could not fully exclude the possibility that the L301P mutation may directly repress the transcriptional activation activity in yeast cells.

We next asked whether the abundances of OsMS1 and OsMS1^wenmin1^ proteins are temperature-dependent in planta. We examined GFP-OsMS1 and GFP-OsMS1^wenmin1^ protein levels using a transient expression assay in *N. benthamiana*. GFP signals of GFP-OsMS1 and GFP-OsMS1^wenmin1^ were readily detectable at 22 °C, but became less abundant at 30 °C (Fig. 3c, d), although their transcript levels were similar (Supplementary Fig. 13a). Notably, GFP signals of GFP-OsMS1^wenmin1^ in nuclei were obviously weaker than that of GFP-OsMS1 in nuclei (Fig. 3c, d and Supplementary Fig. 10c). We then quantified protein levels of OsMS1 and OsMS1^wenmin1^ in response to ambient temperature changes using a dual-luciferase system (Fig. 3e). Surprisingly, we found that total luciferase activity of OsMS1-LUC and OsMS1^wenmin1^-LUC at 22 °C was similar (Fig. 3f). Considering that the mutation in OsMS1^wenmin1^ affects the distribution of OsMS1^wenmin1^ in cytoplasm and nuclei, the luciferase activity of OsMS1^wenmin1^-LUC in nuclei should be lower than that of OsMS1-LUC in the nuclei at 22 °C. The total luciferase activity of OsMS1^wenmin1^-LUC was significantly lower than that of OsMS1-LUC at 30 °C (Fig. 3f). Importantly, the luciferase activity of both OsMS1-LUC and OsMS1^wenmin1^-LUC was decreased at 30 °C compared with that at 22 °C (Fig. 3f), although *OsMS1-LUC* and *OsMS1*^*wenmin1*^*-LUC* transcripts were not significantly affected by temperatures (Fig. 3g). We further examined OsMS1 and OsMS1^wenmin1^ proteins using *pOsMS1:OsMS1-GUS* and *pOsMS1:OsMS1*^*wenmin1*^*-GUS* transgenic rice plants grown at permissive or restrictive temperatures. Consistently, we found that GUS signals in both *pOsMS1:OsMS1-GUS* and *pOsMS1:OsMS1*^*wenmin1*^*-GUS* transgenic lines were detectable at 22 °C, but became less apparent at 30 °C (Fig. 3h and Supplementary Fig. 14a). Notably, GUS signals in *pOsMS1:OsMS1*^*wenmin1*^*-GUS* transgenic lines were barely detected at 30 °C (Fig. 3h, Supplementary Fig. 14a), although transcript levels of *OsMS1* and *OsMS1*^*wenmin1*^ in these transgenic lines were similar (Supplementary Fig. 13b–d). We then quantified protein levels of OsMS1-GUS and OsMS1^wenmin1^-GUS using a GUS antibody. Consistently, we found that the abundances of OsMS1-GUS and OsMS1^wenmin1^-GUS proteins were obviously lower at 30 °C than those at 22 °C, and that the abundance of OsMS1^wenmin1^-GUS was barely detected at 30 °C (Fig. 3i, j and Supplementary Fig. 14b). To further examine the tissue expression pattern, we conducted transverse section analysis using stage 9 anthers of *pOsMS1:OsMS1-GUS* and *pOsMS1:OsMS1*^*wenmin1*^*-GUS* transgenic plants grown at 22 °C. Consistent with the previous results^21^, we found that OsMS1-GUS was expressed in tapetum (Supplementary Fig. 14c). Notably, L301P mutation in OsMS1 did not affects its tapetum expression at 22 °C (Supplementary Fig. 14c). Taken together, these results indicate that ambient temperature affects the abundances of OsMS1 and OsMS1^wenmin1^, and OsMS1^wenmin1^ is more sensitive to temperature changes than OsMS1.

### The interactions of OsMS1 and OsMS1^wenmin1^ with TDR are temperature-dependent

**Fig. 4:**
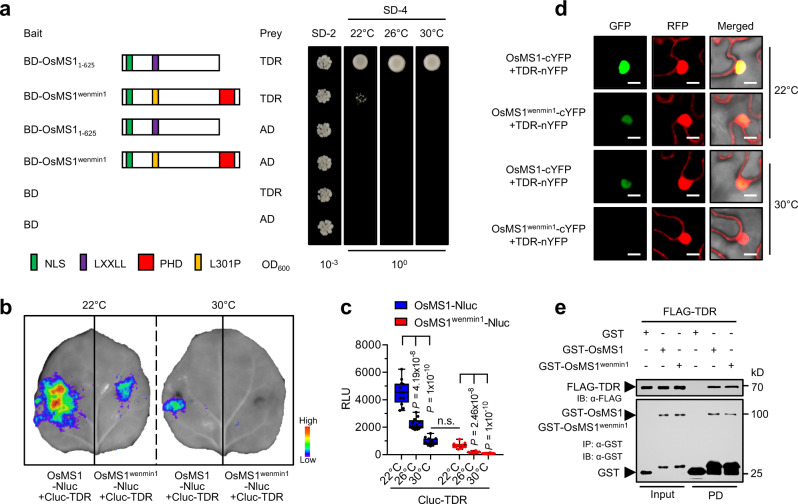
The interactions of OsMS1 and OsMS1^wenmin1^ with TDR in a temperature-dependent manner. **a** Temperature-dependent association of OsMS1^wenmin1^ and TDR in yeast. Yeast cells (OD_600_ = 1) were spotted on the SD-4 plates and grown at 22 °C, 26 °C, or 30 °C for 96 h. **b** Temperature-dependent associations of OsMS1 and OsMS1^wenmin1^ with TDR in LCI assays in *N. benthamiana* plants. *N. benthamiana* plants infiltrated with the *Agrobacteria* combinations were incubated at 22°C for 44 h and then transferred to the indicated temperatures for another 4 h. **c** Quantitative analysis of (**b**). Box-and-whisker plots display the minimum and maximum, the 25th and 75th percentiles (box), and median (center line). RLU, relative luminescence unit. Data are presented as mean RLU ± s.e.m. (*n* = 12 biological replicates). Two-tailed unpaired *t*-test was used for statistical analysis. n.s., not significant. **d** Temperature-dependent associations of OsMS1 and OsMS1^wenmin1^ with TDR in BiFC assays in *N. benthamiana* plants. nYFP, N-terminal portion of YFP; cYFP, C-terminal portion of YFP. Scale bars, 1 μm. **e** OsMS1 and OsMS1^wenmin1^ interact with TDR in a GST pull-down (PD) assay. The proteins were detected by western blots with antibody against GST or FLAG. Experiments in (**e**) were repeated three times with similar results. Source data are provided as a Source data file.

Considering that OsMS1 has the transcriptional activation activity (Fig. 3a) and lacks a predicted DNA-binding domain (Fig. 1e and Supplementary Fig. 5), it is possible that OsMS1 could interact with transcription factors to regulate gene expression. To identify the potential OsMS1-interacting proteins, we used the N-terminal fragment of OsMS1 (OsMS1_1-625_) as the bait to screen a yeast cDNA library derived from rice inflorescence, because the full-length OsMS1 had a self-activating activity. Interestingly, three independent clones corresponding to TDR were identified in this screen. We further confirmed the interaction of OsMS1_1-625_ with the full-length TDR in yeast cells (Fig. 4a). We then observed the interaction between full-length OsMS1 and TDR in planta using a LCI assay in *N. benthamiana* leaves (Fig. 4b, c and Supplementary Fig. 15a, b). Furthermore, bimolecular fluorescence complementation (BiFC) assays in *N. benthamiana* leaves revealed that OsMS1 associates with TDR in the nuclei (Fig. 4d), where OsMS1 and TDR were colocalized (Supplementary Fig. 16). Consistently, co-immunoprecipitation assays showed that OsMS1 associates with TDR in transgenic rice (Supplementary Fig. 15c). The pull-down assay using in vitro translated GST-tagged OsMS1 and FLAG-tagged TDR further demonstrated a direct interaction between OsMS1 and TDR (Fig. 4e). Supporting this, *OsMS1/PTC1* and *TDR* were spatio-temporally co-expressed (Supplementary Figs. 3, 16, and 17), and 40.3% of the altered genes in the *tdr* mutant had expression changes in the *ptc1* mutant^17,21^.

Considering that *OsMS1*^*wenmin1*^ contributes to temperature-sensitive male sterility, we next asked whether OsMS1 and OsMS1^wenmin1^ could interact with TDR in a temperature-dependent manner. Yeast cells cotransfacted AD with BD-OsMS1 but not BD-OsMS1^wenmin1^ can grow in SD-4 plates (Supplementary Fig. 11). Using BD-OsMS1^wenmin1^ as bait and AD-TDR as prey, yeast growth was observed on SD-4 plates incubated at 22 °C (Fig. 4a), albeit at a lower level. However, we did not observe yeast growth on SD-4 plates incubated at 26 °C or 30 °C (Fig. 4a). These results indicate that the temperature influences the abundance of OsMS1^wenmin1^, thereby resulting in the reduced interaction between OsMS1^wenmin1^ and TDR. Similarly, the temperature-dependent interaction between OsMS1^L304L305AA^ with mutations in LXXLL motif and TDR was also observed (Supplementary Fig. 18). We further used LCI assays to examine the interactions of OsMS1 and OsMS1^wenmin1^ with TDR in planta. We found that the abundance of the OsMS1^wenmin1^-TDR complex was temperature-dependent, and was also significantly lower than that of the OsMS1-TDR complex at both low and high temperatures (Fig. 4b, c and Supplementary Fig. 19a). Notably, we also observed that the interaction between OsMS1 and TDR was gradually decreased with increasing temperatures (Fig. 4b, c and Supplementary Fig. 19a), although the temperature did not obviously affect the interaction between OsMS1_1-625_ and TDR in yeast cells (Fig. 4a). Furthermore, BiFC assays confirmed the temperature-dependent interactions of OsMS1 and OsMS1^wenmin1^ with TDR in the nuclei (Fig. 4d and Supplementary Fig. 19b). Considering that high temperature reduced the levels of OsMS1 and OsMS1^wenmin1^ proteins, and that the abundance of TDR proteins is not affected by the temperatures (Supplementary Fig. 20), it is possible that the temperature-dependent interactions of OsMS1 and OsMS1^wenmin1^ with TDR are due to the temperature-dependent abundances of OsMS1 and OsMS1^wenmin1^ in the nuclei. Supporting this idea, in vitro pull-down assay showed that OsMS1 and OsMS1^wenmin1^ had similar ability to bind TDR (Fig. 4e).

### The OsMS1-TDR complex activates *EAT1* expression

**Fig. 5:**
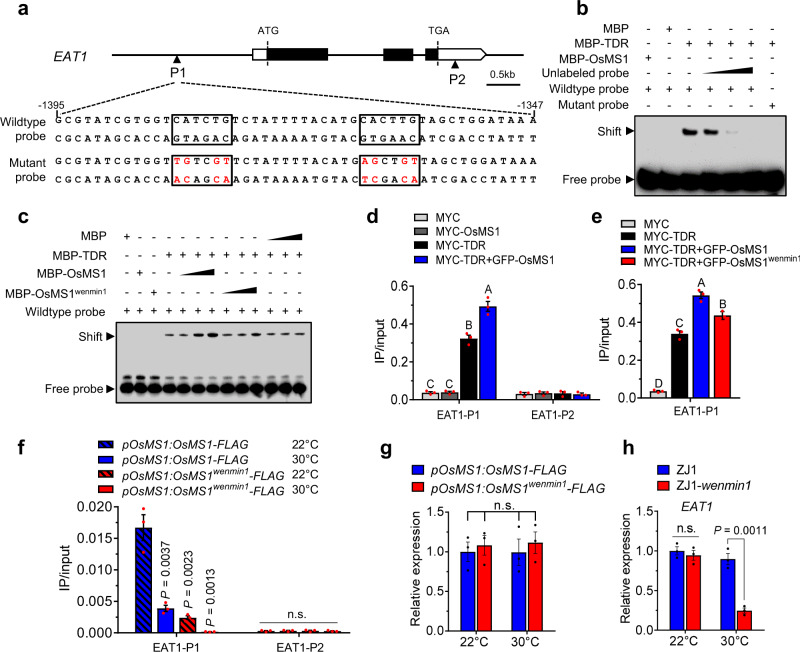
OsMS1 enhances the binding of TDR to the *EAT1* promoter. **a** Schematic representation of partial structure of the *EAT1* locus. The conserved E-box elements (CANNTG) are shown in black boxes. The mutated sequences are shown in red. P1 and P2 represent DNA fragments used for ChIP-qPCR analysis. P1 fragment contains two E-box elements, while P2 fragment contains no E-box element. **b** EMSA assay reveals that TDR but not OsMS1 directly binds to the *EAT1* promoter in vitro. The wild-type and mutated probes were shown in (**a**). MBP was used as a negative control. 10-, 20-,100-fold excess unlabeled probes were used as competitors. **c** EMSA assay showing the effects of OsMS1 and OsMS1^wenmin1^ on the binding of TDR to the E-box elements in the *EAT1* promoter in vitro. Biotin-labeled probes were incubated with a constant amount of MBP-TDR protein with increasing amounts of MBP-OsMS1, MBP-OsMS1^wenmin1^ or MBP. MBP was used as a negative control. **d**, **e** ChIP-qPCR assays showing the effects of OsMS1 and OsMS1^wenmin1^ on the binding of TDR to the *EAT1* promoter in rice leaf protoplasts (*n* = 3 biological repeats). Chromatins were incubated with anti-MYC antibody, and precipitated by protein A + G magnetic beads. ChIP-qPCR results were quantified by normalization of the MYC-IP signal with the corresponding input signal (IP/input), and different letters represent significant differences (*P* < 0.01; Duncan’s multiple range test). **f** Temperature-dependent enrichment of OsMS1 and OsMS1^wenmin1^ at the *EAT1* promoter by ChIP assays in rice stage 9 anthers. *pOsMS1:OsMS1-FLAG* and *pOsMS1:OsMS1*^*wenmin1*^*-FLAG* transgenic lines were grown at 22 °C or 30 °C in growth chamber. Chromatins were incubated with anti-FLAG antibody, and precipitated by protein A + G magnetic beads. ChIP-qPCR results were quantified by normalization of the FLAG-IP signal with the corresponding input signal (IP/input). *P* values indicate the significant differences relative to *pOsMS1:OsMS1-FLAG* at 22 °C. **g** mRNA levels of *OsMS1-FLAG* and *OsMS1*^*wenmin1*^*-FLAG* in (**f**). **h** qPCR analysis of *EAT1* expressions in ZJ1 and ZJ1-*wenmin1* stage 9 anthers. ZJ1 and ZJ1-*wenmin1* were grown at 22 °C or 30 °C in growth chamber. For **d**–**h**, values are means ± s.e.m. (*n* = 3 biological repeats). For **f**–**h**, two-tailed unpaired *t*-test was used for statistical analysis. n.s., not significant. Experiments in **b** and **c** were repeated three times with similar results. Source data are provided as a Source data file.

To identify the downstream factors of the OsMS1-TDR complex in regulating male fertility, we examined the expression levels of several genes involved in male fertility regulation at different stages of anther development^10^. We found that expression levels of *EAT1*, *CP1*, and *C6* in ZJ1-*wenmin1* were significantly reduced in comparison to those in ZJ1 under restrictive temperature (Supplementary Fig. 21). Importantly, expression of *EAT1*, *CP1*, and *C6* was also downregulated in *tdr* mutant^17,19^, supporting that OsMS1 and TDR share common downstream targets. TDR has been known to bind E-box elements (CANNTG) to activate gene expression^17^. Although *EAT1* has been proposed to act downstream of TDR to regulate tapetal development, and mutation in *TDR* resulted in the decreased expression of *EAT1*^18^, it is unclear whether TDR could directly bind the promoter of *EAT1*. To test this, we examined the promoter sequence of *EAT1* and found putative TDR binding elements (CANNTG) (Fig. 5a). We subsequently performed electrophoretic mobility shift assays (EMSAs) to test whether TDR could bind the E-boxes in the *EAT1* promoter. We expressed MBP-conjugated OsMS1 (MBP-OsMS1), TDR (MBP-TDR), or MBP in *Escherichia coli*. We found that MBP-TDR but not MBP-OsMS1 alone can bind directly to the promoter of *EAT1* in vitro (Fig. 5b). Moreover, excess unlabeled probe can compete with the corresponding labeled probe and eliminate the observed shift (Fig. 5b), indicating the specificity of the interaction. These data support a direct interaction between TDR and the promoter of *EAT1* via E-box elements. We further investigated the effects of OsMS1 and OsMS1^wenmin1^ on the binding of TDR to the E-box elements in the *EAT1* promoter. We found that OsMS1 enhances TDR’s binding to the *EAT1* promoter (Fig. 5c). Notably, OsMS1^wenmin1^ exhibited similar but a bit weaker ability than OsMS1 to enhance TDR’s binding to the *EAT1* promoter (Fig. 5c).

Given the fact that *OsMS1* and *TDR* are tapetum specific expressed genes (Supplementary Figs. 3)^17,21^, we performed chromatin immunoprecipitation (ChIP)-qPCR assays in rice leaf protoplasts to investigate the effect of OsMS1 on the binding of TDR to the *EAT1* promoter. We transiently expressed MYC-OsMS1 and MYC-TDR in rice protoplasts and detected the association of OsMS1 and TDR with the *EAT1* promoter. ChIP-qPCR results showed that MYC-TDR, but not MYC-OsMS1 alone associated with the *EAT1* promoter (Fig. 5d and Supplementary Fig. 22a). When MYC-OsMS1 was co-expressed with GFP-TDR, MYC-OsMS1 associated with the *EAT1* promoter (Supplementary Fig. 22c, d), indicating that the association of OsMS1 with the *EAT1* promoter depends on the TDR. In addition, we observed that OsMS1 enhanced the association of TDR with the *EAT1* promoter, and OsMS1^wenmin1^ exhibited similar but a bit weaker ability than OsMS1 to enhance the association of TDR with the *EAT1* promoter in rice protoplasts (Fig. 5e and Supplementary Fig. 22b).

Finally, we performed ChIP-qPCR using *pOsMS1:OsMS1-FLAG* and *pOsMS1:OsMS1*^*wenmin1*^*-FLAG* transgenic rice plants to test if OsMS1 and OsMS1^wenmin1^ were enriched at the promoter region of *EAT1* at different temperatures. We found that both OsMS1 and OsMS1^wenmin1^ were enriched more at the *EAT1* promoter at 22 °C than 30 °C (Fig. 5f, g). Notably, OsMS1^wenmin1^ was barely enriched at the *EAT1* promoter region at 30 °C compared with OsMS1 (Fig. 5f, g). We further examined the expression of *EAT1* in ZJ1 and ZJ1-*wenmin1* at different temperatures. We found that the expression of *EAT1* in ZJ1 and ZJ1-*wenmin1* were comparable at 22 °C, while expression of *EAT1* was significantly decreased in ZJ1-*wenmin1* at 30 °C compared with that in ZJ1 (Fig. 5h), indicating that *OsMS1*^*wenmin1*^ influences the expression of *EAT1* in a temperature-dependent manner.

## Discussion

Male sterile lines are widely used for crop hybrid breeding, contributing greatly to food security. In rice, CMS lines and TGMS/PGMS lines are extensively utilized to explore hybrid vigor in three-line system and two-line system, respectively, which dramatically increase rice grain yield. As male reproduction is temperature-vulnerable in flowering plants^9^, rice TGMS lines give a good system to understand how plants sense and respond to changing ambient temperature. In this study, we discovered that the natural allele *OsMS1*^*wenmin1*^ confers temperature-sensitive male sterility in rice, and OsMS1 is a PHD-finger protein that interacts with histones. Consistent with its role, OsMS1 is localized in nuclei. By contrast, OsMS1^wenmin1^ is localized in both cytoplasm and nuclei, indicating that the mutation in OsMS1^wenmin1^ influences its subcellular localization. We further found that temperatures regulate the abundances of OsMS1 and OsMS1^wenmin1^, and OsMS1^wenmin1^ is more sensitive to temperature changes than OsMS1. At permissive temperatures, the wild-type and the *OsMS1*^*wenmin1*^ allele possess suitable levels of OsMS1 and OsMS1^wenmin1^ proteins to keep normal pollen development, respectively, although the level of OsMS1^wenmin1^ in the *OsMS1*^*wenmin1*^ allele is lower in nuclei than that of OsMS1 in the wild type. Both OsMS1 and OsMS1^wenmin1^ interact with the transcription factor TDR to activate the expression of downstream genes, thereby generating fertile pollens. At restrictive temperatures, the abundance of OsMS1 proteins was decreased, but there are still enough OsMS1 proteins in nuclei to interact with TDR to activate the expression of downstream genes, therefore producing fertile pollens. In contrast, the high temperatures much strongly reduce the OsMS1^wenmin1^ protein levels, thus there are no enough OsMS1^wenmin1^ proteins in nuclei to interact with TDR, resulting in a dramatic decrease of downstream gene expression and forming sterile pollens. Thus, our findings uncover an important thermosensitive mechanism by which OsMS1 interacts with the transcription factor to control male fertility in a temperature-dependent manner (Supplementary Fig. 23).

As sessile organisms, plant cells must be capable of adapting to various environmental signals, especially for ambient temperature^40–46^. In plants, several proteins have been described to respond to ambient temperature^40,41,45,46^. Factors that sense and respond to temperature have been proposed to exist in anywhere in the cell^47^, and different tissues may also possess distinct mechanisms to detect temperature changes^43,44,47^. We found that temperature controls the abundance of OsMS1, which is specifically expressed in anthers. The natural allele *OsMS1*^*wenmin1*^ causes temperature-dependent male sterility. Thus, our results indicate that temperature-dependent abundance of OsMS1 in nuclei is crucial for tissue-specific response to temperature changes.

The LXXLL motif was originally reported to be a signature sequence that mediates the interactions of different proteins with nuclear receptors, and was proposed to be a defining feature of a new family of nuclear proteins in human^48^. In animals, it was reported that LXXLL motifs influence nuclear localizations by interacting with nuclear proteins^32,38^. For example, the LXXLL motif in the N-terminal repeat region of Dax-1 plays a crucial role in its nuclear localization through interacting with the nuclear protein Ad4BP/SF-1^38^. Interestingly, the L301P mutation in the LXXLL motif of OsMS1^wenmin1^ affects its subcellular localization. It will be interesting to investigate whether OsMS1 could interact with nuclear proteins to determine its nuclear localization and why the L301P mutation affects the nuclear localization of OsMS1^wenmin1^ in the future.

LXXLL motifs are present in many transcription factors and cofactors, and involved in activating or repressing transcription^32^. The L301P mutation in the LXXLL motif of OsMS1^wenmin1^ decreases its transcriptional activity (Fig. 3a, Supplementary Figs. 11 and 12a). Considering that the L301P mutation caused the reduced abundance of OsMS1^wenmin1^ in a temperature-dependent manner, it is plausible that the changes in the subcellular localization and abundance of OsMS1^wenmin1^ cause the decreased transcriptional activity. LXXLL motif was reported to mediate interactions between E proteins and p300/CBP histone acetyltransferases (HATs)^39^. Notably, the L301P substitution in OsMS1^wenmin1^ happens in the extremely conserved LXXLL motif (Fig. 1e and Supplementary Fig. 7). Considering that OsMS1 interacts with histones (Fig. 2a and Supplementary Fig. 9), we then conducted a mononuclease (MNase) digestion-qPCR assay to test whether OsMS1 could affect the chromatin status of the *EAT1* promoter. As shown in Supplementary Fig. 24a, b, we found that the *OsMS1*^*wenmin1*^ allele slightly influences the chromatin status of the *EAT1* promoter. It seems plausible that OsMS1 might interact with histone modifiers (e.g., histone acetyltransferases) to influence the chromatin status and gene expression. In addition, a previous study showed that OsMS1 interacts with TIP2/bHLH142 in Y2H and BiFC assays^23^, suggesting a possibility that OsMS1 may interact with multiple transcription factors to regulate gene expression.

Hengliangyouyihao bred from the *indica* HNS-1 with the *OsMS1*^*wenmin1*^ allele and MH63 was once the most widely planted two-line hybrid rice in 1990s in China (http://www.ricedata.cn/). However, the relatively higher male sterility-fertility transition temperature of HNS-1 restricted its further application^31^. Rice breeders prefer to use TGMS lines that had 22–24 °C transition temperature in practical two-line hybrid rice breeding^7,49,50^, because higher transition temperature usually causes self-pollination of TGMS lines during hybrid rice seed production under fluctuating environmental temperature conditions. We observed the transition temperatures of the *OsMS1*^*wenmin1*^ allele depend on genetic backgrounds. The *japonica* ZJ1-*wenmin1* confers temperature-sensitive genic male sterility with significantly decreased sterility-fertility transition temperature at ~23 °C (Fig. 1a), indicating that it has great potential as a better TGMS line for hybrid rice breeding. Considering that Leu^301^ of OsMS1 is evolutionarily conserved in plant species, it opens a way to create TGMS lines by generating the L301P mutation in various rice cultivars and other crops using genome editing technology.

## Methods

### Plant materials and growth conditions

The rice parents, transgenic materials, and NILs used in this study were listed in Supplementary Table 1. All rice plants, including Tian1S (*Oryza sativa* L. ssp. *indica*), ZhongJing1 (*Oryza sativa* L. ssp. *Japonica*, ZJ1), ZJ1-*wenmin1* (near-isogenic line of *OsMS1*^*wenmin1*^ in the ZJ1 background), HengnongS-1 (*Oryza sativa* L. ssp. *indica*, HNS-1), AnnongS-1 (*Oryza sativa* L. ssp. *indica*, AnS-1), Zhu1S (*Oryza sativa* L. ssp. *indica*), Y58S (*Oryza sativa* L. ssp. *indica*), C815S (*Oryza sativa* L. ssp. *indica*), Pei’ai64S (*Oryza sativa* L. ssp. *indica*, PA64S) and Nongken58S (*Oryza sativa* L. ssp. *Japonica*, NK58S), Pei’ai64 (*Oryza sativa* L. ssp. *indica*, PA64), Zhonghua11 (*Oryza sativa* L. ssp. *Japonica*, ZH11), Nipponbare (*Oryza sativa* L. ssp. *Japonica*, NIP), 93-11 (*Oryza sativa* L. ssp. *indica*), *gOsMS1*:ZJ1-*wenmin1* (genomic *OsMS1* transgene in ZJ1-*wenmin1* background), *35S:GFP-OsMS1* and *35S:GFP-OsMS1*^*wenmin1*^ (*OsMS1* and *OsMS1*^*wenmin1*^ overexpression in ZH11 background), and *35S: MYC-TDR* (*TDR* overexpression in ZH11 background) transgenic lines were grown in open fields with 16.7 × 20 cm spacing.

Rice plants were planted in the open field until their inflorescence length reached ~0.5 cm, an indicator of anther development stage 3^7^, and then transplanted to the growth chamber under the indicated day average temperature (DAT). Different anther development stages were valued by flower and anther length^51^. The photoperiod was 12 h light and 12 h dark with 75% relative humidity unless specifically indicated.

For transverse section analysis of OsMS1-GUS and OsMS1^wenmin1^-GUS in rice anther, *pOsMS1:OsMS1-GUS* and *pOsMS1:OsMS1*^*wenmin1*^*-GUS* transgenic plants were grown at 22 °C or 30 °C in growth chamber.

*Arabidopsis thaliana* ecotype Col-0 was used as wild-type control. *Arabidopsis* plants were grown on soil in a growth chamber at 22 °C ± 2 °C under long-day photoperiod (16 h light/8 h dark) with light intensity of 120 μmol photons m^−2^ s^−1^. To grow *Arabidopsis* seedlings on MS medium, seeds were surface-sterilized for 10 min in 15% commercial bleach, washed thoroughly with sterile water, and plated on half-strength MS medium with 1% sucrose and 0.8% agar. Plants were stratified at 4 °C for 2 days in the dark and then transferred to a growth chamber at the indicated times at 22 °C ± 2 °C under long-day photoperiod (16 h light/8 h dark) with light intensity of 120 μmol photons m^−2^ s^−1^.

*N. benthamiana* plants were grown on soil in a growth chamber at 22 °C ± 2 °C under long-day photoperiod (16 h light/8 h dark) with light intensity of 120 μmol photons m^−2 ^s^−1^. Five- to six-week-old *N. benthamiana* plants were used for transient expression assays.

### Yeast strains and growth conditions

*Saccharomyces cerevisiae* strain AH109 was cultured in yeast extract peptone dextrose (YPD) media at the indicated temperatures. The presence of transgenes was confirmed by growth on SD/-Trp/-Leu plates (Clontech, Cat. No: 630317). The transformed yeasts were suspended in 0.9% NaCl to OD_600_ = 1.0. Then, 3 μL of suspended yeast was spotted onto the indicated selection medium.

### Bacterial strains and growth conditions

The *Agrobacterium* strain GV3101 was incubated overnight and resuspended in the activation buffer (10 mM MES, pH 5.6, 10 mM MgCl_2_, and 150 µM acetosyringone). The desired *Agrobacterium* combinations were mixed and incubated at room temperature for at least 2 h by gentle rocking with the final concentration of each construct at OD_600_ = 1. The activated *Agrobacterium* strains were then infiltrated into fully expanded *N. benthamiana* leaves through a 1 mL needleless syringe.

*E. coli* BL21(DE3) transformed with the indicated constructs were grown to a density OD_600_ of 0.4–0.5 on LB supplemented with 100 µg/mL of ampicillin or 50 µg/mL Kanamycin. Protein expression was then induced by addition adding 0.1 mM IPTG and incubated 3 h at 37 °C with agitation.

### Generation of ZJ1-*wenmin1*

A NIL for *OsMS1*^*wenmin1*^ (ZJ1-*wenmin1*) was generated by backcrossing Tian1S with a wide compatibility *japonica* variety ZJ1 as the recurrent parent. The ZJ1-*wenmin1* was selected from the BC_6_F_2_ population based on pollen fertility and marker-assisted selection.

### Seed setting of Tian1S at permissive and restrictive temperatures

Tian1S and the wild-type control *indica* Pei’ai 64 (PA64) were planted in the open field in the Institute of Genetics and Developmental Biology, Chinese Academy of Sciences (40°22′N, 116°22′E) until their inflorescence length reached ~0.5 cm, and then transplanted to growth chamber for ~20 days at the indicated day average temperature (DAT) under photoperiod conditions of 12 h light and 12 h dark with 75% relative humidity. After full bolting, Tian1S and PA64 were replanted in the open field. Temperature profile set of the growth chamber was shown in Supplementary Table 3.

### Pollen fertility analysis

For pollen fertility analysis in Fig. 1a, 100 anthers of 20 mature spikelets from four plants in heading date were sampled just before their blooming. All anthers were taken out and stained with 1% potassium iodide-iodine solution (I_2_-KI). Pollen grains were released using a needle and debris was removed. More than 200 pollen grains were photographed in randomly picked microscopic fields under a Leica DM2500 microscope (Leica Microsystems) using a ×20 magnification objective to evaluate pollen fertility. Darkly stained and round pollen grains were defined as fertile, whereas unstained, lightly or incompletely stained, irregularly-shaped grains were defined as sterile. For detecting the pollen fertility of Tian1S at permissive temperature (22 °C) and restrictive temperature (30 °C) under photoperiod conditions of short day (10 h light and 14 h dark) and long day (14 h light and 10 h dark) in Supplementary Fig. 1b, temperature profile set of the growth chamber was shown in Supplementary Table 4. For examining pollen fertility in Fig. 1a, ZJ1, Tian1S, and ZJ1-*wenmin1* were planted at the indicated different day average temperature (DAT) under photoperiod conditions of 12 h light and 12 h dark in growth chamber. For statistical analysis of Fig. 1a in Fig. 1b, ten repeats of pollen fertility experiments were conducted for ZJ1, Tian1S, and ZJ1-*wenmin1*, each with ten anthers being examined. For examining pollen fertility in Fig. 1f, ZJ1, ZJ1-*wenmin1*, and *gOsMS1:*ZJ1-*wenmin1* lines (#1 and #2) were planted at natural high temperature (~30 °C, DAT).

### Semi-thin section

For transverse section analysis of anthers in Supplementary Fig. 2d, spikelets at different developmental stages were collected and embedded. Briefly, anthers were collected and fixed in FAA fixative solution containing a mixture of 37% formaldehyde, 70% ethanol, and 100% acetic acid (1:18:1). Samples were further dehydrated through an ethanol series of 70%, 85%, 95%, and 100% (v/v). After embedding in Technovit 7100 resin (Hereaus-Kulzer, Wehrheim, Germany), transverse sections of 2 μm were cut using a RM2265 automated microtome (Leica, Germany), and stained with 0.25% toluidine blue O (Chroma Gesellshaft Shaud). The tissue sections were then imaged under a light microscope (Olympus BH2, Japan).

### Map-based cloning of *wenmin1*

Tian1S is a thermosensitive male sterile *indica* variety. To identify the causal *wenmin1* gene, we conducted a map-based cloning by crossing Tian1S with a wide compatibility *japonica* variety ZJ1. The *wenmin1* was first delimited to a genomic interval between the SSR markers RM6235 and RM3600 on chromosome 9, and was then narrowed down to the markers RM24453 and A09-13, within a region covered by one BAC (AP005308). Three genes are located in this mapping region. Schematic structure of *LOC_Os09g27620* showed that *LOC_Os09g27620* has three exons and two introns. DNA sequencing showed that T902C mutation in *LOC_Os09g27620* is responsible for the temperature-sensitive male sterility phenotype of Tian1S and ZJ1-*wenmin1*.

Notably, the *OsMS1*^*wenmin1*^ mutation creates an *Hpa* II restriction site in the *OsMS1* coding sequence. To distinguish *OsMS1* and *OsMS1*^*wenmin1*^, genomic DNA was isolated from rice seedlings by CTAB method. The resulting DNA was used in a 20 μL PCR reaction with the primers A09-37F and A09-37R. The PCR products were digested with *Hpa* II and then separated using 2% agarose gel electrophoresis. The *Hpa* II digested PCR products of *OsMS1* and *OsMS1*^*wenmin1*^ were 168 bp and 87 bp, respectively.

### Allelism and functional complementation test

Hengnong S-1 (HNS-1), bred by the Hengyang Academy of Agricultural Sciences, was a widely used thermosensitive male sterile *indica* cultivar in the two-line hybrid breeding system in China (http://www.ricedata.cn/)^52^. To determine whether *OsMS1*^*wenmin1*^ in Tian1S was allelic to the causal mutation in HNS-1, we performed an allelic non-complementation assay in the F_1_ generation of a cross between Tian1S and HNS-1. To examine whether *OsMS1*^*wenmin1*^ is allelic to *tms5*, we crossed Tian1S with Annong S-1 and Zhu1S, both of which are *indica* and carry *tms5*^5^. We also crossed Tian1S with 93-11 as a control. As shown in Supplementary Table 5, All F_1_ heterozygous plants were grown in natural high-temperature conditions in open fields in Haikou (19°54′N, 110°33′E), Hainan Province when panicle development started in the summer. Male fertility was assayed as the seed setting rate, and plants with a seed setting rate lower than 10% were considered sterile.

For the functional complementation test, a fragment of 5.1 kb in length (designated *gOsMS1*), containing the entire *OsMS1*-coding region, along with 5′ and 3′ flanking regions was amplified from 93-11 genomic DNA using primers gOsMS1-F and gOsMS1-R, and cloned into the binary vector pMDC99. All constructs were sequencing-verified at TSINGKE Biological Technology (Beijing, China), and were individually transformed into *Agrobacterium tumefaciens* strain GV3101. *gOsMS1* was used to transform the *japonica* TGMS line ZJ1-*wenmin1*. We chose ZJ1-*wenmin1* for transformation because the *indica* variety Tian1S was difficult to transform. The presence of transgenes was determined by genomic PCR using oligonucleotide primers specific to the transgene constructs. We analyzed at least 10 independent lines.

### Protein alignment and structure analysis

The protein sequences used for alignment are encoded by LOC_Os09g27620, LOC_Os09g27620, XP_006660674.1, XP_021309975.1, GRMZM5G890224, XP_004956970.1, BAK05033.1 and XP_003578202.1 for Nip (Nipponbare), 93-11, Ob (Oryza brachyantha), Sb (Sorghum bicolor), Zm (Zea may), Si (Setaria italic), Hv (Hordeum vulgare), and Bd (Brachypodium distachyon), respectively. Alignments of protein sequences were performed using GeneDoc software (http://nrbsc.org/gfx/genedoc). The nuclear localization signal was predicted using NLStradamus with default parameters (http://www.moseslab.csb.utoronto.ca/NLStradamus/)^53^. The pre-loaded model was 2 state HMM dynamic, and the prediction cutoff value was 0.3. The plant homeodomain (PHD) was predicted using SMART (http://smart.embl-heidelberg.de/smart/set_mode.cgi?NORMAL=1)^54^.

### Phylogenetic analysis of OsMS1 and its homologs

The protein sequence of OsMS1 was used to identify its homologs via BLAST search. A condensed phylogenetic tree was constructed with MEGA software (version 5.2)^55^ using the Neighbor-Joining (NJ) method with the following parameters: Poisson model, Uniform rates, Complete deletion, and 1000-replicates Bootstrap. The homolog proteins are named according to species and their names or NCBI accession numbers. Bootstrap values (>50) are shown as percentages for each branch. Branch lengths are shown proportional to the amino acid variation rates. The genebank number for homolog proteins in *Manihot esculenta, Jatropha curcas, Ricinus communis, Populus trichocarpa, Eucalyptus grandis, Medicago truncatula, Glycine max, Vigna angularis, Citrus clementina, Cucumis sativus, Brassica oleracea, Raphanus sativus, Camelina sativa, Arabidopsis lyrata, Solanum tuberosum, Nicotiana attenuata, Elaeis guineensis, Phoenix dactylifera, Sorghum bicolor, Setaria italica, Oryza brachyantha, Brachypodium distachyon*, and *Aegilops tauschii* are OAY25374.1, KDP38178.1, XP_002521912.1, XP_002315838.1, XP_010043916.1, XP_003613773.1, XP_003518728.1, XP_017427127.1, XP_006448066.1, XP_004140423.1, XP_013610019.1, XP_018444384.1, XP_010493299.1, XP_020876299.1, XP_006355027.1, XP_019242345.1, XP_010917298.2, XP_017701237.1, XP_021309975.1, XP_004956970.1, XP_006660674.1, XP_003578202.1, and EMT13329.1, respectively.

### Gene expression pattern analysis

Tissue-specific expression patterns of *OsMS1* and *TDR* were obtained from Botany Array Resource (http://bar.utoronto.ca/efprice/cgi-bin/efpWeb.cgi)^56^. The signal threshold was set to 1000 for all data extraction. The expression specificity of *OsMS1* and *TDR* were also obtained from Rice Expression Profile Database (RiceXPro) (https://ricexpro.dna.affrc.go.jp/).

### The quantitative real-time PCR analysis

Plant materials were ground into fine powders in liquid nitrogen and total RNA was isolated by RNAprep pure kit (TIANGEN, Cat. No: DP439-H) according to the manufacturer’s instructions. Total RNAs were treated with RNase-free DNase I (Sigma-Aldrich, Cat. No: 04716728001) according to the manufacturer’s protocol. Full-length cDNAs were synthesized using the FastQuant RT Super Mix kit (TIANGEN, Cat. No: KR108). qPCR reactions were performed on a Lightcycler 480 machine (Roche Applied Science, USA) using 2×SYBR Green supermix kit (BIO-RAD, Hercules, CA). To eliminate DNA contamination, qPCR-specific amplification was verified by a single band product in gel analysis. Data were calculated with the comparative cycle threshold (CT) method and normalized to the housekeeping gene *OsACTIN1* in all qPCR experiments unless specifically indicated. Primers are summarized in Supplementary Data 1. For detecting *OsMS1* and *OsMS1*^*wenmin1*^ mRNA levels in Fig. 3g. Means of the *LUC/RLUC* mRNA ratios were normalized to *OsMS1-LUC/RLUC* at 22 °C.

### Plasmid construction and plant transformation

All constructs used in this study were generated using an EZfusion kit (Genera, Cat. No: GR6086). To generate the plasmid *35S:GFP-OsMS1*, the full-length open reading frame (ORF) of *OsMS1* was amplified with the primers GFP-OsMS1-F and GFP-OsMS1-R from young panicles of *indica* variety 93-11 and cloned into pMDC43 vector. To generate the plasmid *35S:GFP-OsMS1*^*wenmin1*^, the ORF of *OsMS1*^*wenmin1*^ was amplified with the primers GFP-OsMS1-F and GFP-OsMS1-R from young panicles of Tian1S and cloned into the pMDC43 vector. To generate the plasmid *35S:MYC-TDR*, the ORF of *TDR* was amplified with the primers MYC-TDR-F and MYC-TDR-R from young panicles of ZH11 and cloned into the pCAMBIA1300-221-MYC vector. To generate the plasmid *gOsMS1-GUS*, the genomic fragment of *OsMS1* was amplified with the primers gOsMS1-F and gOsMS1-R from young panicles of 93-11 and cloned into the pMDC164 vector. To generate the plasmid *pOsMS1:OsMS1-GUS* and *pOsMS1:OsMS1*^*wenmin1*^*-GUS*, the full-length ORF of *OsMS1* and *OsMS1*^*wenmin1*^ was amplified and fused in frame with *pOsMS1* and then cloned into the pMDC164 vector. *35S:GFP-OsMS1*, *35S:GFP-OsMS1*^*wenmin1*^*, 35S:MYC-TDR, pOsMS1:OsMS1-GUS*, and *pOsMS1:OsMS1*^*wenmin1*^*-GUS* were introduced into ZH11 via Agrobacterium-mediated transformation^57^. *35S:GFP-OsMS1* and *35S:GFP-OsMS1*^*wenmin1*^ were also introduced into wild-type *Arabidopsis* Col-0 via Agrobacterium-mediated transformation using standard protocol^58^. We analyzed at least 10 independent lines for each construct. Primers are summarized in Supplementary Data 1.

### Peptide synthesis

Biotin-H2A (1-21), Biotin-H2B (1-21), Biotin-H3 (1-21), Biotin-H4 (1-21), Biotin-H4K5ac, Biotin-H4K8ac, Biotin-H4K12ac, and Biotin-H4K16ac peptides used in this study were biochemically synthesized by Bankpeptide Bio-Tech Co., Ltd (Hefei, China) with a purity >95%. Biotin-H3K4me3 (Cat. No: 81042), Biotin-H3K9ac (Cat. No: 81044), and Biotin-H3K14ac (Cat. No: 81001) were obtained from Active Motif. All peptides were dissolved in sterile pure water. Sequences of all peptides are summarized in Supplementary Table 6.

### Histone pull-down assay

To generate the plasmid *FLAG-OsMS1*, the ORF of *OsMS1* was amplified with the primers FLAG-OsMS1-F and FLAG-OsMS1-R and cloned into the pET-nT vector. To generate the plasmid *FLAG-OsMS1*^*wenmin1*^, the ORF of *OsMS1*^*wenmin1*^ was amplified with the primers FLAG-OsMS1-F and FLAG-OsMS1-R and cloned into the pET-nT vector. To generate the plasmid *GST-OsMS1C*, the ORF of *OsMS1C* was amplified with the primers GST-OsMS1C-F and GST-OsMS1C-R and cloned into the pGEX4T-1 vector. To generate the plasmid *FLAG-OsMS1C* with the mutations *C642A, C645A, H650A, C653A*, the synthesized mutated *OsMS1C* was amplified with the primers FLAG-OsMS1C-F and FLAG-OsMS1C-R and cloned into the pET-nT vector. To generate the plasmid *FLAG-GST*, the ORF of *GST* was amplified with the primers FLAG-GST-F and FLAG-GST-R and cloned into the pET-nT vector. To generate the plasmid *FLAG-GFP*, the ORF of *GFP* was amplified with the primers FLAG-GFP-F and FLAG-GFP-R and cloned into the pET-nT vector. The peptide pull-down assays were performed in a three hundred microliters system^59^. Briefly, in vitro purified FLAG-OsMS1, FLAG-OsMS1^wenmin1^, GST-OsMS1C (residues 626–683), FLAG-OsMS1C with the mutations C642A, C645A, H650A, C653A, FLAG-GFP, or FLAG-GST were stored in 50 mM Tris-HCl pH 7.4, 200 mM NaCl at −80 °C. Equal amounts (~5 μg) of purified FLAG-OsMS1, GST-OsMS1C, or FLAG-GST proteins in peptide-binding buffer (50 mM Tris-HCl 7.4, 200 mM NaCl, 0.05% NP-40) were precleaned with Streptavidin agarose beads (Sigma-Aldrich, Cat. No: 17-5113-01) to prevent nonspecific binding and then aliquoted equally into several parts. 1 μg of biotinylated histone peptides with different modifications was added to the individually aliquoted mixture and then incubated at 4 °C for half an hour with gentle rotation. Pull-down experiments were performed by adding 10 μL prewashed Streptavidin beads (Sigma-Aldrich, Cat. No: 17-5113-01) and incubated with rotation at 4 °C for half an hour. After washing with 3× 1 mL binding buffer for 1 min at 4 °C, the bound proteins were detected by immunoblotting using antibody to FLAG (Abmart, Cat. No: M20008, dilution 1:5000) or GST (Abmart, Cat. No: M20007, dilution 1:5000). Primers are summarized in Supplementary Data 1. For in vitro histone pull-down assay in Fig. 2a, the indicated proteins were pulled down by strepavidin beads and detected by western blots with antibody against FLAG (Abmart, Cat. No: M20008, dilution 1:5000) or GST (Abmart, Cat. No: M20007, dilution 1:5000). Empty streptavidin beads were used as negative binding control.

### *Semi-*in vivo glutathione *S*-transferase pull-down assay

To generate the plasmid *GST-OsMS1*, the ORF of *OsMS1* was amplified with the primers GST-OsMS1-F and GST-OsMS1-R and cloned into the pGEX4T-1 vector. Primers are summarized in Supplementary Data 1. To examine whether OsMS1 interacts with H4 in Fig. 2d, we conducted a *semi-*in vivo glutathione S-transferase (GST) pull-down assay using nuclear proteins of stage 9 rice anthers. In brief, purified GST-OsMS1 was incubated with nuclear proteins extracts from stage 9 anthers of rice young panicles. Proteins were pulled down by Glutathione Sepharose 4B (GE Healthcare, Cat. No: 45-000-139) and detected by western blots using anti-H4 antibody (Active motif, Cat. No: 61521, Clone: MABI 0400, dilution 1:5000) or anti-GST antibody (Abmart, Cat. No: M20007, dilution 1:5000). GST was used as a negative control.

### Co-immunoprecipitation assay

To confirm the interaction between OsMS1 and H4 in Fig. 2e, stage 9 anthers of rice young panicles of *35S:OsMS1* transgenic rice was immunoprecipitated with GFP-Trap^®^_A agarose beads (Chromotek, Cat. No: gta-20), then endogenous histone H4 was detected using antibody against H4 (Active Motif, Cat. No: 61521, Clone: MABI 0400, dilution, 1:5000).

Homozygous transgenic plants expressing *35S*:*GFP* and *35S*:*GFP*-*OsMS1* were crossed with transgenic plants expressing *35S*:*MYC*-*TDR*, the double transgene plants of *35S:GFP*/*35S*:*MYC*-*TDR* and *35S*:*GFP*-*OsMS1*/*35S*:*MYC*-*TDR* were determined by PCR, and used to confirm the interaction between OsMS1 and TDR. Briefly, about 0.5 g young panicles with stage 9 anthers were grinded into powder in liquid nitrogen. Notably, all the subsequent steps were carried out in a 4 °C cold room or on ice. Briefly, about two volumes of protein extraction buffer (50 mM Tris-HCl, pH 7.5, 150 mM NaCl, 1% Triton X-100, 5% glycerol, 1 mM EDTA, 1× Roche protease inhibitor cocktail) were added to each sample to homogenize the powder. The resuspended samples were centrifuged at 13,523 × *g* for 10 min and the supernatant was subsequently transferred to new 1.5 mL tubes. GFP-Trap^®^_A agarose (Chromotek, Cat. No: gta-20) was then added to the tubes and incubated at 4 °C for 30 min with gentle rocking. The beads were collected by centrifugation and washed at least three times with the wash buffer (50 mM Tris-HCl, pH 7.5, 150 mM NaCl, 0.2% Triton X-100, 5% glycerol, 1 mM EDTA, 1× Roche protease inhibitor cocktail). Finally, the bound proteins were eluted by heating the beads in a 1×SDS loading buffer at 95 °C for 5 min. The eluted proteins were separated in a 10% (w/v) SDS-polyacrylamide gel, and probed with an anti-GFP antibody (Abmart, Cat. No: M20004, dilution, 1:5000), an anti-MYC antibody (Abmart, Cat. No: M20002, dilution, 1:5000) or anti-H4 antibody (Active Motif, Cat. No: 61521, Clone: MABI 0400, dilution, 1:5000).

### Transcription activation assay

Transcription activation assay was performed using Matchmaker GAL4 Two-Hybrid System 3 (Clontech, USA) following the manufacturer’s instructions. The full-length and a series of truncations or mutations of OsMS1 were amplified and fused with the DNA-binding domain (BD) in vector pGBKT7 (Clontech, USA). All the resulting constructs were sequencing-verified and cotransformed with pGADT7 (Clontech, USA) into *Saccharomyces cerevisiae* strain AH109. The presence of transgenes was confirmed by growth on SD/-Trp/-Leu plates (Clontech, Cat. No: 630317). The transformed yeasts were suspended in 0.9% NaCl at OD_600_ = 1.0. Then, 3 μL of suspended yeast was spread into SD/-Trp/-Leu/-His (Clontech, Cat. No: 630319) + 2 mM 3-aminotriazole (Sigma-Aldrich, Cat. No: A8056) or SD/-Trp/-Leu/-His/-Ade (Clontech, Cat. No: 630323) plates. Yeast growth was observed after incubation at the indicated temperatures. For detecting the transcriptional activation activity in Fig. 3a, yeast cells were spotted on SD-2 or SD-3 plus 2 mM 3-aminotriazole (3-AT) plates and grown at low (22 °C) or high (30 °C) temperatures for 4d. For quantifying the transactivation activities in Fig. 3a, we conducted yeast liquid assays. Equal numbers of diploid yeast cells were cultured in the SD-2 or SD-3 liquid medium at 22 °C (OD_600_ = 0.05) and 30 °C (OD_600_ = 0.01) for the indicated time. Relative yeast growth was shown as (OD_600_ in SD-3)/(OD_600_ in SD-2).

### Yeast two-hybrid assay

Yeast two-hybrids were performed using Matchmaker GAL4 Two-Hybrid System 3 following the manufacturer’s instructions. As the full-length OsMS1 autoactivated the reporter gene when fused with BD, we used the N-terminal fragment of OsMS1 (referred to as OsMS1_1-625_, amino acids 1-625) as bait to screen a cDNA library derived from rice inflorescence.

To check for the interaction between OsMS1_1-625_, OsMS1^wenmin1^, OsMS1^L304L305AA^, and TDR, the coding sequence of OsMS1^wenmin1^, OsMS1^L304L305AA^, and OsMS1_1-625_ were fused with the BD in pGBKT7 (Clontech, Cat. No: 630443), and the coding sequence of TDR was fused with the DNA-activation domain (AD) in pGADT7 (Clontech, Cat. No: 630442). Constructs for testing interactions were cotransformed into yeast strain AH109. The presence of transgenes was confirmed by growth on SD/-Trp/-Leu plates (Clontech, Cat. No: 630317). The transformed yeasts were suspended in 0.9% NaCl to OD = 1.0. Then, 3 μL of suspended yeast was spotted onto a stringent selection medium SD/-Trp/-Leu/-His/-Ade (Clontech, Cat. No: 630323). Interactions were observed after incubation at the indicated temperatures. For detecting the temperature-dependent association of OsMS1^wenmin1^ and TDR in Fig. 4a, yeast cells (OD_600_ = 1) were spotted on the SD-4 plates and grown at 22 °C, 26 °C, and 30 °C for 96 h.

To quantify the temperature-dependent association of OsMS1^wenmin1^ and OsMS1^L304L305AA^ with TDR in yeast in Supplementary Fig. 18a, equal amounts of yeast diploid cells were cultured in 10 mL liquid SD-4 (Clontech, Cat. No: 630322) medium at 22°C, 26°C, and 30°C for about 96 h until yeast cells coexpressing OsMS1_1-625_-BD and TDR-AD were grown to an OD_600_ of ~2.0. Yeast growth were shown as OD_600_ and normalized to yeast cells coexpressing OsMS1_1-625_-BD and TDR-AD.

### Luciferase complementation imaging assay

For confirming OsMS1-interacting proteins, coding sequences of *OsMS1* were cloned into vector pCAMBIA1300-35S-nLUC-RBS^60^ using primers OsMS1-Nluc-F and OsMS1-Nluc-R. The full-length ORF of TDR was cloned into the vector pCAMBIA1300-35S-cLUC-RBS using primers Cluc-TDR-F and Cluc-TDR-R. All the constructs were introduced into *Agrobacterium tumefaciens* strain GV3101. Primers are summarized in Supplementary Data 1. The LCI assay was carried out in *N. benthamiana* leaves^61,62^. In brief, *Agrobacterium* strains GV3101 with individual expression constructs were incubated overnight and resuspended in the activation buffer (10 mM MES, pH 5.6, 10 mM MgCl_2_ and 150 µM acetosyringone). The desired *Agrobacterium* combinations were mixed and incubated at room temperature for at least 2 h by gentle rocking with the final concentration of each construct at OD_600_ = 1. The activated *Agrobacterium* strains were then infiltrated into fully expanded *N. benthamiana* leaves through a 1 mL needleless syringe. After infiltration, plants were incubated at 22 °C for 44 h and then transferred at the indicated temperatures for 4 h until LUC activity was measured. For examining the interactions in Fig. 2b, the indicated Nluc and Cluc construct combinations were transiently expressed in *N. benthamiana* at 22 °C for 2 d. For examining the temperature-dependent associations of OsMS1 and OsMS1^wenmin1^ with TDR in LCI assays in Fig. 4b, *N. benthamiana* plants infiltrated with the *Agrobacteria* combinations were incubated at 22 °C for 44 h and then transferred to 30 °C or not for another 4 h.

To detect luciferase activity, 1 mM D-luciferin solution (Promega, Cat. No: E1602) was sprayed onto the leaves of *N. benthamiana*, followed by incubation in darkness for 5–10 min. A low-light cooled CCD imaging apparatus (NightOWL II LB983 with Indigo software) was used to capture the image. An exposure time of 5 min was used to take images.

To quantify protein–protein interaction intensity, leaf discs infiltrated with the desired *Agrobacterium* combinations were taken and incubated with 50 μL water containing 1 mM D-luciferin in a Costar^®^ 96-Well White Flat Bottom Polystyrene High Bind Microplate (Corning, Cat. No: 7400) for 5–10 min. The luminescence activity was measured using the GLOMAX 96 microplate luminometer (Promega, USA). The protein–protein interaction intensity was shown as relative luminescence unit (RLU). To avoid the bias of transfection efficiency, we used at least twelve individual leaves of six tobacco plants for each combination.

### Luciferase assay

For detecting the temperature-dependent protein abundance, *35S:OsMS1* or *35S:OsMS1*^*wenmin1*^ were cloned into vector pGreenII 0800-LUC^62^ using primers pGreenII 0800-OsMS1-LUC-F and pGreenII 0800-OsMS1-LUC-R. *35S:TDR* were cloned into vector pGreenII 0800-LUC^62^ using primers pGreenII 0800-TDR-LUC-F and pGreenII 0800-TDR-LUC-R, and used as a control. Primers are summarized in Supplementary Data 1. All the constructs were introduced into *A. tumefaciens* strain GV3101.

The *Agrobacterium* bacteria were activated and then infiltrated into *N. benthamiana* plants as LCI assay. After infiltration, plants were incubated at 22 °C for 44 h and then transferred to the indicated temperatures. The firefly and Renilla luciferase activity were detected using the Dual-Luciferase® Reporter Assay System (Promega, Cat. No: E1960) in the GLOMAX 96 microplate luminometer (Promega, USA) following the manufacturer’s instructions. The firefly luciferase activity was normalized to the Renilla luciferase activity to control for transfection efficiency. The relative firefly luciferase activity reflects the protein abundance of OsMS1, OsMS1^wenmin1^, or TDR at the indicated temperatures. Three independent replicates were performed for each condition.

### Confocal microscopy observation

For the subcellular localization of OsMS1 in rice, root tip cells of 7-day-old *35S:GFP-OsMS1* transgenic rice grown at 22 °C were visualized using a laser scanning confocal microscope (Zeiss LSM710 META, Germany). Nuclei were stained blue by 4′,6-diamidino-2-phenylindole (DAPI).

For the subcellular localization of OsMS1 and OsMS1^wenmin1^ in *Arabidopsis*, root tip cells and cotyledons of 4-day-old *35S:GFP-OsMS1* and *35S:GFP-OsMS1*^*wenmin1*^ transgenic *Arabidopsis* plants were visualized using a laser scanning confocal microscope (Nikon confocal microscope A1R, Japan).

For the subcellular localization of OsMS1 and OsMS1^wenmin1^ in *N. benthamiana*, *35S:GFP-OsMS1* + *35S:mCherry* or *35S:GFP-OsMS1*^*wenmin1*^ + *35S:mCherry* were transiently co-expressed in fully expanded leaves of 6-week-old *N. benthamiana* as described in LCI assay. The GFP and RFP fluorescence signals were visualized using a laser scanning confocal microscope (Zeiss LSM710 META, Germany) after 48–72 h incubation at 22 °C in the dark.

For the subcellular localization of OsMS1 and OsMS1^wenmin1^ in rice protoplast, we used the *japonica* variety ZH11 to prepare and transform rice protoplasts. The plasmids (10 μg per construct) were introduced by PEG-mediated transfection^63^. The GFP and DAPI signals were visualized using a laser scanning confocal microscope (Zeiss LSM710 META, Germany) after 18 h incubation at 28 °C in the dark.

To examine whether GFP-OsMS1 and GFP-OsMS1^wenmin1^ protein levels are temperature-dependent in Fig. 3c, *N. benthamiana* expressing *35S:GFP-OsMS1* or *35S:GFP-OsMS1*^*wenmin1*^ were grown at 22 °C for 44 h then transferred to 30 °C or not for another 4 h. The GFP signal in leaves was visualized under a laser scanning confocal microscope (Zeiss LSM710 META, Germany). The excitation wavelength used for DAPI, GFP/YFP, and mCherry is 405 nm, 488 nm, and 543 nm, respectively. For quantitative analysis of Fig. 3c in Fig. 3d, the GFP-OsMS1 and GFP-OsMS1^wenmin1^ protein levels were quantified by comparing the GFP fluorescence intensity of the cell expressing *35S:GFP:OsMS1* or *35S:GFP:OsMS1*^*wenmin1*^ with that of RFP fluorescence intensity of 35S:mCherry in the same cell. Fluorescence intensity was determined using ImageJ software. All the images in a single experiment were captured under the same setting parameters. Zen Black 2009 (Zeiss) was used to export pictures taken by Zeiss LSM710 META.

### Bimolecular fluorescence complementation assays

To generate the plasmid *35S:OsMS1-cYFP* and *35S:OsMS1*^*wenmin1*^*-cYFP* the ORF of *OsMS1* and *OsMS1*^*wenmin1*^ was amplified with the primers OsMS1-YC-F and OsMS1-YC-R and cloned into the *pSPYCE(M)* vector^64^. To generate the plasmid *35S:H4-nYFP*, the ORF of *H4* was amplified with the primers H4-YN-F and H4-YN-R and cloned into the *pSPYNE173* vector^64^. To generate the plasmid *35S:TDR-nYFP*, the ORF of *TDR* was amplified with the primers TDR-YN-F and TDR-YN-R and cloned into the *pSPYNE173* vector^64^. Primers are summarized in Supplementary Data 1. Co-transfection of the indicated constructs (for example, those encoding OsMS1-cYFP and TDR-nYFP) into *N. benthamiana* leaf epidermal cells was performed as LCI assay. YFP fluorescence was visualized and photographed using a confocal scanning microscope (Zeiss LSM710 META, Germany). The excitation wavelength used for YFP and mCherry is 488 nm and 543 nm, respectively. Each BiFC assay was repeated at least three times. For detecting the interactions of OsMS1 with H4 in Fig. 2c, the indicated nYFP and cYFP construct combinations were transiently expressed in *N. benthamiana* at 22 °C for 48 h. For detecting the temperature-dependent interactions of OsMS1 and OsMS1^wenmin1^ with TDR in Fig. 4d, the co-infiltrated tobacco plants were incubated at 22 °C for 44 h and then transferred to 30 °C for another 4 h or not.

### In vitro pull-down assay

To produce GST-tagged OsMS1 and OsMS1^wenmin1^ proteins, the full-length coding sequences of *OsMS1* and *OsMS1*^*wenmin1*^ were amplified and cloned into the GST vector pGEX-4T-1. To produce FLAG-tagged TDR protein (referred to as FLAG-TDR), the coding sequence of *TDR* was amplified and cloned into the vector pET-nT. Primers are summarized in Supplementary Data 1.

To examine protein–protein interactions in Fig. 4e, recombinant proteins were expressed in *E. coli* BL21 (DE3). Equal amounts (~2 μg) of purified GST, GST-OsMS1 or GST-OsMS1^wenmin1^ proteins were incubated with ~2 μg of recombinant FLAG-TDR in a volume of 600 μL in buffer (50 mM Tris-HCl, pH 7.5, 150 mM NaCl, 1.5 mM MgCl_2_, 1 mM EGTA, pH 8.0, 1% Triton X-100, 10% glycerol, 1×Complete Protease Inhibitor cocktail, and 1 mM phenylmethylsulfonyl fluoride). 30 μL Glutathione Sepharose 4B (GE Healthcare, USA) was then added to each combined solution with gentle rotation at 4 °C for 30 min. The beads were collected by centrifugation at 23 × *g* for 1 min and washed at least four times with the wash buffer (50 mM HEPES, pH 7.5, 150 mM NaCl, 1.5 mM MgCl_2_, 1 mM EGTA, pH 8.0, 1% Triton X-100, 10% glycerol, 1×Complete Protease Inhibitor cocktail, and 1 mM phenylmethylsulfonyl fluoride). Finally, the bound proteins were eluted by heating the beads in a 1×SDS loading buffer at 95 °C for 5 min. The eluted proteins were separated by a 10% SDS-PAGE and detected by immunoblotting using an anti-GST antibody (Abmart, Cat. No: M20007, dilution, 1:5000), and an anti-FLAG antibody (Abmart, Cat. No: M20008, dilution, 1:5000), respectively. Signals were detected using eECL Western Blot Kit (Cwbiotech, Cat. No: CW0049), and images were scanned using Tanon-4500 (Shanghai, China).

### Rice protoplast preparation

We planted ZH11 in growth chamber for two weeks. Approximately 50–200 leaves at the three-leaf stage were treated with macerozyme and cellulase to prepare protoplast. Briefly, the leaves were cutted into ~0.5 mm fragments, and then incubated in an enzyme solution (0.75% Macerozyme R-10, 1.5% Cellulose RS, 0.6 M mannitol, 10 mM MES at pH 5.7, 10 mM CaCl_2_, and 0.1% BSA) for 4–5 h in the dark with gentle rotation. After digestion, the same volume of W5 solution (154 mM NaCl, 5 mM KCl, 125 mM CaCl_2_, and 2 mM MES at pH 5.7) was added, and followed by vigorous shaking for 10–30 s. Protoplasts were collected by filtering through 40 μm nylon mesh with three to five washes using W5 solution. The pellets were collected by centrifugation at 150 × *g* for 5 min. After washing twice with W5 solution, the pellets were then resuspended in MMG solution (0.4 M mannitol, 4 mM MES at pH 5.7, and 15 mM MgCl_2_) at a concentration of 2× 10^6^ cells mL^−1^ for subcellular localization or 2 × 10^7^ cells mL^−1^ for ChIP-qPCR. Protoplasts were further transiently transformed with the indicated constructs by PEG-mediated transfection according to a published protocol^63^. Briefly, the indicated DNA plasmids (10 μg) were mixed together with 300 μL protoplasts and the freshly prepared 330 μL polyethylene glycol (PEG) solution (0.4 M mannitol, 40% w/v PEG4000, 0.1 M CaCl_2_), and then incubated at room temperature for 20 min. After serial dilutions with W5 solution, the transfected protoplasts were resuspended in W5 solution and cultured overnight at 28 °C in the dark.

### Western blot analysis

About 0.5 g stage 9 anthers of *pOsMS1:OsMS1-GUS* and *pOsMS1:OsMS1*^*wenmin1*^*-GUS* transgenic plants were used to extract OsMS1 or OsMS1^wenmin1^ protein in Figs. 3i, 3j and Supplementary Fig. 14b. Proteins were prepared as co-immunoprecipitation (Co-IP) assay in Fig. 2e, and detected by anti-GUS antibody (Abmart, Cat. No: P26299, dilution, 1:2000) and anti-ACTIN antibody (Abmart, Cat. No: M20009, dilution, 1:5000).

To examine the subcellular localization of OsMS1 and OsMS1^wenmin1^ in Supplementary Fig. 10f, nuclear and cytoplasmic fractions were prepared from stage 9 anthers of *pOsMS1:OsMS1-FLAG* and *pOsMS1:OsMS1*^*wenmin1*^*-FLAG* transgenic rice. About 0.5 g anthers were ground into powder in liquid nitrogen and homogenized in 5 mL Honda buffer (20 mM HEPES-KOH at pH 7.4, 1.25% ficoll, 2.5% Dextran T40, 0.44 M sucrose, 10 mM MgCl_2_, 0.5% (v/v) Triton X-100, 5 mM DTT, 1 mM PMSF, Roche protease inhibitor cocktail), and then filtered through 62 μm nylon mesh. Triton X-100 was added to a final concentration of 0.5%, and the mixture was incubated on ice for 15 min. The solution was centrifuged at 1500 × *g* for 10 min, and the supernatant was used to detect the cytoplasmic protein. The remaining pellets were washed with Honda buffer containing 0.1% (v/v) Triton X-100 and then used to extract the nucleus protein. Proteins were detected by western blots with antibody against FLAG (Abmart, Cat. No: M20008, dilution, 1:5000), H4 (Active Motif, Cat. No: 61521, dilution, 1:5000) or BIP (Abcam, Cat. No: ab108615, dilution, 1:5000). H4 and BIP were used as a marker for nuclei and cytoplasm, respectively.

### GUS histochemical staining

Transgenic rice plants were planted in the open field until their inflorescence length reached ~0.5 cm, an indicator of anther development stage 3^7^, and then transferred to the growth chamber under the indicated day average temperature (DAT). For GUS-staining, panicles were submerged in buffer containing 50 mM NaPO_4_ buffer, 0.4 mM each K_3_Fe(CN)_6_/K_4_Fe(CN)_6_, 0.1% (v/v) Triton X-100, and 1 mM X-Gluc and stained at 37 °C until blue color became visible. Then the samples were incubated with 75% ethanol until chlorophyll was removed. The GUS-staining panicles were taken photos using a microscope. Notably, *pOsMS1:OsMS1-GUS* and *pOsMS1:OsMS1*^*wenmin1*^*-GUS* panicles were treated under the same setting parameters. For detecting endogenous OsMS1-GUS proteins in Fig. 1d, *pOsMS1:OsMS1-GUS* transgenic plants were grown at planted in the open field and then transplanted to 22 °C DAT in growth chamber. For detecting endogenous OsMS1-GUS or OsMS1^wenmin1^-GUS proteins in Fig. 3h and Supplementary Fig. 14a, *pOsMS1:OsMS1-GUS* and *pOsMS1:OsMS1*^*wenmin1*^*-GUS* transgenic plants were grown in the open field and then transplanted to 22 °C or 30 °C DAT in growth chamber.

### Electrophoretic mobility shift assay

To generate the plasmid *MBP-OsMS1* and *MBP-OsMS1*^*wenmin1*^, the ORF of *OsMS1* and *OsMS1*^*wenmin1*^ was amplified with the primers MBP-OsMS1-F and MBP-OsMS1-R and cloned into the pMAL-c2 vector. To generate the plasmid *MBP-TDR*, the ORF of *TDR* was amplified with the primers MBP-TDR-F and MBP-TDR-R and cloned into the pMAL-c2 vector. Primers are summarized in Supplementary Data 1. MBP-OsMS1 and MBP-TDR proteins were expressed and purified as described in in vitro pull-down assay. The oligonucleotide sequences containing E-box sequences in the rice *EAT1* promoter were found by manual search in the promoter sequence. Double-stranded biotin-labeled DNA probes were generated by annealing sense and antisense oligonucleotides. Annealing reactions were performed at 95 °C for 10 min with 0.5 °C decrease per minute until 25 °C in a PCR thermocycler.

EMSA was conducted using Chemiluminescent Nucleic Acid Detection Module Kit (Thermo Scientific). EMSA reactions were performed in a 20 μL system containing 10 mM Tris-Hcl pH 7.5, 2.5% glycerol, 1 mM DTT, 50 mM KCl, 5 mM MgCl_2_, 50 ng/μL Poly (dI·dC), and 0.05% NP-40. The binding assays of the indicated proteins to the *EAT1* promoter were performed by incubating 10 fmol Biotin-dsDNA probe with 1 μg MBP-TDR or MBP-OsMS1 proteins (1 μL) at room temperature for 30 min. To test whether TDR binds to the *EAT1* promoter differently with OsMS1 or OsMS1^wenmin1^, biotin-labeled probes were incubated with a constant amount of MBP-TDR protein with increasing amounts of MBP-OsMS1, MBP-OsMS1^wenmin1^ or MBP. Protein-DNA complexes were resolved on native 9% (w/v) polyacrylamide gels at 0.5 × TBE at 100 V for 90 min at 4 °C. Electrophoresed protein-DNA complexes were transferred to a Hybond N^+^ nylon membrane (Amersham, GE Healthcare Life Sciences) at 100 V for 60 min using 0.5 × TBE at 4 °C. The transferred protein-DNA complexes were further cross-linked to the membrane for 10 min with the membrane facing a transilluminator equipped with 312 nm bulbs. Detection of Biotin-labeled DNA by chemiluminescence was performed following the instructions of the manufacturer.

### Chromatin immunoprecipitation and quantitative real-time PCR analysis

The proteins expressed in protoplasts were used for the ChIP assay. In brief, we used ZH11 to prepare rice protoplasts as described in rice protoplast preparation section^63^. The indicated plasmids with final concentration at 5 μg per 100 μL were introduced by PEG-mediated transfection. ChIP experiments were performed according to the published protocol^65^. Briefly, approximately 10 mL protoplasts were cross-linked with 1% (v/v) formaldehyde in ice for 10 min. The crosslink was stopped with the addition of 0.125 M glycine for 5 min. After shearing chromatin into about 500 bp fragments via sonication, anti-MYC antibody (Abcom, Cat. No: ab32, dilution, 1:200) was used to immunoprecipitate genomic DNA fragments. Immunoprecipitated DNA was extracted with phenol/chloroform and precipitated with ethanol. The ChIP experiments were quantified by qPCR in triplicate with appropriate primers (Supplementary Data 1). Empty MYC vector was used as the control. The relative enrichment of promoter DNA was calculated by the % input method with qPCR analysis.

In Fig. 5f, young panicles of *pOsMS1:OsMS1-FLAG* and *pOsMS1:OsMS1*^*wenmin1*^*-FLAG* transgenic plants grown at 22 °C or 30 °C in growth chamber were used. Approximately 2 g young panicles with stage 9 anthers of the transgenic lines were ground into powder in liquid nitrogen and cross-linked using extraction buffer with 1% (v/v) formaldehyde in ice for 10 min. The crosslink was stopped with the addition of 0.125 M glycine for 5 min. The chromatin was isolated and the immunoprecipitation assay was conducted as described above. Anti-FLAG antibody (Abcom, Cat. No: ab 205606, dilution, 1:200) was used to immunoprecipitate genomic DNA fragments. The ChIP experiments were quantified by qPCR in triplicate with appropriate primers (Supplementary Data 1). The relative enrichment of promoter DNA was calculated by the % input method with qPCR analysis.

### Open chromatin detection by MNase digestion

Open chromatin was detected using a published protocol^66^. In brief, ~0.5 g young panicles with stage 9 anthers of ZJ1 and ZJ1-*wenmin1* were ground into power in liquid nitrogen and suspended in 10 mL of cold nuclei isolation buffer (1 M hexylene glycol, 20 mM PIPES-KOH at pH 7.6, 10 mM MgCl_2_, 1 mM EGTA, 15 mM NaCl, 0.5 mM spermidine, 0.15 mM spermine, 0.5% Triton X-100, 10 mM β-mercaptoethanol and 1× Roche protease inhibitor cocktail) with gentle rotation for 15 min. The suspension was filtered through 30 nm CellTrics, and the elute was centrifuged for 10 min at 1500 × *g* at 4 °C. The pellets were resuspended as crude nuclei extracts with digestion buffer (40 mM Tris-HCl at pH 7.9, 0.3 M Suc, 10 mM MgSO_4_, 1 mM CaCl_2_, and 1× Roche protease inhibitor cocktail). Equal aliquot of crude nuclei extracts was subject to digestion with increasing MNase amounts at 30 °C for 20 min and DNA was extracted using phenol:chloroform treatment followed by ethanol precipitation and used for qPCR analysis.

### Statistics and reproducibility

All data are shown as the mean ± s.e.m. unless indicated otherwise. Statistical analysis was performed using GraphPad Prism 7 software (GraphPad Software, Inc. San Diego, CA, USA). All details on statistics have been indicated in figure legends. For all quantitative measurements, a normal distribution was assumed. For comparisons between two groups, the two-tailed unpaired Student’s *t* test was applied. No method was undertaken to calculate statistical power and corresponding sample size. Instead, sample size was determined empirically on the basis of experience with similar assays and from sample sizes generally used by other investigators^5,61^. Images were processed and analyzed with Adobe Photoshop and ImageJ.

### Reporting summary

Further information on research design is available in the Nature Research Reporting Summary linked to this article.

## Supplementary information

Supplementary Information

Description of Additional Supplementary Files

Supplementary Data 1

Reporting Summary

## Data Availability

The authors declare that all data supporting the findings of this study are available within the article and its Supplementary Information files. Sequence details for rice *OsMS1*, *TDR*, and *EAT1* genes used in this study can be found in China Rice Data Center (https://www.ricedata.cn/gene/) with the following accession numbers: LOC_Os09g27620, LOC_Os02g02820, and LOC_Os04g51070, respectively. Tissue-specific expression patterns were obtained from Botany Array Resource (http://bar.utoronto.ca/efprice/cgi-bin/efpWeb.cgi). Expression profiles were derived from RiceXPro (https://ricexpro.dna.affrc.go.jp/). The primers used in this study are provided as Supplementary Data 1. Source data are provided with this paper.

## Source data

Source data
