# Supplementary material for: A natural allele of OsMS1 responds to temperature changes and confers thermosensitive genic male sterility
Source: Nat Commun. 2022 Apr 19;13:2055. doi: 10.1038/s41467-022-29648-z (PMC9018702; doi:10.1038/s41467-022-29648-z)
Supplement: Supplementary file 1 — Supplementary Information [file 41467_2022_29648_MOESM1_ESM.pdf]

A natural allele of *OsMS1* responds to temperature changes and confers thermosensitive genic male sterility

Wu *et al.*

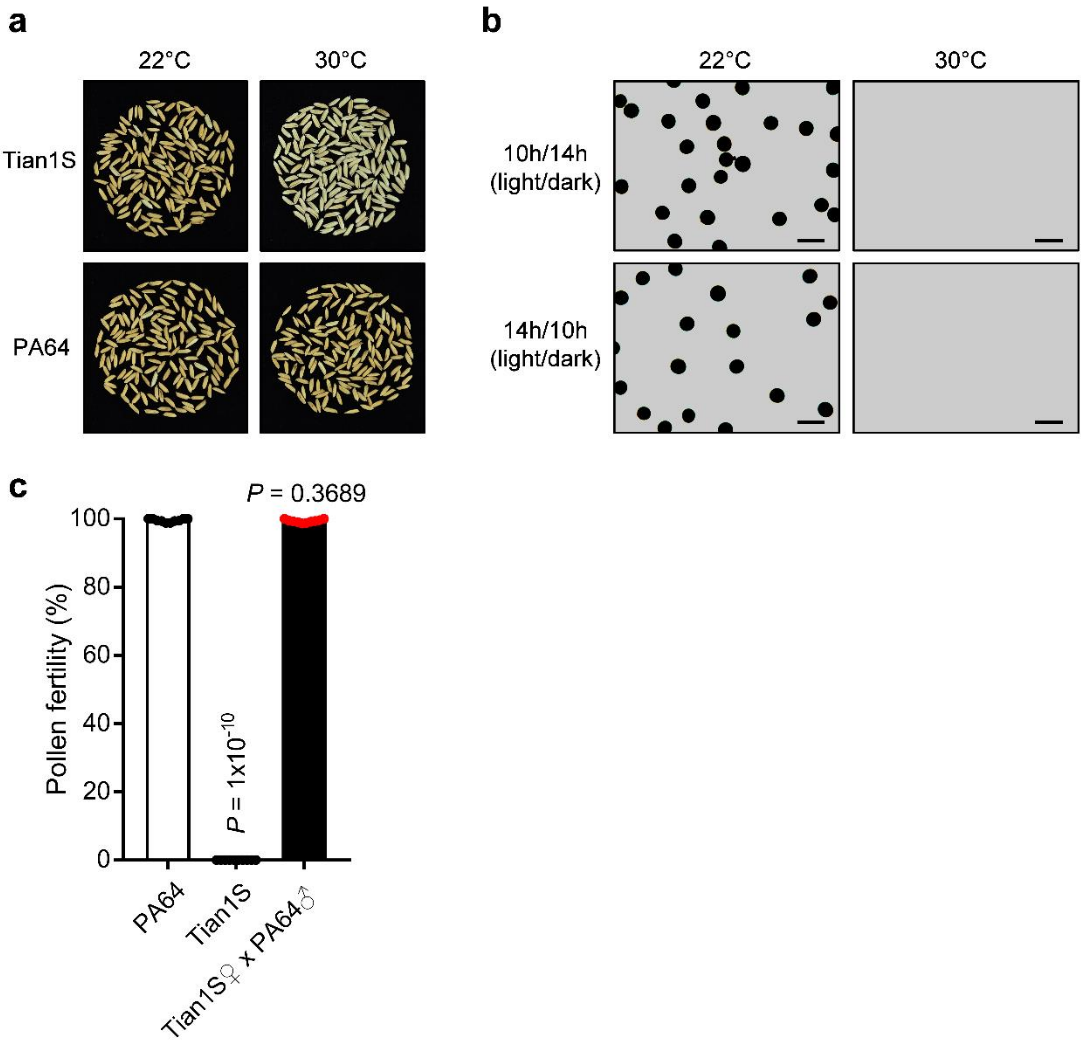

Supplementary Fig. 1. Characterization of a thermosensitive male sterile mutant Tian1S.

a, Seed setting of Tian1S at permissive and restrictive temperatures. Tian1S was sterile with no seed setting, compared with the wild type control *indica* Pei'Ai 64 (PA64) under restrictive temperature (30°C). **b,** Pollen fertility of Tian1S at permissive temperature (22°C) and restrictive temperature (30°C) under photoperiod conditions of short day (10 h light and 14 h dark) and long day (14 h light and 10 h dark). **c,** Pollen fertility of PA64, Tian1S and a cross of Tian1S with PA64. PA64, Tian1S and the heterozygous F_1 plants were grown at 30°C in growth chamber. Ten repeats of pollen fertility experiments were conducted, each with ten anthers being examined. Values are shown as the mean ± s.e.m. (n =10 biological repeat). Source data are provided as a Source Data file.

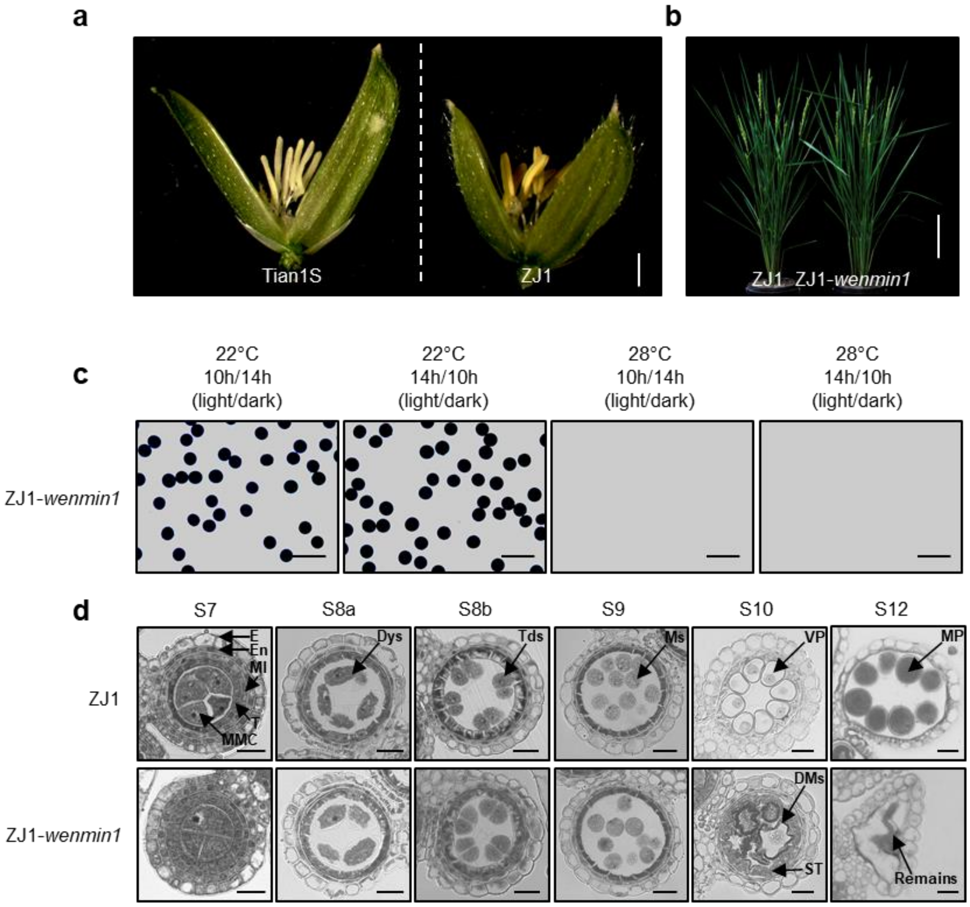

Supplementary Fig. 2. The *wenmin1* allele confers the thermosensitive male-sterile phenotype in the *japonica* variety ZJ1.

a, Spikelets of Tian1S (left) and ZJ1 (right). Plants were grown at high temperature in the open fields of the Institute of Genetics and Developmental Biology Chinese Academy of Sciences (40°22'N,116°22'E, Beijing). **b,** Whole plant morphology of ZJ1 (left) and ZJ1-*wenmin1* (right) at the heading stage. Plants were grown at natural high temperature in the open field (19°54'N, 110°33'E, Haikou) when panicle development started in the summer. **c,** Pollen fertility of ZJ1-*wenmin1* at low temperature (22°C) and high temperature (28°C) under photoperiod conditions of short day (10 h light and 14 h dark) and long day (14 h light and 10 h dark). **d,** Semi-thin section analysis of ZJ1 and ZJ1-*wenmin1* anthers grown under high temperature (~30°C) at MMC (S7), dyad (S8a), tetrad (S8b), Early microspore (S9), vacuolated microspore (S10), and mature pollen (S12) stages. The images are cross sections of a single locule. E, epidermis; En, endothecium; ML, middle layer; T, tapetum; MMC, microspore mother cell; Dys, dyads; Tds, tetrads; Ms, microspores; Vp, Vacuolated pollen; DMs, degenerated microspores; MP, mature pollen; ST, swollen tapetal layer, Remains, only cell debris of both tapetal cells and pollen grains remained. Scale bars, 100 μm [(**a**) and (**c**)], 15 cm (**b**), 20 μm (**d**). Representative results of at least three independent experiments in **d** are shown. Source data are provided as a Source Data file.

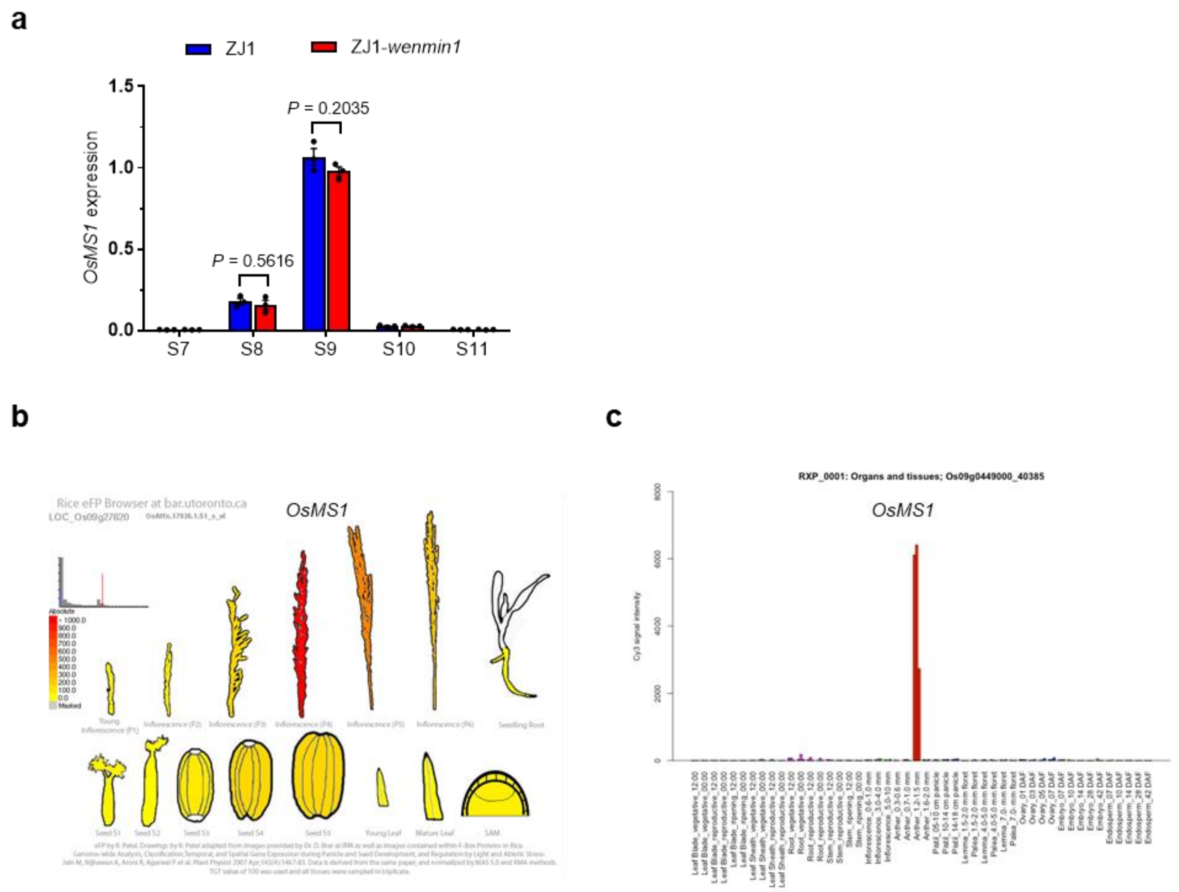

Supplementary Fig. 3. Expression patterns of *OsMS1/LOC_Os09g27620*.

a, Expression profiles of *OsMS1* determined by qPCR analysis. Expression levels were normalized against *OsACTIN1*. Values are means ± s.e.m. (n = 3 biological replicates). Two-tailed unpaired t-test was used for statistical analysis. S7, S8, S9, S10, S11 represent stage 7, stage 8, stage 9, stage10, stage 11 during rice anther development, respectively. **b,** Tissue-specific expression patterns of *OsMS1* obtained from Botany Array Resource (http://bar.utoronto.ca/efprice/cgi-bin/efpWeb.cgi). Signal threshold was set to 1,000 for all the data extraction. **c,** Expression profiles of *OsMS1* derived from RiceXPro (https://ricexpro.dna.affrc.go.jp/). Source data are provided as a Source Data file.

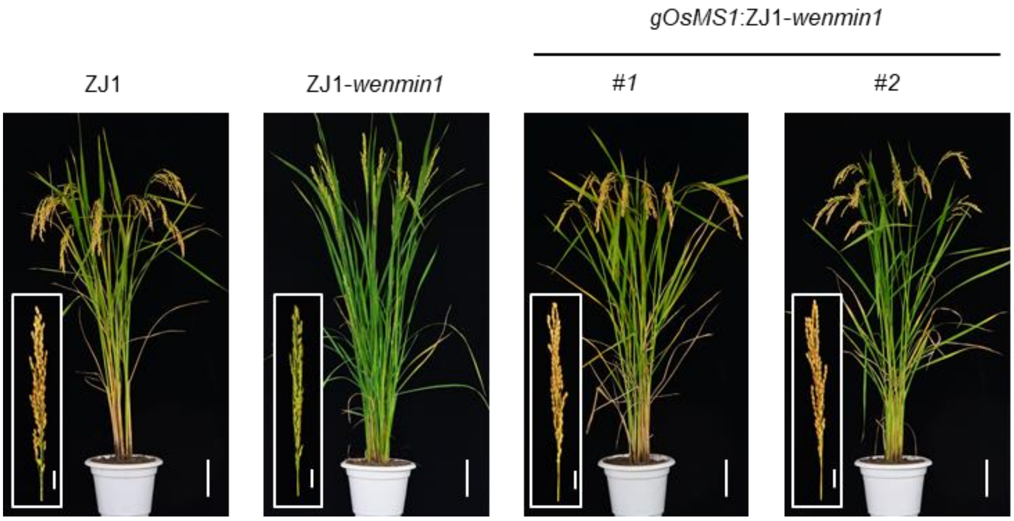

Supplementary Fig. 4. *wenmin1* is a natural allele of *LOC_Os09g27620.*
The genomic fragment of *OsMS1* complements the thermosensitive male sterile phenotype of ZJ1-*wenmin1* at restrictive temperature. Whole plant and panicle (inset) phenotypes of ZJ1, ZJ1-*wenmin1* mutant and *gOsMS1:*ZJ1-*wenmin1* lines (#1 and #2). Plants were grown under natural high temperature in the open field (19°54'N, 110°33'E, Haikou). Scale bars, 15 cm (main panel); 2 cm (inset).

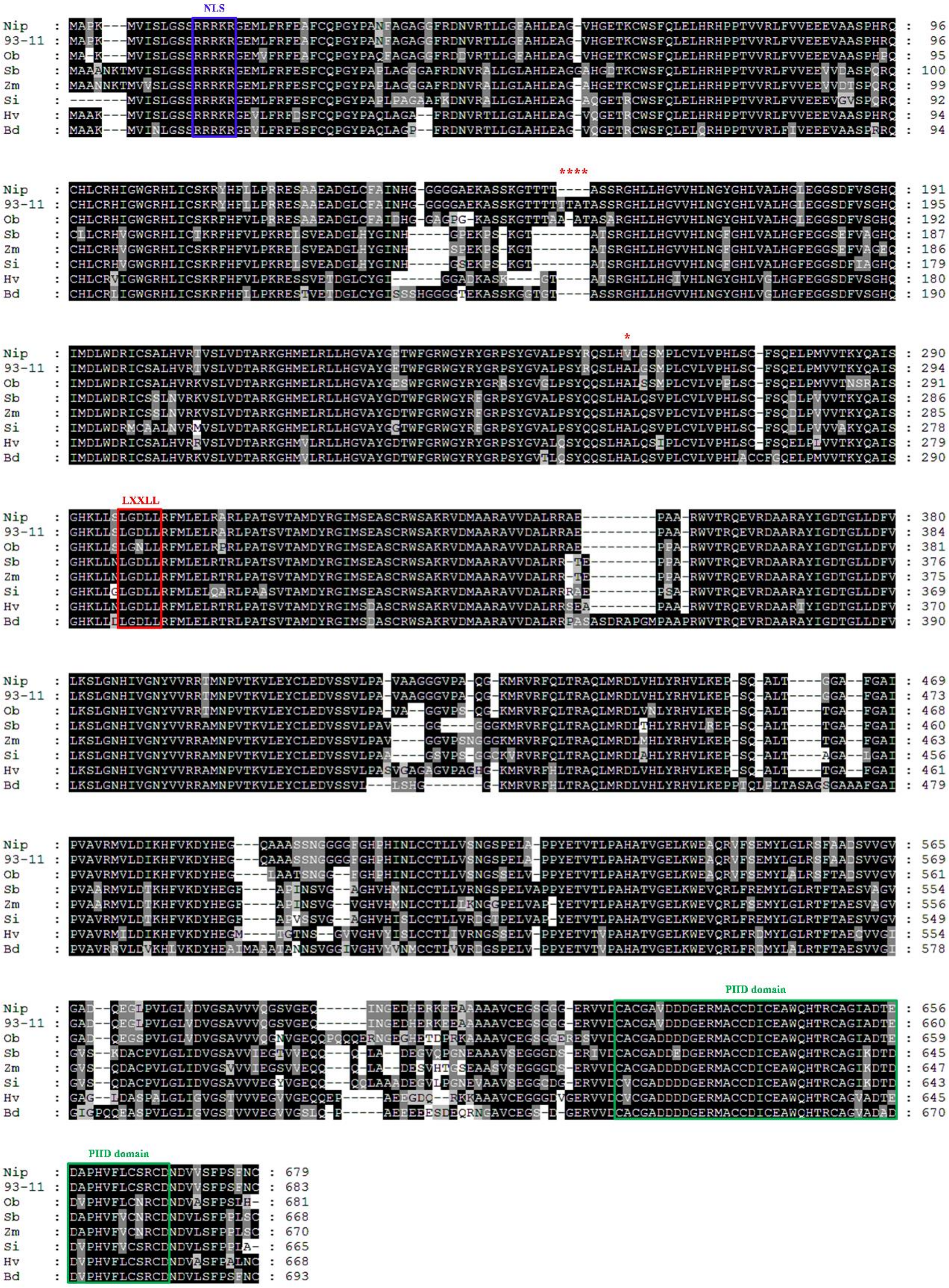

Supplementary Fig. 5. Alignments of the deduced amino acid sequence of OsMS1 with its homologs.

The predicted nuclear localization signal (NLS) is shown in blue box. The conserved LXXLL motif is shown in red box. The conserved plant homeodomain (PHD domain) is shown in green box. Red asterisks indicate the amino acid differences in OsMS1 between *indica* and *japonica*. Identical residues are shaded in black, whereas

conserved residues are shaded in gray. The protein sequences used for alignment are encoded by *LOC_Os09g27620*, *LOC_Os09g27620*, *XP_006660674.1*, *XP_021309975.1*, *GRMZM5G890224*, *XP_004956970.1*, *BAK05033.1* and *XP_003578202.1* for Nip (Nipponbare), 93-11, Ob (*Oryza brachyantha*), Sb (*Sorghum bicolor*), Zm (*Zea may*), Si (*Setaria italic*), Hv (*Hordeum vulgare*) and Bd (*Brachypodium distachyon*), respectively.

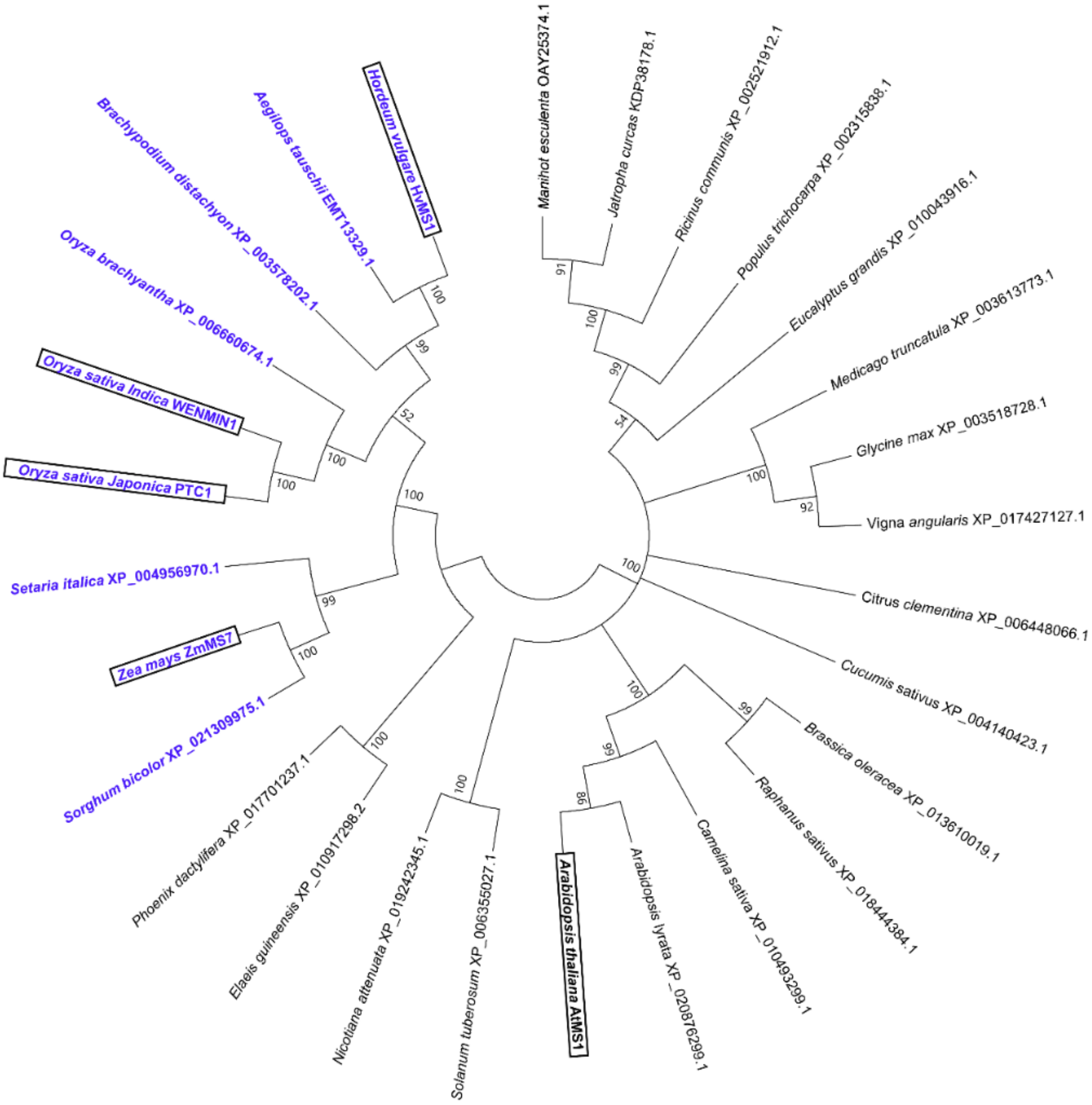

Supplementary Fig. 6. Phylogenetic analysis of OsMS1 and its homologs.
The protein sequence of OsMS1 was used to identify its homologs via BLAST search. A condensed phylogenetic tree was constructed with MEGA software (version 5.2) using the Neighbour-Joining (NJ) method with the following parameters: Poisson model, Uniform rates, Complete deletion, 1,000-replicates Bootstrap. The homolog proteins are named according to species and their names or NCBI accession numbers. Bootstrap values (>50) are shown as percentages on each branch. Branch lengths are shown proportional to the amino acid variation rates. Blue branches show the most similar members of OsMS1 from different species. Notably, the loss function of *OsPTC1*, *HvMS1*, *ZmMS7* and *AtMS1* in *Oryza sativa Japonica*, *Hordeum vulgare*, *Zea mays* and *Arabidopsis thaliana* are male sterile. OsPTC1, HvMS1, ZmMS7 and AtMS1 are highlighted by the black rectangle.

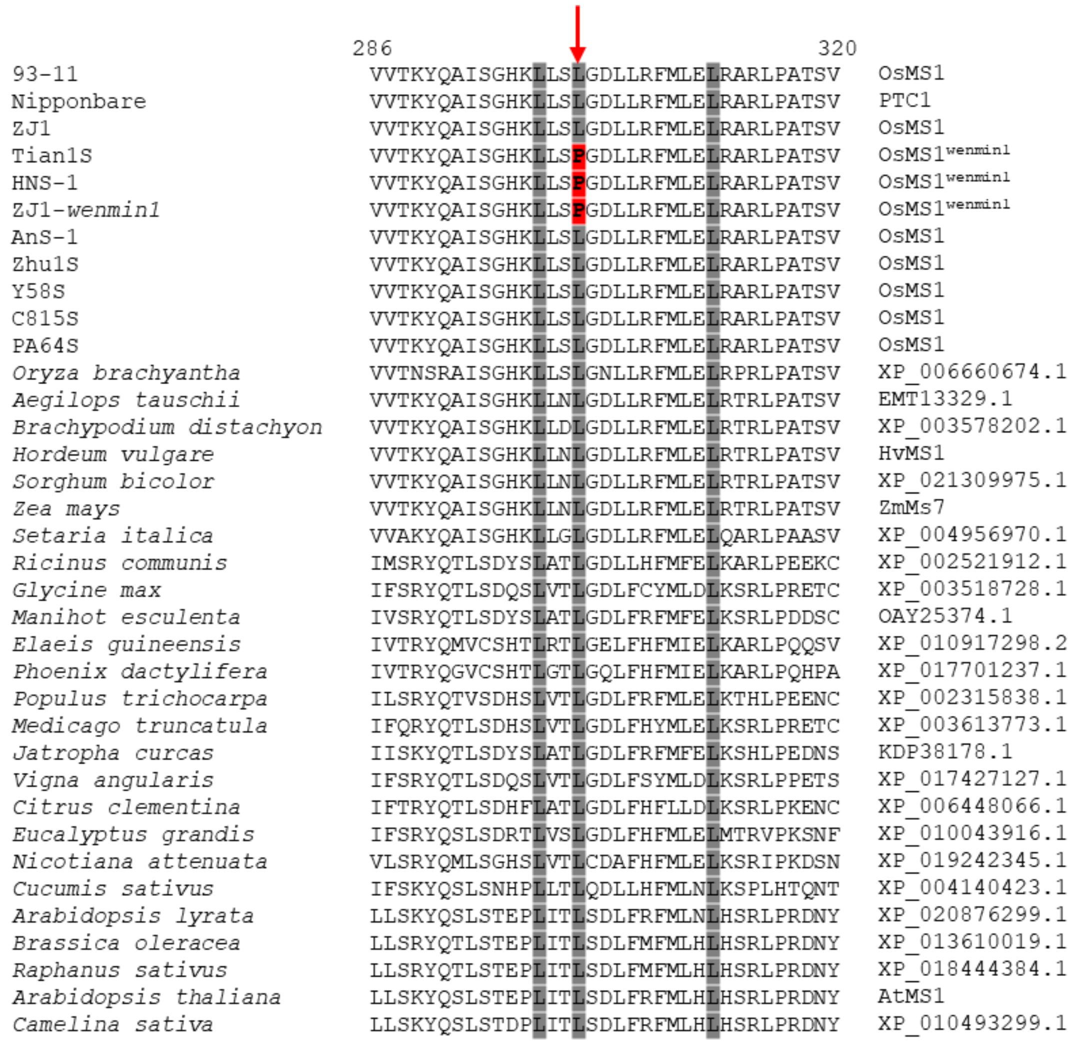

Supplementary Fig. 7. The OsMS1 amino acid residue that is mutated in Tian1S is highly conserved (red arrow; L, Leucine).

A multiple sequence alignment of OsMS1 protein sequences surrounding the point mutation site with its homologs of the indicated organisms. Arrowhead indicates L301 in OsMS1 and the equivalent residues in its homologs. The mutated amino acids (L301P) in HNS-1, Tian1S, ZJ1-*wenmin1* are shaded in red. Three highly conserved leucines (L) are shaded in gray. The numbering refers to OsMS1 amino acid position. Left, names of the indicated organisms. Middle, alignment of OsMS1 protein sequences surrounding the point mutation site with its homologs. Right, proteins names or NCBI accession numbers.

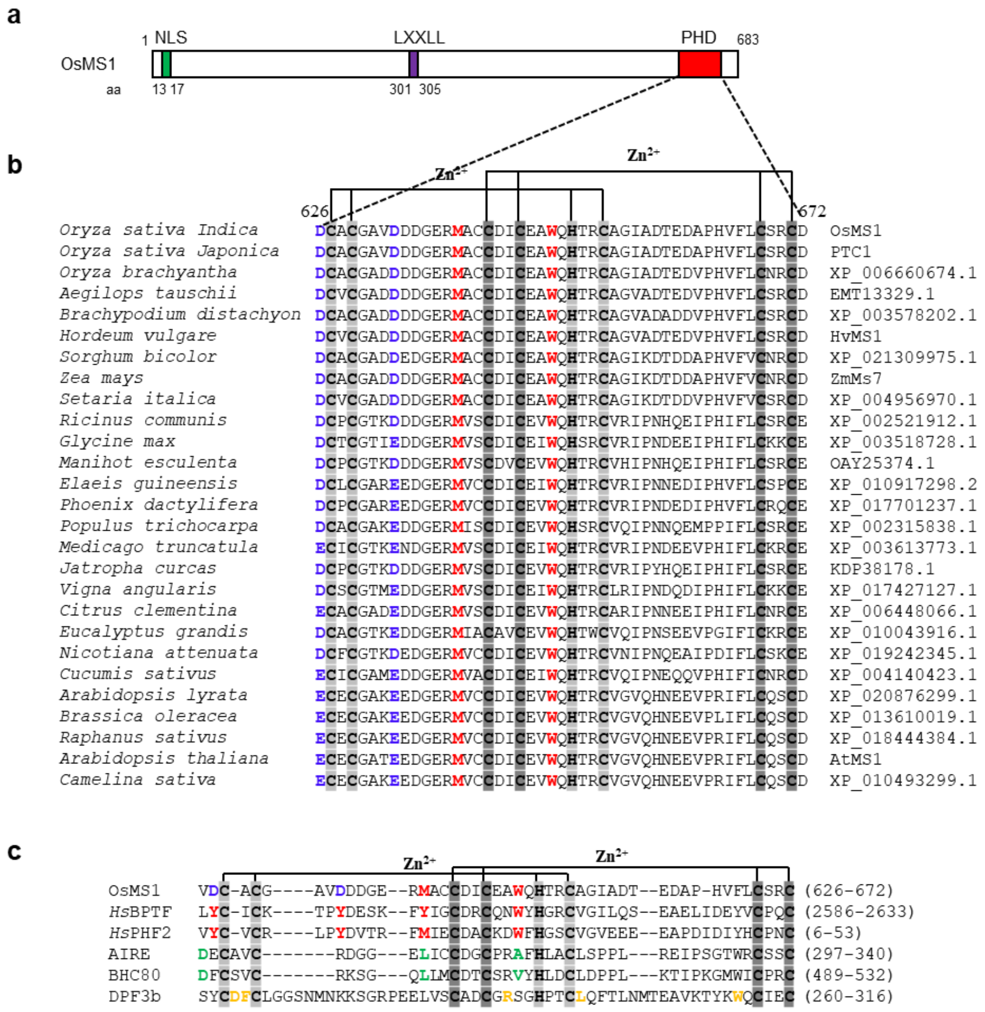

Supplementary Fig. 8. Sequence alignments of the PHD domain of OsMS1.

a, Schematic representation of OsMS1 protein structure. Nuclear localization signal (NLS, green rectangle), Leu-X-X-Leu-Leu motif (LXXLL, purple rectangle) and plant homeodomain (PHD, red rectangle). Numbers indicate the amino acid position (aa). **b,** Multiple sequence alignments of the PHD domain of OsMS1 with its orthologs of the indicated organisms. Residues correspond to the highly conserved methionine and tryptophan in the aromatic cage for H3K4me3 recognition are colored red; acidic amino acids correspond to the aromatic cage are colored blue. The numbering above the sequence refers to OsMS1 amino acid position. **c,** Sequence alignment of OsMS1 PHD domains with the representative PHD finger sequences. Residues important for H3K4me3 recognition in *Hs*BPTF and *Hs*PHF2, H3K4me0 recognition in AIRE and BHC80, H3K14ac recognition in DPF3b are colored red, green and orange, respectively. Acidic amino acids in OsMS1 correspond to the aromatic cage are colored blue. The conserved Zinc-coordinating residues are shown in light gray for Zinc 1 and dark gray for Zinc 2 (**b** and **c**). The residue numbers corresponding to the PHD finger in the full-length protein are shown in right. *Homo species* (*Hs*).

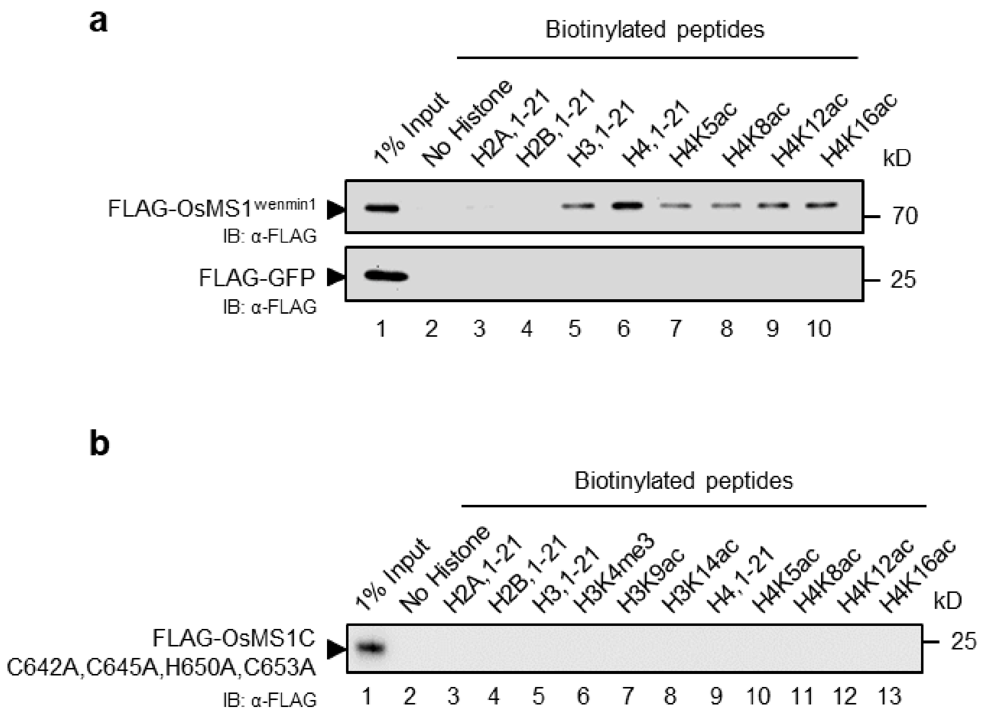

Supplementary Fig. 9. PHD domain but not L301 of OsMS1 is crucial for binding histones.

a, In vitro histone pull-down assay with FLAG-OsMS1^{wenmin1} against the indicated biotinylated peptides. Proteins were pulled down by strepavidin beads and detected by western blots with antibody against FLAG. **b**, The mutations (C642A, C645A, H650A and C653A) within the PHD domain abolished the interactions between OsMS1 and histone peptides. Proteins were pulled down by strepavidin beads and detected by western blots with antibody against FLAG. Representative results of at least three independent experiments are shown. Source data are provided as a Source Data file.

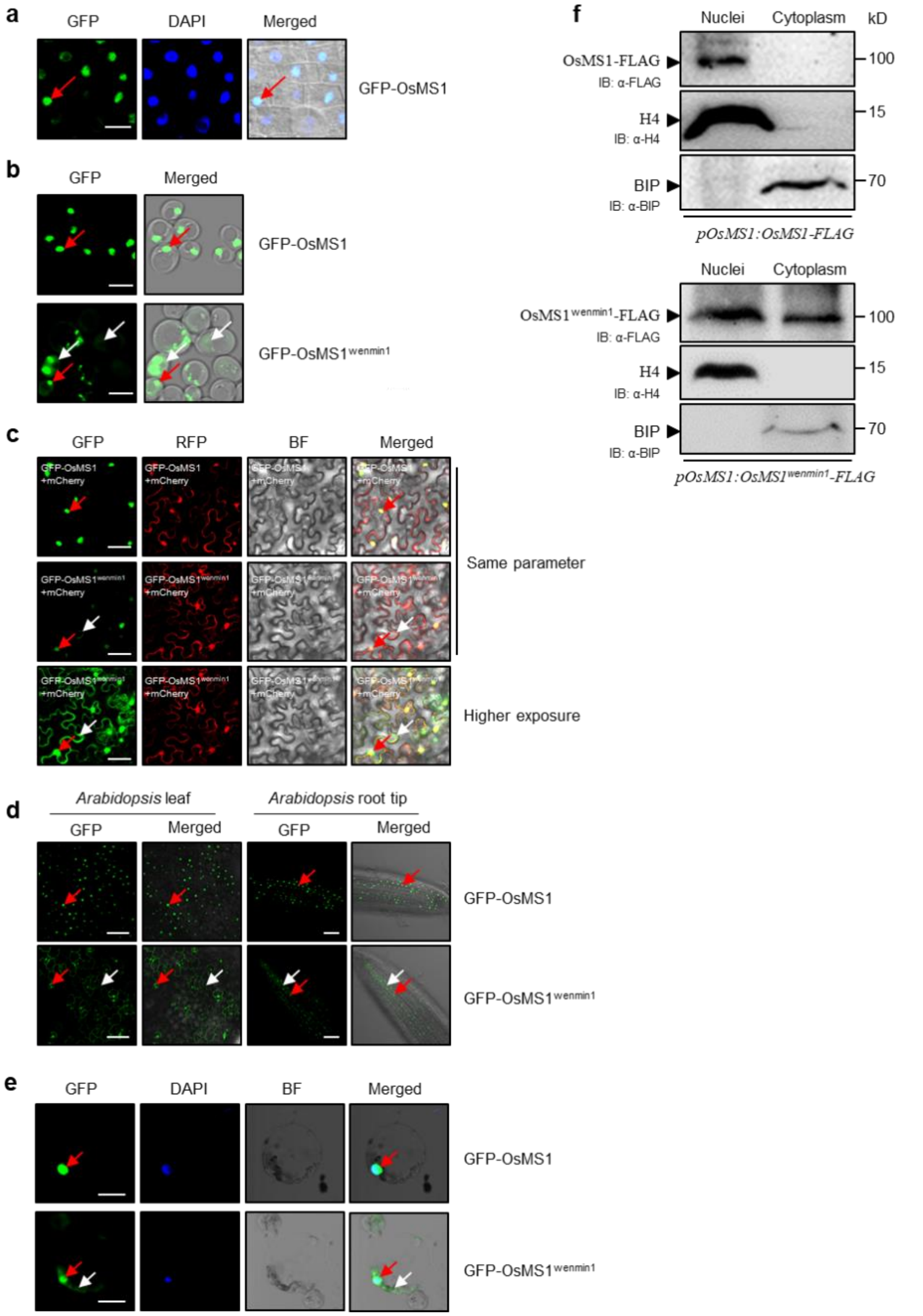

Supplementary Fig. 10. L301 is important for nucleus localization of OsMS1.

a, Subcellular localization of GFP-OsMS1 in *35S:GFP-OsMS1* rice root tip cells. GFP-OsMS1 green fluorescence was observed in the nucleus (left), overlapped with nuclei (middle, stained blue with 4′-6-Diamidino-2-phenylindole, DAPI). Scale bars, 10 μm. **b,** Subcellular localization of GFP-OsMS1 and GFP-OsMS1^{wenmin1} in yeast.

Full-length OsMS1 and OsMS1^{wenmin1} were fused in frame with BD and cotransfected with AD. Yeast cells were grown in SD-2 at 22°C until OD_{600}=1.0. BD, GAL4-DNA binding domain; AD, GAL4-activation domain; SD-2, plates lacking Trp and Leu. Scale bars, 5 μm. **c,** Subcellular localization of GFP-OsMS1 and GFP-OsMS1^{wenmin1} in *N. benthamiana* plants. *N. benthamiana* plants infiltrated with the indicated *Agrobacteria* were incubated at 22°C for 48 h, then GFP signals were visualized under confocal microscopy. Scale bars, 50 μm. **d,** Subcellular localization of GFP-OsMS1 and GFP-OsMS1^{wenmin1} in *Arabidopsis*. GFP signals of 4-day-old transgenic *Arabidopsis* expressing *35S:GFP-OsMS1* and *35S:GFP-OsMS1*^{wenmin1} in *Arabidopsis* leaf (left) and root tips (right) were visualized under confocal microscopy. GFP, GFP channel; Merged, Merged images from GFP and bright-field image. Scale bar, 50 μm. **e**, Subcellular localization of GFP-OsMS1 and GFP-OsMS1^{wenmin1} in rice protoplast. GFP signals of rice protoplast expressing *35S:GFP-OsMS1* and *35S:GFP-OsMS1*^{wenmin1} were visualized under confocal microscopy. GFP, GFP channel; DAPI, 4′-6-Diamidino-2-phenylindole; BF, bright field; Merged, Merged images from GFP and bright field image. Scale bar, 20 μm. **f,** Subcellular localization of OsMS1 and OsMS1^{wenmin1} in transgenic rice. Nuclei and cytoplasm were extracted from stage 9 anthers of *pOsMS1:OsMS1-FLAG* and *pOsMS1:OsMS1*^{wenmin1}*-FLAG* transgenic rice and detected by western blots with antibody against FLAG, H4 or BIP. OsMS1^{wenmin1}-FLAG was detected in the nuclei and cytoplasm. H4 and BIP were used as a marker for nuclei and cytoplasm, respectively. Red arrows indicate nuclei, white arrows indicate cytoplasm (**a-e**). Representative results of at least three independent experiments in **a-f** are shown. Source data are provided as a Source Data file.

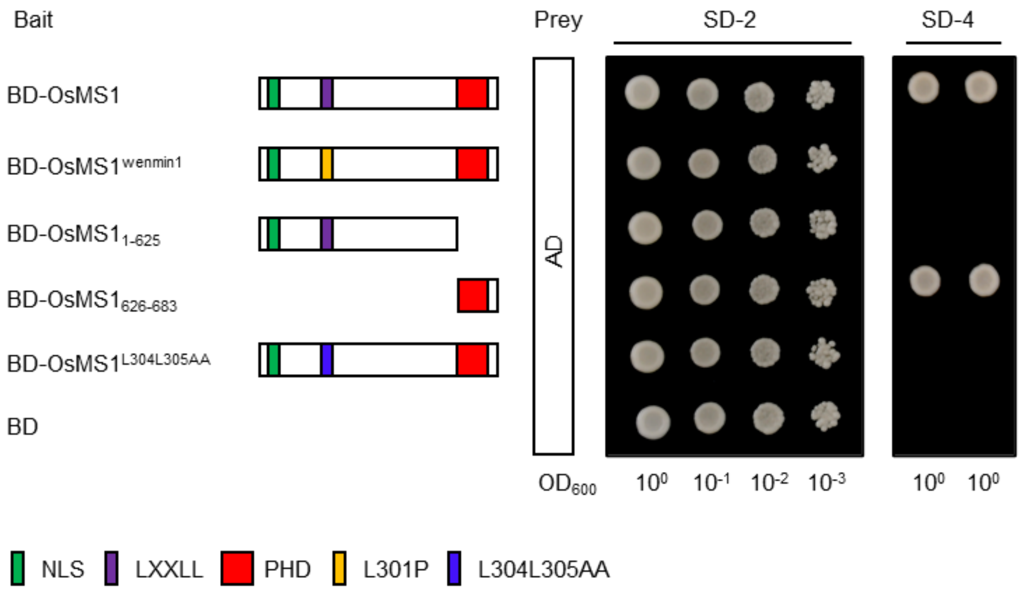

Supplementary Fig. 11. Transcriptional activation assay in yeast.

Full-length OsMS1 and the indicated deletion or mutation constructs were fused in frame with BD and cotransfected with AD. Diploid yeast cells were spotted on the indicated plates and grown at 30°C. BD, GAL4-DNA binding domain; AD, GAL4-activation domain; SD-2, plates lacking Trp and Leu; SD-4, plates lacking Trp, Leu, His, and Ade.

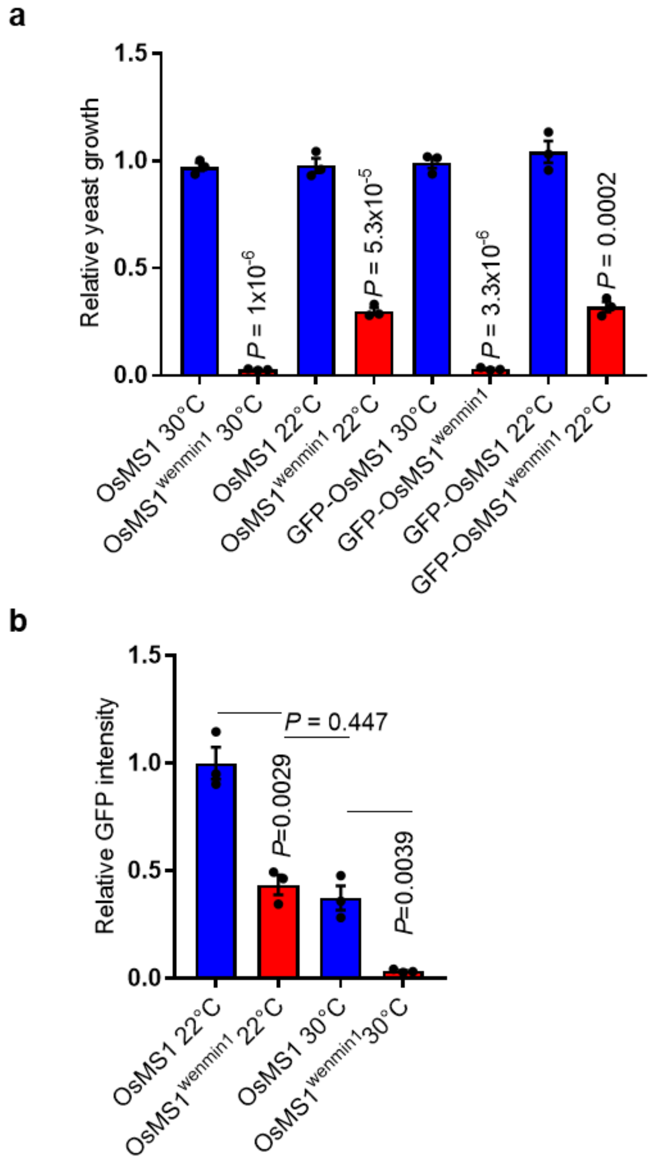

Supplementary Fig. 12. L301 is essential for the transcriptional activation activity and subcellular localization of OsMS1 in yeast.

a, L301 is essential for the transcriptional activation activity of OsMS1 in yeast. Equal numbers of diploid yeast cells were cultured in the SD-2 or SD-3 liquid medium at 22°C (OD_{600}=0.05) and 30°C (OD_{600}=0.01) for the indicated time. Relative yeast growth was shown as (OD_{600} in SD-3)/ (OD_{600} in SD-2). *P* values indicate the significant differences relative to OsMS1 at the indicated temperatures. Values are means ± s.e.m. (n = 3 biological replicates). Two-tailed unpaired *t*-test was used for statistical analysis. **b,** Quantitative analysis of Fig 3b. The GFP-OsMS1 and GFP-OsMS1^{wenmin1} protein levels in nuclei were quantitated by Image J and normalized to every single cell. Values are means ± s.e.m. (n = 3 biological replicates). Two-tailed unpaired *t*-test was used for statistical analysis. Source data are provided as a Source Data file.

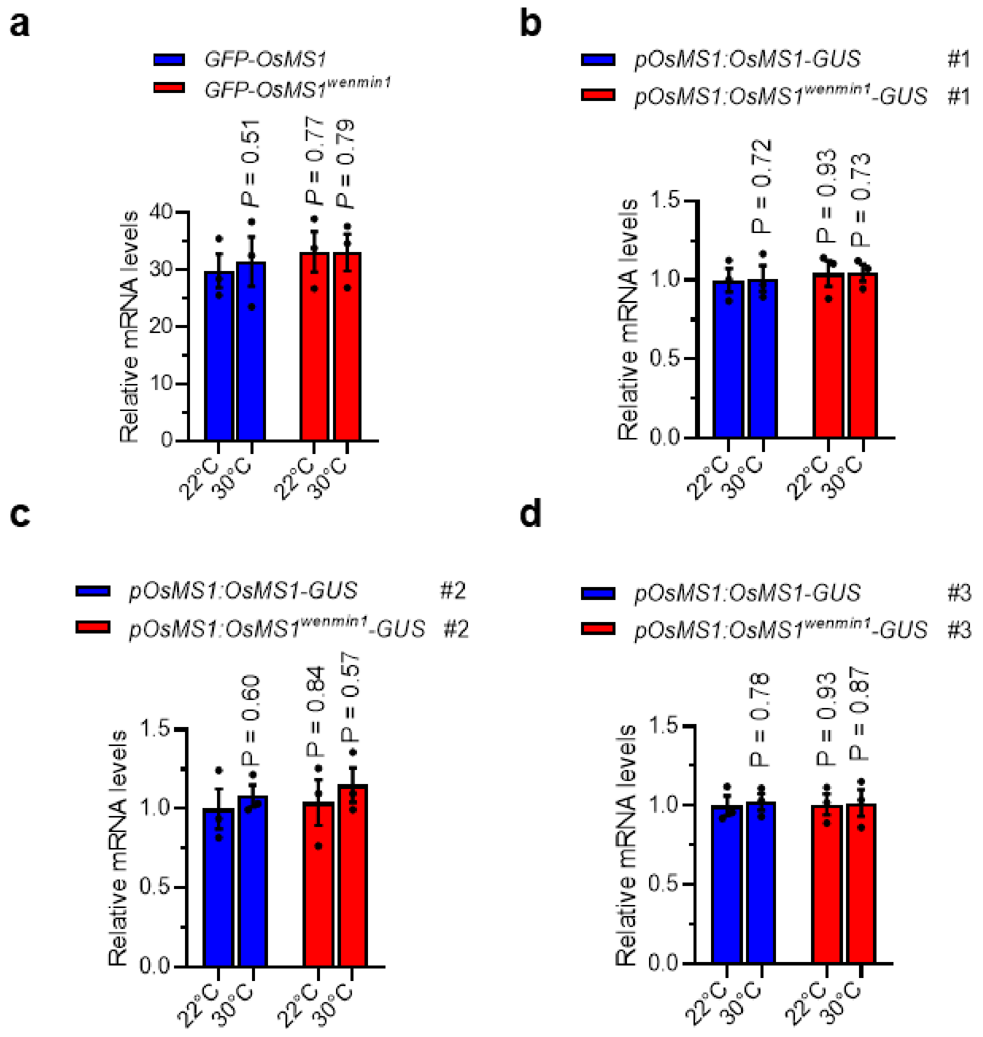

Supplementary Fig. 13. mRNA levels of *OsMS1* and *OsMS1^{wenmin1}*.

a, mRNA levels of *OsMS1* or *OsMS1^{wenmin1}* in *N. benthamiana* plants expressing GFP-OsMS1 and GFP-OsMS1^{wenmin1} (Related to Fig. 3**c**, n = 3 biological replicates). P values indicate the significant differences relative to *OsMS1* at 22°C. **b-d,** mRNA levels of *OsMS1-GUS* and *OsMS1^{wenmin1}-GUS* in transgenic *pOsMS1:OsMS1-GUS* and *pOsMS1:OsMS1^{wenmin1}-GUS* plants (Related to Fig. 3**h**, **i** and **j**). Stage 9 anthers were used. *OsMS1 or OsMS1^{wenmin1}* mRNA levels were normalized against *OsACTIN1* and shown as relative mRNA levels. P values indicate the significant differences relative to *OsMS1* at22°C. In **a-d**, values are mean ± s.e.m. (n = 3 biological replicates), two-tailed unpaired t-test was used for statistical analysis. Source data are provided as a Source Data file.

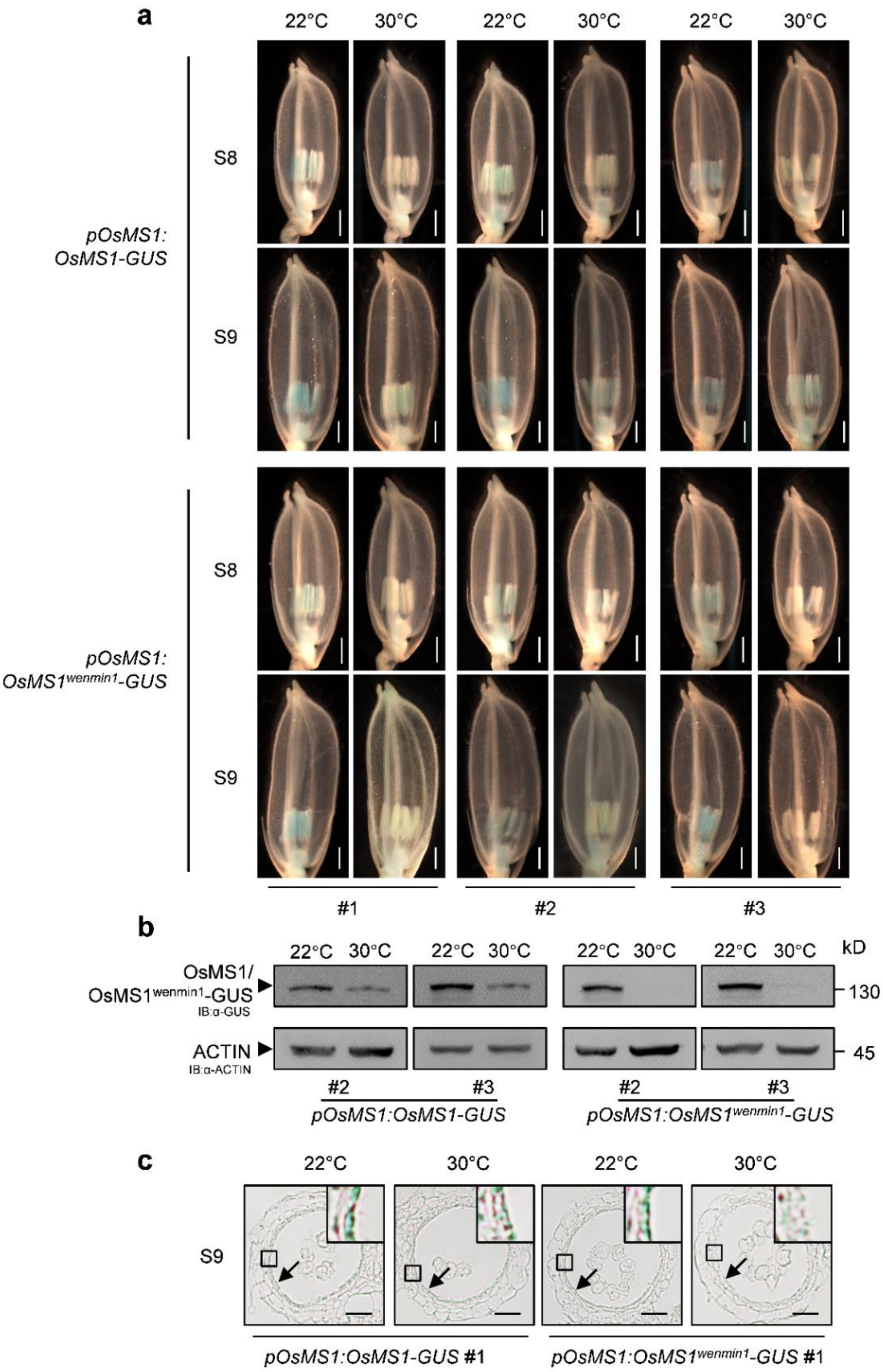

Supplementary Fig. 14. Temperature regulates the abundances of OsMS1 and OsMS1^{wenmin1}

a, Protein levels of OsMS1-GUS and OsMS1^{wenmin1}-GUS are temperature-dependent in rice. *pOsMS1*:*OsMS1*-GUS and *pOsMS1*:*OsMS1^{wenmin1}*-GUS transgenic plants were grown at 22°C or 30°C in growth chamber. S8, stage 8; S9, stage 9. Scale bars, 1 mm. **b**, Protein levels of OsMS1-GUS and OsMS1^{wenmin1}-GUS in stage 9 anthers were

detected by ani-GUS antibody. ACTIN was used as the loading control. *pOsMS1*:*OsMS1-GUS* and *pOsMS1*:*OsMS1*^{wenmin1}*-GUS* transgenic plants were grown at 22°C or 30°C in growth chamber. Black triangles indicate proteins OsMS1-GUS, OsMS1^{wenmin1}-GUS or ACTIN. **c,** Transverse section analysis of OsMS1-GUS and OsMS1^{wenmin1}-GUS in rice anthers. *pOsMS1:OsMS1-GUS* and *pOsMS1:OsMS1*^{wenmin1}*-GUS* transgenic plants were grown at 22°C or 30°C in growth chamber. The images are cross sections of a single locule. Black arrows indicate tapetum. Scale bars, 20 μm. Representative results of at least three independent experiments in **b** and **c** are shown. Source data are provided as a Source Data file.

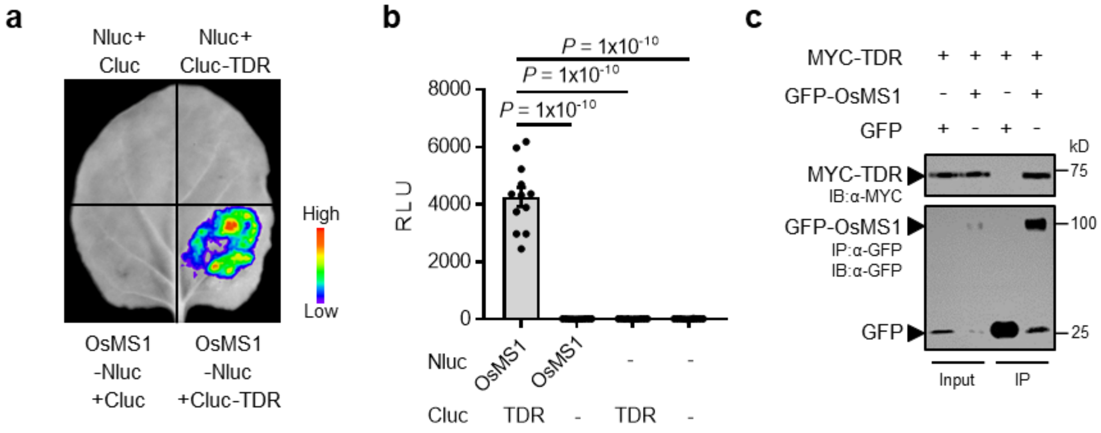

Supplementary Fig. 15. OsMS1 associates with TDR.

a, OsMS1 associates with TDR in *N. benthamiana* plants. Luciferase activity is depicted with false color from low (blue) to high (red). Nluc, N-terminal half of firefly luciferase; Cluc, C-terminal half of firefly luciferase. **b,** Quantitative analysis of (**a**). The protein-protein interaction intensity was shown as RLU. RLU, relative luminescence unit. Values are mean RLU ± s.e.m. (*n* = 12 biological replicates). Two-tailed unpaired *t*-test was used for statistical analysis. **c,** OsMS1 associates with TDR in rice by co-immunoprecipitation analysis. *35S:GFP-OsMS1* and *35S:MYC-TDR* was transformed into ZH11 and hybrid with each other, and subjected to co-immunoprecipitation (IP) with GFP-Trap beads, and then immunoblotted with anti-GFP or anti-MYC antibody. Empty GFP was used as a negative control. IB, immunoblot; IP, immunoprecipitation. Representative results of at least three independent experiments in **c** are shown. Source data are provided as a Source Data file.

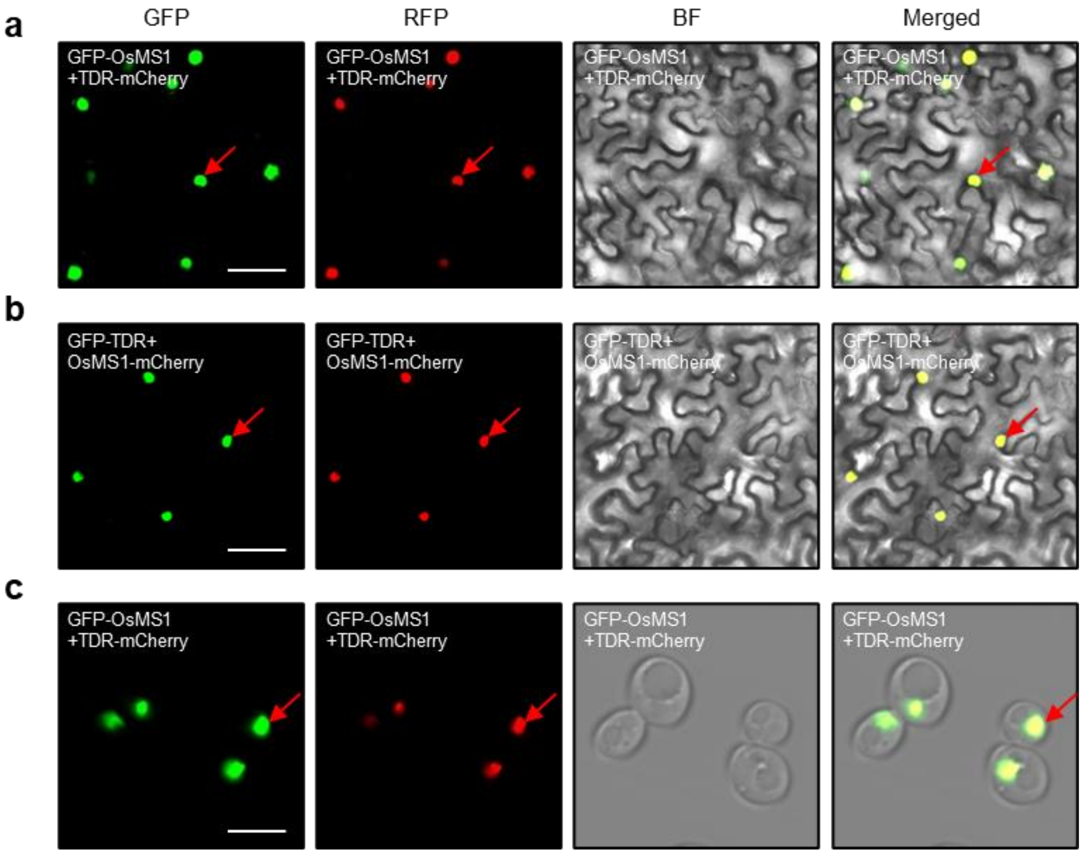

Supplementary Fig. 16. Co-localization of OsMS1 with TDR.

a, Co-localization of GFP-OsMS1 with TDR-mCherry in *N. benthamiana* leaves. GFP-OsMS1 overlapped with TDR-mCherry at the nucleus. Green fluorescence is an indicative of GFP-OsMS1, whereas red fluorescence shows RFP as an indicator of TDR-mCherry. BF, bright field. **b,** Co-localization of OsMS1-mCherry with GFP-TDR in *N. benthamiana* leaves. OsMS1-mCherry overlapped with GFP-TDR at the nucleus. Red fluorescence is indicative of OsMS1-mCherry, whereas green fluorescence shows GFP as an indicator of GFP-TDR. BF, bright field. **c,** Co-localization of GFP-OsMS1 with TDR-mCherry in yeast. GFP-OsMS1 was merged with that of TDR-mCherry at the nucleus. Red arrows indicate nuclei (**a-c**). Scale bars, 50 μm (**a, b**), 5 μm (**c**). Representative results of at least three independent experiments in **a-c** are shown. Source data are provided as a Source Data file.

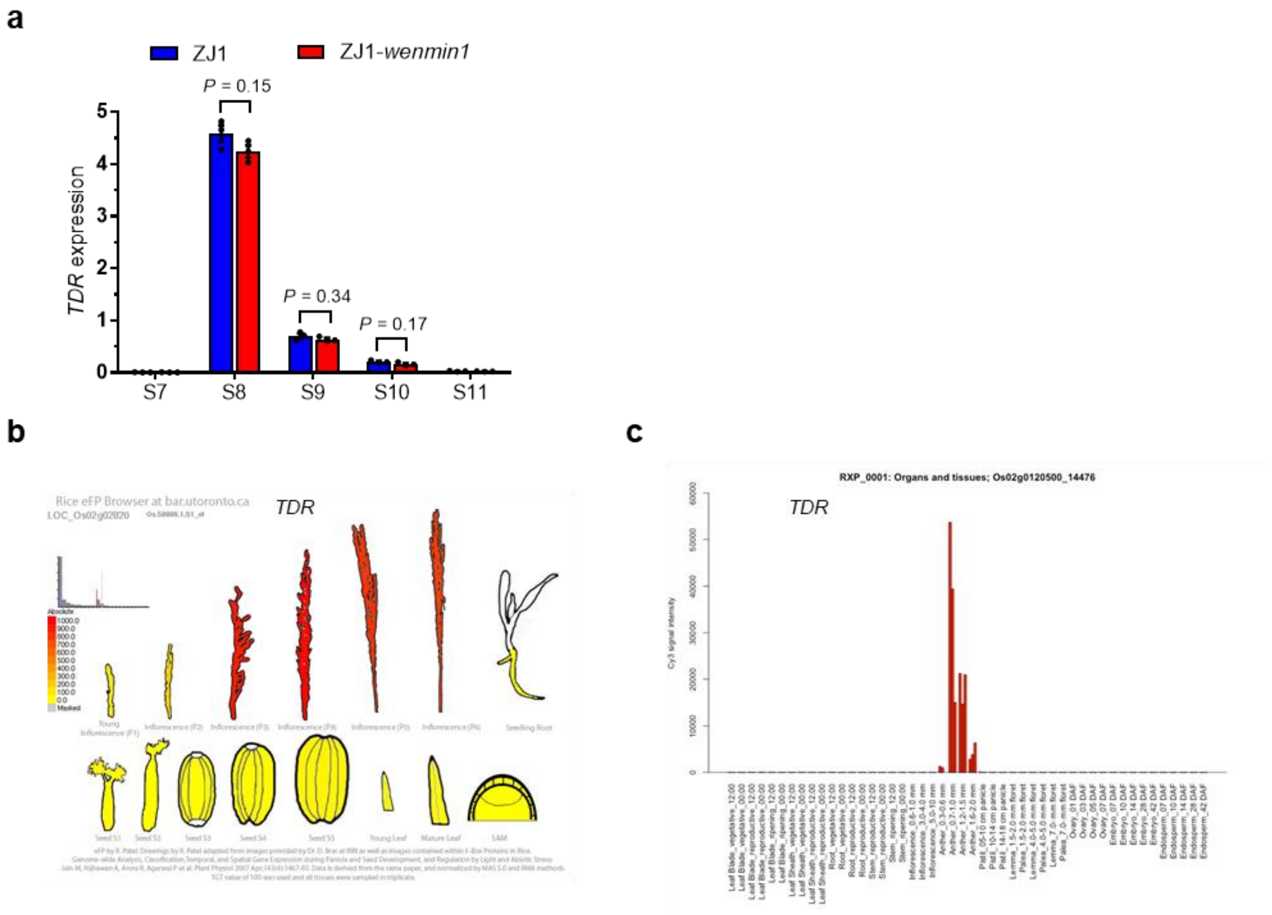

Supplementary Fig. 17. Expression patterns of *TDR*.

a, Expression profiles of *TDR* determined by qPCR analysis. Expression levels were normalized against *OsACTIN1* gene. Values are means ± s.e.m. (n = 3 biological replicates). Two-tailed unpaired t-test was used for statistical analysis. S7, S8, S9, S10, S11 represent stage 7, stage 8, stage 9, stage10, stage 11 during rice anther development, respectively. **b,** Tissue-specific expression patterns of *TDR* obtained from Botany Array Resource (http://bar.utoronto.ca/efprice/cgi-bin/efpWeb.cgi). Signal threshold was set to 1,000 for all the data extraction. **c,** Expression profiles of *TDR* derived from RiceXPro (https://ricexpro.dna.affrc.go.jp/). Source data are provided as a Source Data file.

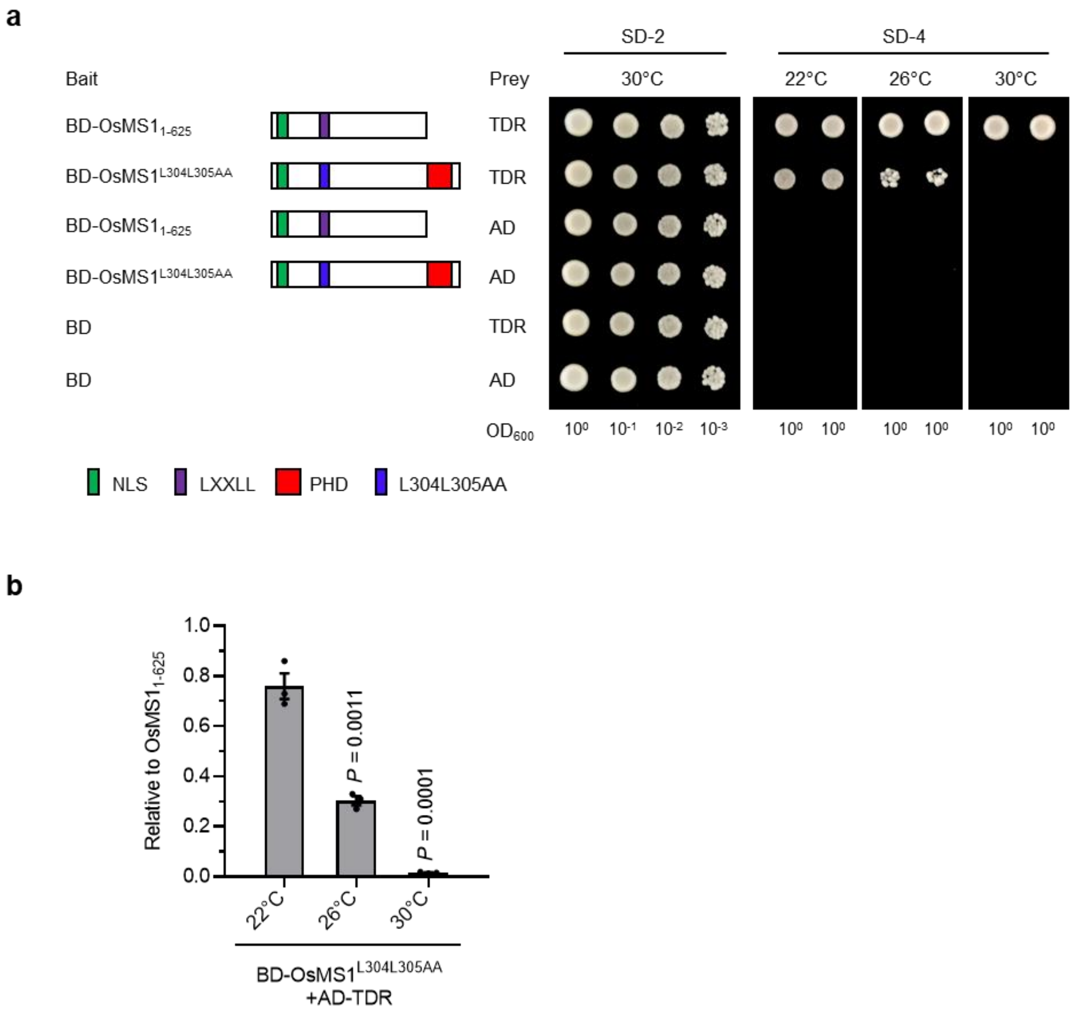

Supplementary Fig. 18. LXXLL motif is essential for temperature-dependent association of OsMS1 and TDR.

a, Temperature-dependent association of OsMS1^{L304L305AA} and TDR in yeast. Yeast two-hybrid assay to test interactions between BD-OsMS1$_{1\text{-}625}$, BD-OsMS1^{L304L305AA} and AD-TDR. Equal numbers of diploid yeast cells (OD_{600}=1) were spotted on the SD-4 plates and grown at 22°C, 26°C, and 30°C for 96 h (**a**). Diluted yeast cells spotted on the SD-2 plates were taken as loading control. **b,** Quantitative analysis of (**a**). yeast cells were cultured in the SD-4 liquid medium at 22°C, 26°C, and 30°C (OD_{600}=0.01) for approximately 96 h. Yeast growth was shown as OD_{600}, and normalized to yeast cells coexpressing BD-OsMS1$_{1\text{-}625}$ and AD-TDR. Values are means ± s.e.m. (n = 3 biological replicates). Two-tailed unpaired t-test was used for statistical analysis. P values indicate the significant differences relative to 22°C. Source data are provided as a Source Data file.

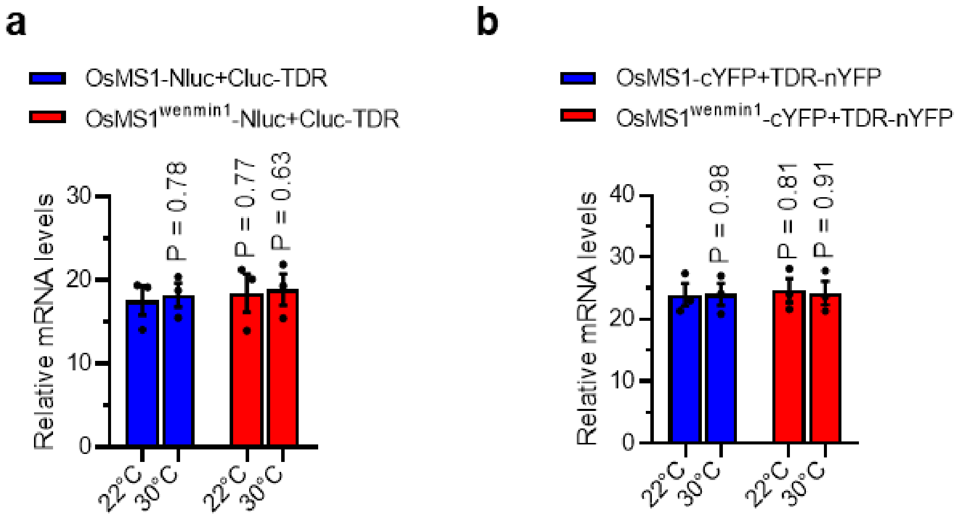

Supplementary Fig. 19. mRNA levels of *OsMS1* and *OsMS1^{wenmin1}* in *N. benthamiana* plants.

a, mRNA levels of *OsMS1* or *OsMS1^{wenmin1}* in *N. benthamiana* plants expressing OsMS1-Nluc + Cluc-TDR and OsMS1^{wenmin1}-Nluc + Cluc-TDR (Related to Fig. 4b, *n* =3 biological replicates). **b**, mRNA levels of *OsMS1* or *OsMS1^{wenmin1}* in *N. benthamiana* plants expressing OsMS1-cYFP + TDR-nYFP and OsMS1^{wenmin1}-cYFP + TDR-nYFP (Related to Fig. 4d, n = 3 biological replicates). *OsMS1* or *OsMS1^{wenmin1}* mRNA levels in (**a**, **b**) were normalized against *NbACTIN1* gene and shown as relative mRNA levels. *P* values indicate the significant differences relative to *OsMS1* at 22°C. Values are mean ± s.e.m, two-tailed unpaired *t*-test was used for statistical analysis. Source data are provided as a Source Data file.

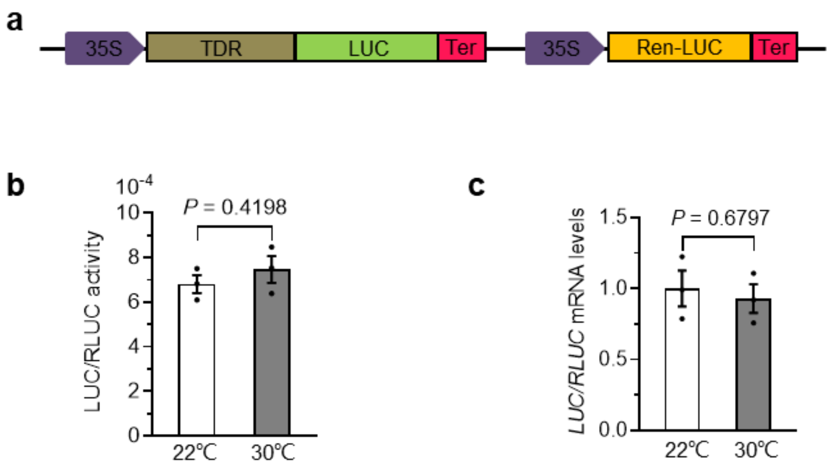

Supplementary Fig. 20. Protein levels of TDR is not temperature-dependent in *N. benthamiana* plants.

a, Dual-luciferase system. LUC, firefly luciferase. Ren-LUC, Renilla luciferase. **b,** Relative luciferase activity of TDR-LUC in *N. benthamiana* plants during the low temperature to high temperature transition. **c,** *TDR* mRNA levels as in (**b**). Means of the *LUC/RLUC* mRNA ratios were normalized to *TDR-LUC/RLUC* at 22°C. Values are means ± s.e.m., n = 3 biological replicates in (**b**) and (**c**). Two-tailed unpaired *t*-test was used for statistical analysis. Source data are provided as a Source Data file.

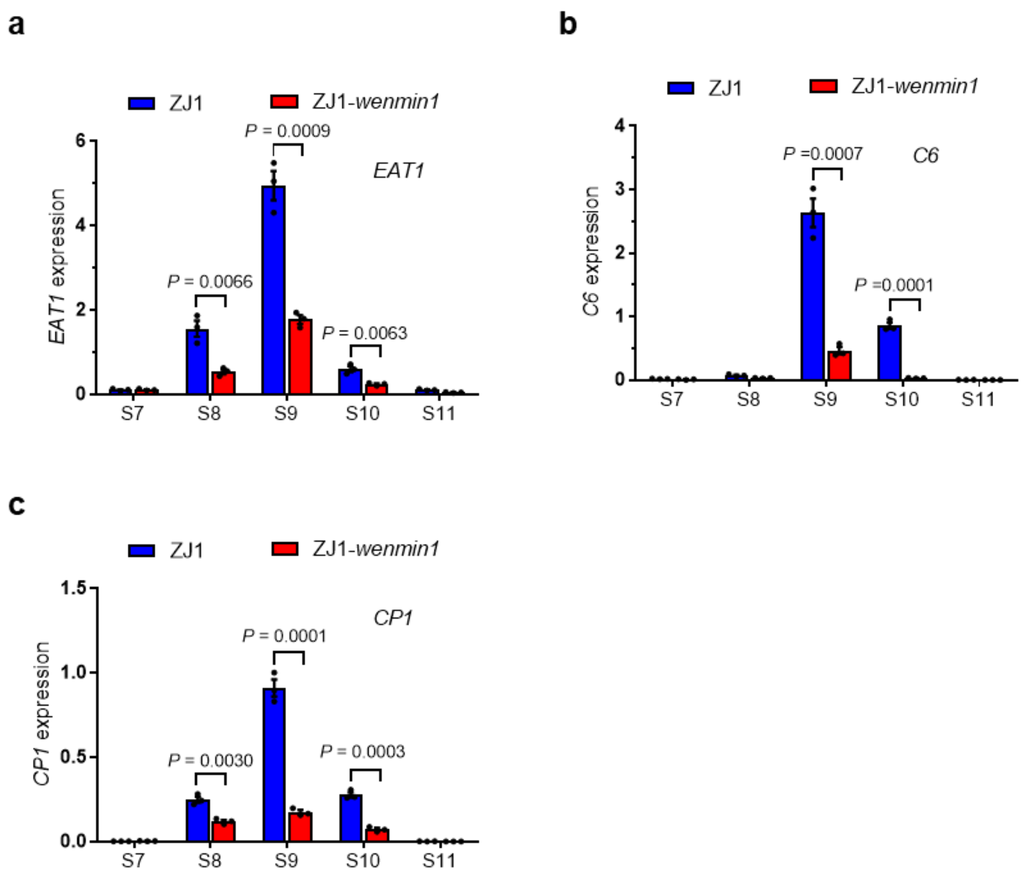

Supplementary Fig. 21. Expression patterns of *C6* and *CP1* in ZJ1 and ZJ1-*wenmin1* under restrictive temperatures.

a, b, c, Expression profiles of *EAT1* (**a**), *C6* (**b**) and *CP1* (**c**) determined by qPCR analysis in ZJ1 and ZJ1-*wenmin1* anthers. Expression levels were normalized against *OsACTIN1*. Values are means ± s.e.m. (n = 3 biological replicates). S7, S8, S9, S10, S11 represent stage 7, stage 8, stage 9, stage10, stage 11 during rice anther development, respectively. Two-tailed unpaired t-test was used for statistical analysis. Source data are provided as a Source Data file.

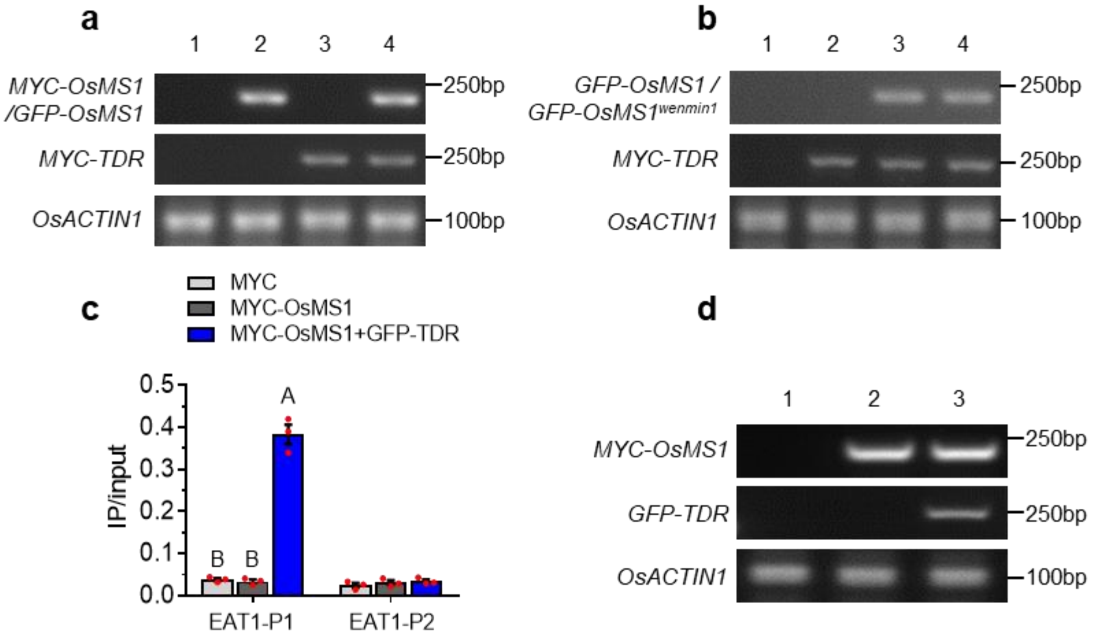

Supplementary Fig. 22. The association of OsMS1 with the *EAT1* promoter depends on TDR (Related to Fig. 5d and 5e).

a, Expressions of *MYC-OsMS1, GFP-OsMS1, MYC-TDR* and *OsACTIN1* in rice leaf protoplasts determined by RT-PCR analysis (Related to Fig. 5**d**). Number 1-4 indicate protoplast expressing *MYC*, *MYC-OsMS1*, *MYC-TDR*, *MYC-TDR+GFP-OsMS1*, respectively. **b**, Expressions of *GFP-OsMS1, GFP-OsMS1*^{wenmin1}*, MYC-TDR* in rice leaf protoplasts determined by RT-PCR analysis (Related to Fig. 5**e**). Number 1-4 indicate protoplast expressing *MYC*, *MYC-TDR*, *MYC-TDR+GFP-OsMS1*, *MYC-TDR+GFP-OsMS1*^{wenmin1}, respectively. **c**, ChIP-qPCR reveals that the association of OsMS1 with the promoter of *EAT1* depends on TDR in rice protoplasts. The fragment P2 was used as negative control (Related to Fig.5a). ChIP-qPCR results were quantified by normalization of the MYC-IP signal with the corresponding input signal (IP/input). Data are presented as means ± s.e.m. (n = 3 biological replicates). Different letters represent significant differences (P<0.01; Duncan's multiple range test). **d**, Expressions of *MYC-OsMS1, GFP-TDR* and *OsACTIN1* in rice leaf protoplasts determined by RT-PCR analysis (Related to **c**). Number 1-3 indicate protoplast expressing *MYC*, *MYC-OsMS1*, *MYC-OsMS1+GFP-TDR*, respectively. In **a**, **b** and **d**, primers within *OsMS1* were used for determining expression levels of *MYC-OsMS1, GFP-OsMS1* and *GFP-OsMS1*^{wenmin1}, primers within *TDR* were used for determining expression levels of *MYC-TDR* and *GFP-TDR* and *OsACTIN1* was used as control. Representative results of at least three independent experiments in **a, b** and **d** are shown. Source data are provided as a Source Data file.

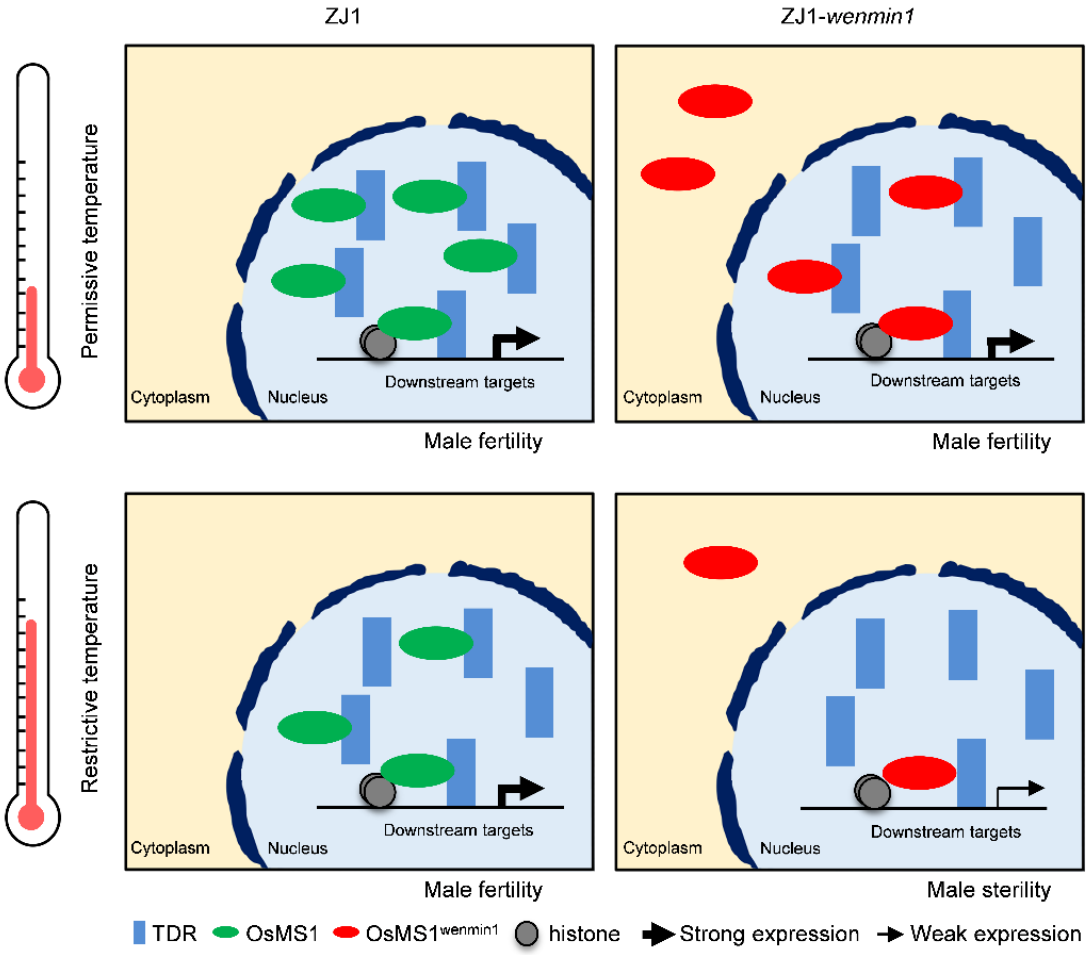

Supplementary Fig. 23. Proposed working model for OsMS1-mediated thermosensitive mechanism.

OsMS1 and OsMS1^{wenmin1} protein levels are regulated in a temperature-dependent manner although their transcriptional levels are similar. OsMS1 is localized in nuclei, while OsMS1^{wenmin1} is localized in both cytoplasm and nuclei, indicating that the mutation in OsMS1^{wenmin1} causes the decreased abundance in nuclei. The temperatures regulate the abundances of OsMS1 and OsMS1^{wenmin1}, and OsMS1^{wenmin1} is more sensitive to temperature changes than OsMS1. At permissive (low) temperatures, both OsMS1 and OsMS1^{wenmin1} interact with TDR to activate the expression of *EAT1*, thereby generating fertile pollens. At restrictive (high) temperatures, the abundance of OsMS1 proteins was decreased, but there are still enough OsMS1 proteins to interact with TDR to activate the expression of *EAT1*, therefore producing fertile pollens. On contrast, the high temperatures strongly reduce the levels of OsMS1^{wenmin1} proteins, thus there are no enough OsMS1^{wenmin1} proteins in nuclei to interact with TDR, resulting in a dramatic decrease of *EAT1* expression and forming sterile or no pollens.

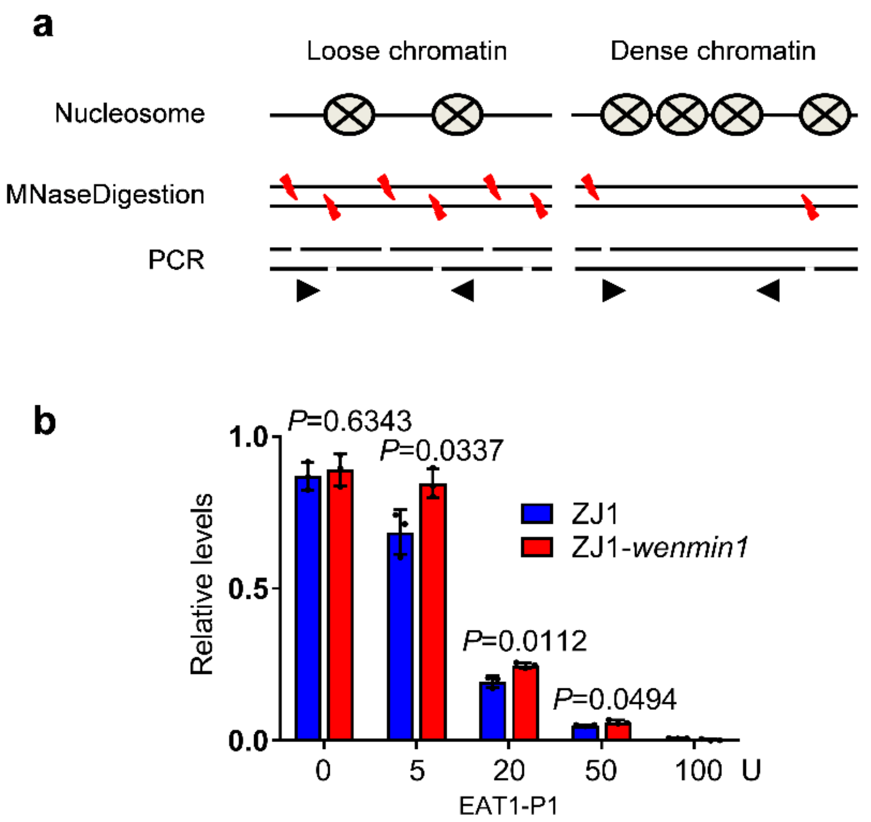

Supplementary Fig. 24. The *OsMS1*^{wenmin1} allele slightly influences the chromatin status of the *EAT1* promoter.

a, Schematic representation of the approach to detect chromatin organization. Chromatin with dense nucleosome occupation is resistant to mononuclease (MNase) digestion and efficiently amplified by qPCR. **b**, Sensitivity of the *EAT1* promoter region P1 to increasing dosage of MNase digestion between ZJ1 and ZJ1-*wenmin1*. Equal aliquot of crude nuclei extracts of ZJ1 and ZJ1-*wenmin1* stage 9 spikelets at 30°C was subject to digestion with increasing MNase amounts at 30°C for 20 min and DNA was extracted using phenol:chloroform treatment followed by ethanol precipitation and used for qPCR analysis. Expression levels were normalized against *OsACTIN1* gene. Values are means ± s.e.m. (n = 3 biological replicates). Two-tailed unpaired *t*-test was used for statistical analysis. Source data are provided as a Source Data file.

Supplementary Table 1. Rice materials developed and used in this study.

**Variety/line**	**MAS/transgene**	**Purpose**
Tian1S	*OsMS1*^{wenmin1} donor, *Oryza sativa* L. ssp. *indica*	Gene cloning, NIL development and Fertility analysis
ZJ1	Recurrent parent, *Oryza sativa* L. ssp. *japonica*	Gene cloning, NIL development and Fertility analysis
PA64	*Oryza sativa* L. ssp. *indica*	Co-segregation
HNS-1	*tms9-1* donor, *Oryza sativa* L. ssp. *indica*	Allelic test
AnS-1, Zhu1S	*tms5* donor, *Oryza sativa* L. ssp. *indica*	Allelic test
AnS-1, Zhu1S, Y58S, C815S, PA64S	known TMS lines, *Oryza sativa* L. ssp. *indica*	Sequencing *OsMS1*^{wenmin1} mutation
93-11	*Oryza sativa* L. ssp. *indica*	OsMS1 donor
ZH11	*Oryza sativa* L. ssp. *japonica*	Transgene receipt variety and preparing rice protoplasts
ZJ1-*wenmin1*	Near-isogenic lines harboring *OsMS1*^{wenmin1} in ZJ1	Fertility analysis and complementation test
gOsMS1:ZJ1-*wenmin1*	Genomic *OsMS1* transgenic lines in ZJ1-*wenmin1*	Complementation test and fertility analysis
pOsMS1:OsMS1-GUS	Promoter of *OsMS1* plus *OsMS1* coding sequence in ZH11	GUS staining assays
pOsMS1:OsMS1^{wenmin1}*-GUS*	Promoter of *OsMS1* plus *OsMS1*^{wenmin1} coding sequence in ZH11	GUS staining assays
35S:GFP-OsMS1	*OsMS1* overexpressing transgenic lines in ZH11 and *Arabidopsis* Col-0	Subcellular localization, protein stability analysis and in planta Co-IP
35S:GFP-OsMS1^{wenmin1}	*OsMS1*^{wenmin1} overexpressing transgenic lines in *Arabidopsis* Col-0	Subcellular localization
35S:MYC-TDR	*TDR* overexpressing transgenic lines in ZH11	In planta Co-IP
35S:GFP-OsMS1-35S: MYC-TDR	Double overexpression lines in ZH11 developed by crossing	In planta Co-IP

Supplementary Table 2. Tian1S was a single-genic recessive mutant.

Cross combination	F_2			$\chi^2_{0.05(3:1)}=3.84$
	Total plants	Fertile plants	Sterile plants	
Tian1S×PA64	381	292	89	0.27

Supplementary Table 3. Temperature profile set of the growth chamber.

DAT	Temperatures in light (°C)					Temperatures in dark (°C)		
	7:00-8:00	8:00-9:00	9:00-12:00	12:00-15:00	15:00-19:00	19:00-21:00	21:00-24:00	0:00-7:00
22	20	22	25	27	25	22	20	18
23	21	23	26	28	26	23	21	19
25	23	25	28	30	28	25	23	21
27	25	27	30	32	30	27	25	23
29	27	29	32	34	32	29	27	25

Photoperiod: 12 hour (h) light and 12 h dark.

Supplementary Table 4. Temperature profile set of the growth chamber.

DAT	Temperatures in light (°C)					Temperatures in dark (°C)			
	7:00-8:00	8:00-9:00	9:00-12:00	12:00-15:00	15:00-17:00	17:00-19:00	19:00-21:00	21:00-24:00	0:00-7:00
22	22	22	25	27	25	25	22	20	19
30	28	30	33	35	33	33	30	28	26

Photoperiod: 10 hour (h) light and 14 h dark or 14 hour (h) light and 10 h dark.

Supplementary Table 5. *OsMS1^{wenmin1}* is allelic to *tms9-1* but not to *tms5*.

Cross combination	Sterile gene	F_1 generation plants			Allelic to *OsMS1^{wenmin1}*
		Total plants	Fertile plants	Sterile plants	
Tian1S×HNS-1	*tms9-1*	23	0	23	yes
Tian1S×AnS-1	*tms5*	31	31	0	no
Tian1S×Zhu1S	*tms5*	18	18	0	no
Tian1S×93-11	-	25	25	0	no

Supplementary Table 6. Peptide sequences used in this study.

**Peptide name**	**Sequence (5'-3')**
Biotin-H2A	AGRGKAIGSSAAKKATSRSSK-GGYK(Biotin)
Biotin-H2B	APKAEKKPAAKKPAEEEPAAE-GGYK(Biotin)
Biotin-H3	ARTKQTARKSTGGKAPRKQLA-GGYK(Biotin)
Biotin-H4	SGRGKGGKGLGKGGAKRHRKV-GGYK(Biotin)
Biotin-H4K5ac	SGRGK(Ac)GGKGLGKGGAKRHRKV-GGYK(Biotin)
Biotin-H4K8Ac	SGRGKGGK(Ac)GLGKGGAKRHRKV-GGYK(Biotin)
Biotin-H4K12Ac	SGRGKGGKGLGK(Ac)GGAKRHRKV-GGYK(Biotin)
Biotin-H4K16Ac	SGRGKGGKGLGKGGAK(Ac)RHRKV-GGYK(Biotin)
Biotin-H3K4me3	ARTK(me3)QTARKSTGGKAPRKQLA-GGYK(Biotin)
Biotin-H3K9Ac	ARTKQTARK(Ac)STGGKAPRKQLA-GGYK(Biotin)
Biotin-H3K14Ac	ARTKQTARKSTGGK(Ac)APRKQLA-GGYK(Biotin)
