# Supplementary material for: A natural allele of OsMS1 responds to temperature changes and confers thermosensitive genic male sterility
Source: Nat Commun. 2022 Apr 19;13:2055. doi: 10.1038/s41467-022-29648-z (PMC9018702; doi:10.1038/s41467-022-29648-z)
Supplement: Supplementary file 2 — Description of Additional Supplementary Files [file 41467_2022_29648_MOESM2_ESM.pdf]

Description of Additional Supplementary Files

File name: Supplementary Data 1

Description: Primers used in this study.
