# Supplementary material for: A natural allele of OsMS1 responds to temperature changes and confers thermosensitive genic male sterility
Source: Nat Commun. 2022 Apr 19;13:2055. doi: 10.1038/s41467-022-29648-z (PMC9018702; doi:10.1038/s41467-022-29648-z)
Supplement: Supplementary file 4 — Reporting Summary [file 41467_2022_29648_MOESM4_ESM.pdf]

nature portfolio

Corresponding author(s): Yunhai Li, Yuehua Luo, Chengcai An

Last updated by author(s): Mar 16, 2022

Reporting Summary

Nature Portfolio wishes to improve the reproducibility of the work that we publish. This form provides structure for consistency and transparency in reporting. For further information on Nature Portfolio policies, see our Editorial Policies and the Editorial Policy Checklist.

Statistics

For all statistical analyses, confirm that the following items are present in the figure legend, table legend, main text, or Methods section.

n/a	Confirmed	
☐	☒	The exact sample size (n) for each experimental group/condition, given as a discrete number and unit of measurement
☐	☒	A statement on whether measurements were taken from distinct samples or whether the same sample was measured repeatedly
☐	☒	The statistical test(s) used AND whether they are one- or two-sided<br>*Only common tests should be described solely by name; describe more complex techniques in the Methods section.*
☒	☐	A description of all covariates tested
☒	☐	A description of any assumptions or corrections, such as tests of normality and adjustment for multiple comparisons
☐	☒	A full description of the statistical parameters including central tendency (e.g. means) or other basic estimates (e.g. regression coefficient) AND variation (e.g. standard deviation) or associated estimates of uncertainty (e.g. confidence intervals)
☐	☒	For null hypothesis testing, the test statistic (e.g. F, t, r) with confidence intervals, effect sizes, degrees of freedom and P value noted<br>*Give P values as exact values whenever suitable.*
☒	☐	For Bayesian analysis, information on the choice of priors and Markov chain Monte Carlo settings
☒	☐	For hierarchical and complex designs, identification of the appropriate level for tests and full reporting of outcomes
☒	☐	Estimates of effect sizes (e.g. Cohen's d, Pearson's r), indicating how they were calculated

Our web collection on statistics for biologists contains articles on many of the points above.

Software and code

Policy information about availability of computer code

Data collection	In this study, no custom-made software or codes/mathematical algorithms were generated by the authors. Pollen grains were photographed using a Leica DM2500 microscope. Fluorescence microscopic images were collected on Zeiss LSM710 confocal microscope or Nikon A1R confocal microscope as indicated in the Methods. Luciferase complementation images were taken on NightOWL II LB983. Western blot images were scanned using Tanon-4500. Luminescence recording was detected in GLOMAX 96 microplate luminometer.A low-light cooled CCD imaging apparatus (NightOWL II LB983 with Indigo software) was used to capture the N. benthamiana image. Tissue-specific expression patterns were obtained from Botany Array Resource (http://bar.utoronto.ca/efprice/cgi-bin/efpWeb.cgi). Expression profiles were derived from RiceXPro (https://ricexpro.dna.affrc.go.jp/).
Data analysis	In this study, no custom-made software or codes/mathematical algorithms were generated by the authors. ImageJ (Version 1.49); Adobe Photoshop CC 2018 (version: 1.9); Zen Black 2009 (Zeiss); GraphPad Prism7 (GraphPad Software);MEGA software (version 5.2) .

For manuscripts utilizing custom algorithms or software that are central to the research but not yet described in published literature, software must be made available to editors and reviewers. We strongly encourage code deposition in a community repository (e.g. GitHub). See the Nature Portfolio guidelines for submitting code & software for further information.

Data

Policy information about availability of data

All manuscripts must include a data availability statement. This statement should provide the following information, where applicable:

- Accession codes, unique identifiers, or web links for publicly available datasets
- A description of any restrictions on data availability
- For clinical datasets or third party data, please ensure that the statement adheres to our policy

The authors declare that all data supporting the findings of this study are available within the article and its Supplementary Information files. Sequence details for rice genes used in this study can be found in China Rice Data Center (https://www.arabidopsis.org/) with the following accession numbers: LOC_Os09g27620 (OsMS1), LOC_Os02g02820 (TDR) and LOC_Os04g51070 (EAT1). Tissue-specific expression patterns were obtained from Botany Array Resource (http://bar.utoronto.ca/efprice/cgi-bin/efpWeb.cgi). Expression profiles were derived from RiceXPro (https://ricexpro.dna.affrc.go.jp/). The primers used in this study are provided as Supplementary Data 1. The raw images of microscopy data and uncropped blots or gels are provided in the Source Data file. Further materials are available from the corresponding authors upon reasonable request. Source data are provided with this paper.

Field-specific reporting

Please select the one below that is the best fit for your research. If you are not sure, read the appropriate sections before making your selection.

☒ Life sciences ☐ Behavioural & social sciences ☐ Ecological, evolutionary & environmental sciences

For a reference copy of the document with all sections, see nature.com/documents/nr-reporting-summary-flat.pdf

Life sciences study design

All studies must disclose on these points even when the disclosure is negative.

Sample size	Sample sizes were determined based on empirical knowledge, instructions for commercial kits and previous publications in related research field. For pollen fertility analysis, 100 anthers of 20 mature spikelets from four plants were sampled (based on Zhou H, et al., 2014, reference 5 in the manuscript). For statistical analysis of Fig. 1a in Fig. 1b, ten repeats of pollen fertility experiments were conducted for Tian1S and ZJ1-wenmin1, each with ten anthers being examined. For transverse section analysis of anthers, 100 spikelets at different developmental stages were collected and embedded as described previously (based on Li N, et al., 2006, reference 16 in the manuscript). For quantitative real-time PCR (qPCR) analysis, sufficient stage 9 anthers or protoplast were prepared to extract > 2 μg mRNA for each biological replicate (based on instructions for the TIANGEN, Cat. No: DP439-H and Liu Z et al. 2020, reference 57 in the manuscript). For co-immunoprecipitation assay in Fig.2e, around 2 g of ground frozen stage9 anthers was used for each biological replicate (based on Huang K et al. 2017, reference 65 in the manuscript). For other total RNA and protein prep including N. benthamiana, around 500 mg ground frozen powder were used for each biological replicate (based on Liu Z et al. 2020, reference 57 in the manuscript). For spikelets phenotypes, 4 or more individual plants were analyzed, detailed in relevant figure legends (based on Zhou H, et al., 2014, reference 5 in the manuscript).For chromatin immunoprecipitation (ChIP) and qPCR, approximately 10ml protoplasts or 2 g young panicles with stage 9 anthers were used.
Data exclusions	No data was excluded from the analyses.
Replication	To ensure the reliability of experimental results, we had at least three biological replicates for each experiment, which are described in the relevant figure legends. The key experiments were reproduced at least three times with similar results, as indicated in the figure legends. The original data for these replicates are provided in the source data file.
Randomization	Arabidopsis thaliana and N. benthamiana plants were grown on soil and randomly placed on the same shelf in the growth chamber. For field trials, rice plants were randomly grown in the same open field. Plants of different genotypes were collected randomly.
Blinding	Researchers were blinded during fluorescence microscopic images collection and analysis. Researchers were not blinded to plant genotypes during experiments. Routine practices included more than one author observing/assessing phenotypes, whenever possible. Experiment results are not subjective.

Reporting for specific materials, systems and methods

We require information from authors about some types of materials, experimental systems and methods used in many studies. Here, indicate whether each material, system or method listed is relevant to your study. If you are not sure if a list item applies to your research, read the appropriate section before selecting a response.

Materials & experimental systems

n/a	Involved in the study
	[x] Antibodies
[x]	Eukaryotic cell lines
[x]	Palaeontology and archaeology
[x]	Animals and other organisms
[x]	Human research participants
[x]	Clinical data
[x]	Dual use research of concern

Methods

n/a	Involved in the study
[x]	ChIP-seq
[x]	Flow cytometry
[x]	MRI-based neuroimaging

Antibodies

Antibodies used

The following antibodies were used for western blot, immunoprecipitation or ChIP-PCR:
Anti-FLAG (#M20008, dilution, 1:5000), anti-GST (#M20007, dilution, 1:5000), anti-GFP (#M20004, dilution, 1:5000), anti-MYC (#M20002, dilution, 1:5000), anti-MBP (#M20051, dilution, 1:5000), anti-GUS (#P26299, dilution, 1:2000), and anti-ACTIN(#M20009, 26F7, dilution, 1:5000) were purchased from Abmart (Shanghai, China).
Anti-MYC (#ab32, dilution, 1:200), anti-FLAG (#ab205606, dilution, 1:200) and anti-BIP (#ab108615, dilution, 1:5000) were purchased from Abcam (Cambridge, UK).
Anti-H4 (#61521, Clone: MABI 0400, dilution, 1:5000) was purchased from Active Motif (Carlsbad, USA).
Streptavidin agarose beads (#16-126), Glutathione SepharoseTM 4B (#45-000-139), GFP-Trap® agarose (#gta-20) and one step western kit HRP (#CW2030) were purchased from Sigma-Aldrich (St Louis, USA), GE Healthcare(Chicago, IL, USA), ChromoTek (Planegg-Martinsried, Germany), and Cwbiotech (Beijing, China), respectively.
The antibodies were applied in ChIP-qPCR at 15 μg per ChIP.

Validation

Validation statements, relevant citations of commercial primary antibodies are available from manufacturers:
Anti-FLAG antibody (#M20008) is a mouse monoclonal antibody to the synthetic DYKDDDDK peptide (KLH-coupled). http://www.ab-mart.com.cn/page.aspx?node=%2060%20&id=%20968
Anti-GST is a mouse monoclonal antibody to Glutathione S-Transferase (GST). http://www.ab-mart.com.cn/page.aspx?node=%2060%20&id=%20967
Anti-GFP antibody is a mouse monoclonal antibody to full length recombinant GFP. http://www.ab-mart.com.cn/page.aspx?node=%2060%20&id=%20971
Anti-MYC antibody (#M20002) is a mouse monoclonal antibody to a synthetic peptide (KLH-coupled) corresponding to residues 410-419 of human c-Myc (EQKLISEEDL). http://www.ab-mart.com.cn/page.aspx?node=%2060%20&id=%20962
Anti-MBP is produced by immunizing animals with full-length MBP protein. http://www.ab-mart.com.cn/page.aspx?node=%2060%20&id=%2017734
Anti-GUS antibody is produced by immunizing rabbit. http://www.ab-mart.com.cn/page.aspx?node=%2060%20&id=%2049655
Anti-ACTIN (#M20009, 26F7) is produced by immunizing mice with Arabidopsis ACT11 full-length protein. http://www.ab-mart.com.cn/page.aspx?node=%2059%20&id=%20985
Anti-MYC antibody (#ab32) is a mouse monoclonal antibody to MYC tag. https://www.abcam.cn/myc-tag-antibody-9e10-ab32.html
Anti-FLAG antibody (#ab205606) is a rabbit monoclonal antibody to DDDDK tag (Binds to FLAG® tag sequence). https://www.abcam.cn/ddddk-tag-binds-to-flag-tag-sequence-antibody-epr20018-251-ab205606.html
Anti-BIP (#ab108615) is a recombinant rabbit monoclonal [EPR4041(2)] to GRP78 BiP. https://www.abcam.cn/grp78-bip-antibody-epr40412-ab108615.html
Anti-H4 antibody is a mouse monoclonal antibody to a synthetic peptide containing human Histone H4. https://www.activemotif.com/catalog/details/61521/histone-h4-antibody-mab-clone-mabi-0400
Streptavidin isolated from Streptomyces avidinii covalently bound to agarose. Suitable for purification of biotinylated macromolecules. https://www.sigmaaldrich.com/catalog/product/mm/16126?lang=zh&region=CN
Glutathione Sepharose 4B is designed for high capacity single-step purification of Glutathione S-Transferase (GST) tagged fusion proteins. https://www.fishersci.com/shop/products/ge-healthcare-glutathione-sepharose-4b-media-3/45000139
GFP-Trap® Agarose is an affinity resin for immunoprecipitation of GFP-fusion proteins, consisting a GFP Nanobody/ VHH coupled to agarose beads. https://www.chromotek.com/products/detail/product-detail/gfp-trap-agarose/
One step western kit HRP is a product for western blot through incubating with primary antibody. https://www.cwbiotech.com/goods/index/id/10294
